# Using Joint Models in Phase I Dose‐Finding Designs in Oncology: Considerations for Frequentist Approaches

**DOI:** 10.1002/sim.70758

**Published:** 2026-10-13

**Authors:** Xijin Chen, Pavel Mozgunov, Richard D. Baird, Thomas Jaki

**Affiliations:** ^1^ MRC Biostatistics Unit University of Cambridge Cambridge UK; ^2^ Department of Oncology University of Cambridge Cambridge UK; ^3^ Computational Statistics Group University of Regensburg Regensburg Germany

**Keywords:** biomarker information, CRM likelihood, existence of MLE, factorization, mixed outcomes, separation condition

## Abstract

Dose‐finding trials for oncology studies are traditionally designed to assess safety in the early stages of drug development. With the rise of molecularly targeted therapies and immuno‐oncology compounds, biomarker‐driven approaches are increasingly importance. In this article, we propose a novel approach to dose‐finding that incorporates biomarkers which predict for the subsequent development of clinical toxicity. The proposed joint model framework, which incorporates repeated biomarker measurements, is compared to the likelihood‐based continual reassessment method (CRM) using only binary toxicity data, across various dose–toxicity relationship scenarios. Our findings highlight a critical limitation of likelihood‐based approaches in early‐phase dose‐finding studies with small sample sizes: estimation challenges that have been previously overlooked in the phase I dose‐escalation setting. We explore potential remedies to address these challenges and emphasize the appropriate use of likelihood‐based methods. Simulation results demonstrate that the proposed joint model framework, by integrating biomarker information, can alleviate estimation problems in the likelihood‐based CRM and improve the proportion of correct selection. However, we highlight that the inherent data limitations in early phase dose‐finding studies remain a significant challenge that cannot be fully overcome in the frequentist framework.

## 1 Introduction

Dose‐finding studies, particularly phase I trials, primarily aim to assess toxicity before evaluating efficacy and comparing new treatments with a standard‐of‐care in subsequent phases. These trials are traditionally designed for cytotoxic agents, being based on tumor size and histology. The advent of molecularly targeted therapies and immuno‐oncology compounds has revolutionized oncology drug development [1]. Regulatory agencies like the Food and Drug Administration (FDA) champion innovations of modern approaches, such as master protocol, adaptive designs, and biomarker‐driven evaluations for patient selection and endpoint determination [2]. The US FDA's is driving a more thorough determination of optimal dose at an earlier point in clinical drug development [3].

Biomarkers, which are defined as objectively measurable indicators of biological or pharmacological processes, play a central role in the evolution of paradigm for dose selection [4]. Modern technologies such as liquid biopsies provide a minimally invasive approach to capturing the comprehensive genetic landscape of tumors in real time throughout the course of treatment [5].

Under the FDA‐NIH BEST framework, a safety biomarker is measured during treatment to indicate the likelihood or extent of toxicity as an adverse event. Although such biomarkers are collected longitudinally and share features with monitoring biomarkers [6, 7], our approach is distinct: the repeated measurements serve as prospective predictive signals to inform dose‐escalation decisions within the trial, rather than to track cumulative or long‐term organ damage across treatment cycles.

Prominent examples include the absolute neutrophil count (ANC), routinely measured on a continuous scale throughout a treatment cycle and shown to be an effective predictor of subsequent neutropenic events—among the most common dose‐limiting toxicities in oncology trials. In particular, nadir ANC in the first chemotherapy cycle has been shown to predict subsequent neutropenic complications in adjuvant chemotherapy, with patients below a critical threshold experiencing a threefold higher rate of febrile neutropenia in later cycles [8]. Serial measurements of liver enzymes such as ALT and AST during treatment cycles are routinely used to monitor for hepatotoxicity, though their prognostic utility in acute settings remains limited [9], suggesting that emerging biomarkers may ultimately provide more reliable early signals within such a framework. In certain tumor types, platelet counts have also emerged as a candidate biomarker for assessing disease recurrence and forecasting treatment‐related toxicity or adverse events [10]; an early decline in platelet count has been shown to predict persistent thrombocytopenia after peptide receptor radionuclide therapy [11]. While some of these biomarkers, such as ANC, are already routinely collected in clinical practice, others remain investigational and require further validation before integration into adaptive trial frameworks.

For efficacy endpoints, circulating tumor DNA (ctDNA) has shown considerable promise in monitoring the therapeutic response, identifying resistance mechanisms, and predicting early relapse in certain tumor types [12, 13, 14]. Advances in next‐generation sequencing (NGS) technologies are likely to expand the repertoire of candidate biomarkers further [15].

These developments underscore the potential of using predictive biomarkers, particularly in dose‐finding studies, where real‐time assessment of treatment‐related outcomes could improve the precision and adaptability of dose selection in oncology phase I trials.

Incorporating a generic biomarker as a predictive marker into dose‐finding designs necessitates the joint modeling of mixed outcomes measured on different scales. For example, Chen et al. [16] incorporate ctDNA as a predictive biomarker for efficacy by dichotomizing the continuous biomarker based on a threshold value, thereby facilitating its integration into the trial design. However, this dichotomization approach may result in a loss of information, potentially reducing the statistical efficiency and predictive power of the biomarker.

Traditional dose‐finding studies have primarily aimed to identify the maximum tolerated dose (MTD) based on dose‐limiting toxicities (DLTs)—serious adverse events that impair normal activities and require therapeutic intervention [17]. However, with many novel oncology agents, DLTs may be infrequent or absent, prompting a shift toward biomarker‐driven dose escalation strategies. While DLTs are often analyzed as a binary endpoint in dose‐finding studies, biomarkers such as platelets are repeatedly measured on a continuous scale. Standard multivariate tools suffice for outcomes measured on the same scale, but jointly analyzing non‐commensurate outcomes requires specialized statistical methods [18]. There are basically two classes of general likelihood‐based approaches to jointly model binary and continuous outcomes [19]. The first class considers a factorization of the joint likelihood and fits a model to each component of the factorization [20, 21, 22]. The other class uses a latent variable shared by both outcomes, inducing the correlation [23, 24].

The concept of factorizing a joint model into a marginal model and a conditional model for different components of mixed outcomes is particularly useful in dose‐finding studies, especially when the mixed outcomes are not always observed simultaneously. Bekele and Shen [25] proposed a joint model for binary toxicity and continuous activity outcomes by factorizing the joint distribution into a marginal model for the continuous outcome and a conditional model for the binary outcome, assuming both outcomes are evaluated at the same time. Wason and Seaman [26] proposed a method for making inferences on composite or mixed efficacy outcomes by fitting a joint model to the continuous and binary efficacy components in phase II cancer studies. This was further expanded to a multivariate probit model incorporating toxicity outcomes in seamless phase I/II studies [27]. However, neither approach has been comprehensively evaluated in adaptive dose‐finding studies involving multiple continuous outcomes assessed over varying evaluation windows. Our proposed method addresses this gap by accommodating potential continuous biomarkers, such as platelet counts, measured on different time scales, thus improving the flexibility and applicability of joint modeling in complex clinical trial settings. Specifically, we focus on phase I dose‐finding trials, modeling both toxicity and predictive biomarkers associated with clinical toxicity outcomes. The use of biomarkers as surrogate endpoints for toxicity would require establishing validated thresholds and demonstrating that the biomarker adequately captures the clinical toxicity of interest—requirements that are difficult to satisfy in the early‐phase setting where toxicity profiles may be incompletely characterized. In our approach, however, the biomarker need not serve as a perfect surrogate for all DLT components. Rather, it acts as a complementary covariate that enhances dose‐toxicity estimation when it demonstrates temporal correlation with DLT occurrence, while binary DLT definitions are retained as the primary endpoint in line with established clinical practice and regulatory frameworks. Since the true correlation between a continuous biomarker and a composite DLT endpoint is unknown in practice and may vary considerably across settings, we evaluate our framework across a range of correlation strengths to assess performance under weak, moderate, and strong correlation assumptions.

Among model‐based approaches for dose‐finding designs, the continual reassessment method (CRM) stands as one of the most widely recognized model‐based phase I trial designs [28]. Originally developed within a Bayesian framework, it was later adapted into a frequentist variant known as the CRM likelihood approach (CRML) [29]. The CRML method has the notable advantage of eliminating the need for prior specification. Under appropriate regularity conditions and with adequate dose diversity, Shen and O'Quigley [30] established that ML estimators in the CRM are consistent and asymptotically normal, providing theoretical justification for their use in sequential dose‐finding trials. These results were later extended by Cheung and Chappell, who developed methods to evaluate model sensitivity and relax certain regularity conditions [31], and by O'Quigley [32], who extended the theoretical framework to two‐parameter models and investigated the impact of subject heterogeneity. These theoretical developments suggest that likelihood‐based CRM methods should perform well in practice, particularly when dose allocation generates sufficient information across the dose range. Further investigations via simulation studies into both Bayesian and likelihood‐based CRM methods have confirmed their practical utility [33]. While Bayesian CRM offers flexibility through the incorporation of prior information, this can introduce sensitivity to prior specification—particularly problematic in early‐phase settings where reliable prior information is scarce. The likelihood‐based approach avoids this dependence while retaining strong theoretical guarantees, making it a transparent and robust choice for the proposed framework.

Despite the potential advantages of ML estimation in dose‐finding designs, practical implementation reveals important numerical challenges that have received limited attention in the literature. This work is organized around two interconnected themes that address critical gaps in current methodology: (i) incorporating biomarker information to enhance dose‐finding decisions, and (ii) investigating numerical issues with maximum likelihood estimation in dose‐finding designs.

First, we introduce a factorization framework that incorporates repeated biomarker measurements taken at different time points along with binary toxicity outcomes in phase I dose‐finding studies. We then compare two variants of the proposed joint method: the Joint2d method, which utilizes a single biomarker measurement, and the Joint9d method, which integrates multiple biomarkers collected across multiple time points. These methods are compared with the traditional probit method, which is based solely on binary toxicity outcomes. Our findings aim to demonstrate the potential of biomarkers to refine dose‐finding methodologies in oncology, particularly by improving the accuracy of dose selection by including a repeatedly measured biomarker (such as platelet counts).

In the following sections, we outline the factorization framework in Section 2, illustrate challenges in parameter estimation in Section 3, propose different remedies in Section 4, and present simulation results in different scenarios in Section 5 to highlight the advantages of incorporating biomarker in phase I dose‐finding studies in oncology. The article concludes with a discussion presented in Section 6.

## 2 Methods

### 2.1 Model‐Based Dose‐Finding Designs Based on the Binary Outcome Only

We consider a dose‐finding trial with J doses under investigation, notated as $d_{1},...,d_{J}$. Each patient enrolled in the trial is assessed for a binary toxicity outcome, denoted as $Y_{b}$, which indicates the presence or absence of DLT at these doses.

To model the dose–toxicity relationship, a working model ψ(x,β) can be employed, where β parameterizes the relationship. An important point about the CRM is that the dose labels are not the actual doses administered, but rather are defined on a conceptual scale that represents an ordering of the risks of events, termed the skeleton [34]. The impact of dose mapping on the performance of the CRM has been evaluated [31, 35]. “Backward fitting,” which is widely accepted in the CRM literature, maps the actual doses from the numerical dose levels through the functional form that best mirrors that shape. That is, dose labels x are transformed from the skeleton using backward substitution into the model. Specifically, these labels are derived by solving $x=\psi^{-1}(p_{0},\beta^{*})$, where $\beta^{*}$ represents the initial guess of the parameter β, and $p_{0}$ corresponds to the skeleton probabilities for the investigated doses. This transformation ensures that the model aligns accurately with the guess about toxicity probability estimates provided by clinicians, creating a strong foundation for dose‐toxicity evaluation.

Table 1 presents examples of candidate models ψ(x,β) and the corresponding dose labels $x_{j}$ associated with the actual doses $d_{j}$ in the set $d_{1},\ldots ,d_{J}$. Further details regarding model choice and the design parameters of the CRM approach can be found in Wheeler et al. [36].

**TABLE 1:** Common choices for dose–toxicity models and resultant dose labels for the CRM.

Model	ψ(x,β)	Dose labels $x_{j}$	Choice of $\beta^{*}$	Dose labels $x_{j}$
	(General form)		(Initial guess)	(Given $\beta^{*}$)
Power (Empiric)	$x^{\exp(\beta_{b1})}$	$p_{j}^{\frac{1}{\exp(\beta_{b1})}}$	$\beta_{b1}=0$	$p_{j}$
One‐parameter Probit	$\Phi (a_{0}+\exp(\beta_{b1})x)$	$\frac{\Phi^{-1}(p_{j})-a_{0}}{\exp(\beta_{b1})}$	$\beta_{b1}=0$	$\Phi^{-1}(p_{j})-a_{0}$
Two‐parameter Probit	$\Phi (\beta_{b0}+\exp(\beta_{b1})x)$	$\frac{\Phi^{-1}(p_{j})-\beta_{b0}}{\exp(\beta_{b1})}$	$\beta_{b0}=0,\;\beta_{b1}=0$	$\Phi^{-1}(p_{j})$

*Note:* $p_{j}$ is the skeleton probability at the dose label $x_{j}$ and $a_{0}$ is the fixed intercept in corresponding one‐parameter models. To ensure a monotonically increasing dose–toxicity curve, the model is parameterized with a positive slope via $\exp(\beta_{b1})$.

### 2.2 Dose‐Finding Algorithm: The Two‐Stage Design

The dose‐finding algorithm follows that of the CRML [29] that focuses on the evaluation of toxicity. The CRML utilizes the benefit of the two‐stage design first proposed by Storer [37] in the context of the classical up‐and‐down schemes and limits its attention to the power model. Specifically, the design comprises two distinct phases: an initial exploratory escalation stage (Stage 1) and a subsequent modeling stage (Stage 2) for refining dose selection. These two phases operate independently of one another.

In Stage 1, the heterogeneity requirement is imposed, necessitating the observation at least one DLT and one non‐DLT [29]. This stage plays a critical role in the context of sequential likelihood estimation, as the likelihood equation remains monotonic in the unknown parameter space until heterogeneity in responses is observed. Meeting this requirement was intended to ensure that the maximization of the log‐likelihood occurs within the interior of the parameter space, thereby enabling more accurate parameter estimation.

The requirement for heterogeneity is well‐established in the literature and originates from the CRML approach to ensure the existence of a maximum likelihood estimate (MLE) for the chosen power model. This requirement originates from more general situations and richer models of two‐parameter logistic model, which has been extensively studied in Silvapulle [38]. Albert and Anderson [39] also identified a certain degree of overlap in the data as a necessary and sufficient condition for the MLE to exist. Similarly, Lesaffre and Kaufmann [40] established a corresponding condition for the probit model, which aligns with the logistic model framework. This condition, known as the *separation condition*, requires a degree of intermixed or overlapping data points to ensure MLE existence in both logistic and probit models.

In binomial response models, data configurations are mutually exclusively categorized as complete separation, quasi‐complete separation, or overlap. Table 2 presents a 2 × 2 tabular representation of the data configurations, specifically tailored to the initial stage starting from the lowest dose in phase I dose‐finding context. This representation focuses on the two lowest dose levels to reflect conditions typically encountered at the beginning of such studies.

**TABLE 2:** Three mutually exclusive binary outcome configurations at the initial stage of dose‐finding studies with two dose levels.

	DLTs	Non‐DLTs
(a) Complete separation		
$d_{1}$	0	3
$d_{2}$	3	0
(b) Quasi‐complete separation		
$d_{1}$	0	3
$d_{2}$	3	3
(c) Overlap		
$d_{1}$	3	3
$d_{2}$	3	3

In light of the heterogeneity requirement incorporated into the CRML approach, the dose‐finding algorithm is structured accordingly. Stage 1 consists of an initial phase before the implementation of the model in Stage 2. The corresponding flowchart is presented in Supporting Information 7. The target dose is identified as the dose with the estimated probability closest to the target toxicity probability $\phi_{T}$, given by $d^{T}={\text{argmin}}_{j\in \{1,...,J\}}|\psi (x^{T},\hat{\beta })-\phi_{T}|$. While the target toxicity level is typically specified by clinicians, an alternative approach involves referencing response rates from related treatments, which falls outside the scope of the present work [36].

1. *Initial Stage (Stage 1)*:
  • Administer the lowest dose level, $d_{1}$, to the first cohort of patients
  • Evaluate whether the heterogeneity requirement is met based on the observed outcomes. If the criterion is satisfied, proceed to Stage 2
  • If the criterion is not met, determine the next dose based on observed outcomes in the current cohort:
    – 0 DLTs
      * If the current dose level is not the highest (d<5), escalate to the next higher dose.
      * If at the highest dose (d=5), remain at the same dose level
    – 1 DLT:
      * Stay at the same dose level
    – ≥2 DLTs:
      * If at the lowest dose level (d=1):
        · Apply the stopping rule ($\mathrm{Pr}(p_{T}>0.3)\geq 0.95$) if at least two DLTs occur at $d_{1}$, using all accumulated data to assess early stopping
        · If not, remain at the same dose level
      * If not at the lowest dose, de‐escalate to the next lower dose level
2. *Modeling Stage (Stage 2)*:
  • Utilize Maximum Likelihood Estimation (MLE) with the selected model (e.g., the two‐parameter probit model) to iteratively adjust dose administration, optimizing parameter estimates for the probability of toxicity
  • Identify the target dose and determine the subsequent dose for administration, adhering to the no‐skipping rule
  • Conclude the trial upon reaching the pre‐defined sample size, with the recommended dose $d^{T}$ determined based on the accumulated data

For both the joint method and the probit method, there is no requirement on biomarker outcomes in Stage 1. This implies that the consistent structure in the initial stage facilitates comparability across different modeling approaches.

### 2.3 Notation

While changes from baseline are commonly used to evaluate biomarkers in clinical practice, absolute measures, such as fragment length, variant allele frequency (VAF), and specific genomic alterations, are also frequently assessed. It has been shown that certain biomarker levels depend strongly on the cancer type. For simplicity, we denote the repeatedly measured biomarker as $Yc={\left(Y_{c1},Y_{c2},\ldots ,Y_{ct}\right)}^{T}$, representing measurements taken at time points 1 through t within a treatment cycle (e.g., week 1 to week t).

We assume a sequence of linearly decreasing mean biomarker values across dose levels, denoted as $\mu_{c1}>\mu_{c2}>\ldots >\mu_{ct}$, while maintaining a constant variance $\sigma_{c}$. This trend reflects the underlying assumption that lower biomarker levels are associated with an increased risk of adverse effects or toxicity, as exemplified by the relationship between declining platelet counts and treatment‐related toxicity [41]. To capture the temporal relationship between the variances of consecutive biomarker measurements, we assume that the measurements taken closer in time are more strongly correlated in their predictive value. This temporal dependency is modeled using a first‐order autoregressive process, AR(1), characterized by a variance–covariance matrix $\sum_{c}$. This assumption allows for simplicity in the number of parameters. The correlation between biomarker measurements decays exponentially as the time gap between them increases, reflecting the sustained effects of treatment over time. For two time points, t and t+k, the correlation is given by $\rho_{c}^{k}$, where $\rho_{c}$ ($0<\rho_{c}<1$) represents the autoregressive coefficient. Equation (1) illustrates $\sum_{c}$ using t=8 as an example.

(1) $$\sum_{c}=\left(\begin{array}{cccccccc} \sigma_{c}^{2} & \rho_{c}\sigma_{c}^{2} & \rho_{c}^{2}\sigma_{c}^{2} & \rho_{c}^{3}\sigma_{c}^{2} & \rho_{c}^{4}\sigma_{c}^{2} & \rho_{c}^{5}\sigma_{c}^{2} & \rho_{c}^{6}\sigma_{c}^{2} & \rho_{c}^{7}\sigma_{c}^{2}\\ \rho_{c}\sigma_{c}^{2} & \sigma_{c}^{2} & \rho_{c}\sigma_{c}^{2} & \rho_{c}^{2}\sigma_{c}^{2} & \rho_{c}^{3}\sigma_{c}^{2} & \rho_{c}^{4}\sigma_{c}^{2} & \rho_{c}^{5}\sigma_{c}^{2} & \rho_{c}^{6}\sigma_{c}^{2}\\ \rho_{c}^{2}\sigma_{c}^{2} & \rho_{c}\sigma_{c}^{2} & \sigma_{c}^{2} & \rho_{c}\sigma_{c}^{2} & \rho_{c}^{2}\sigma_{c}^{2} & \rho_{c}^{3}\sigma_{c}^{2} & \rho_{c}^{4}\sigma_{c}^{2} & \rho_{c}^{5}\sigma_{c}^{2}\\ \rho_{c}^{3}\sigma_{c}^{2} & \rho_{c}^{2}\sigma_{c}^{2} & \rho_{c}\sigma_{c}^{2} & \sigma_{c}^{2} & \rho_{c}\sigma_{c}^{2} & \rho_{c}^{2}\sigma_{c}^{2} & \rho_{c}^{3}\sigma_{c}^{2} & \rho_{c}^{4}\sigma_{c}^{2}\\ \rho_{c}^{4}\sigma_{c}^{2} & \rho_{c}^{3}\sigma_{c}^{2} & \rho_{c}^{2}\sigma_{c}^{2} & \rho_{c}\sigma_{c}^{2} & \sigma_{c}^{2} & \rho_{c}\sigma_{c}^{2} & \rho_{c}^{2}\sigma_{c}^{2} & \rho_{c}^{3}\sigma_{c}^{2}\\ \rho_{c}^{5}\sigma_{c}^{2} & \rho_{c}^{4}\sigma_{c}^{2} & \rho_{c}^{3}\sigma_{c}^{2} & \rho_{c}^{2}\sigma_{c}^{2} & \rho_{c}\sigma_{c}^{2} & \sigma_{c}^{2} & \rho_{c}\sigma_{c}^{2} & \rho_{c}^{2}\sigma_{c}^{2}\\ \rho_{c}^{6}\sigma_{c}^{2} & \rho_{c}^{5}\sigma_{c}^{2} & \rho_{c}^{4}\sigma_{c}^{2} & \rho_{c}^{3}\sigma_{c}^{2} & \rho_{c}^{2}\sigma_{c}^{2} & \rho_{c}\sigma_{c}^{2} & \sigma_{c}^{2} & \rho_{c}\sigma_{c}^{2}\\ \rho_{c}^{7}\sigma_{c}^{2} & \rho_{c}^{6}\sigma_{c}^{2} & \rho_{c}^{5}\sigma_{c}^{2} & \rho_{c}^{4}\sigma_{c}^{2} & \rho_{c}^{3}\sigma_{c}^{2} & \rho_{c}^{2}\sigma_{c}^{2} & \rho_{c}\sigma_{c}^{2} & \sigma_{c}^{2} \end{array}\right).$$

### 2.4 Framework of Factorization

For clarity, we demonstrate the framework using a bivariate example involving one binary variable $Y_{b}$ and one continuous variable $Y_{c}$. The key idea is to factorize the joint distribution and fit a univariate model to each component. We consider the factorization of a marginal model for continuous outcomes and a conditional model for the binary outcome [42]. This choice aligns with our context, where biomarker values can be assessed earlier than the binary toxicity outcomes. That is, the joint model of $Y_{b}$ and $Y_{c}$ is factorized in the form $f_{Yb,Yc}(y_{b},y_{c})=f_{Yc}(y_{c})f_{Y_{b}|Y_{c}}(y_{b}|y_{c})$. This factorization can be motivated by assuming that the binary outcome $Y_{b}$ is a dichotomization of an underlying latent continuous variable $Y_{b}^{*}$.

(2) $$Y_{b}=\left\{\begin{array}{ll} 0,\; & \text{if}\;Y_{b}^{*}\leq 0\\ 1, & \text{if}\;Y_{b}^{*}>0 \end{array},\right.$$

where $Y_{b}^{*}∼𝒩(\mu_{b}^{*},1)$. The underlying variable $Y_{b}^{*}$ is assumed to follow a standard normal distribution 𝒩(0,1), as this avoids any loss of generality compared to using a normal distribution with an arbitrary mean and standard deviation.

We employ a probit regression model for the conditional distribution of the binary outcome $Y_{b}$. The logistic model is often favored for its intuitive interpretation of coefficients in terms of odds ratios. However, we choose the probit link for its mathematical convenience, particularly the availability of closed‐form solutions in certain multivariate model calculations. Despite these differences, the probit and logistic regression models generally yield similar predictions [43].

The joint method models the joint probability by using a bivariate linear regression framework, structured as follows:

(3) $$\left(\begin{array}{c} Y_{c}\\ Y_{b}^{*} \end{array}\right)∼𝒩\left(\left(\begin{array}{c} \mu_{c}\\ \mu_{b}^{*} \end{array}\right),\left(\begin{array}{cc} \sigma_{c}^{2} & \rho_{b}\sigma_{c}\\ \rho_{b}\sigma_{c} & 1 \end{array}\right)\right),$$

where the correlation between two outcomes is represented as $\rho_{b}$. The joint bivariate normal distribution of $Y_{b}^{*}$ and $Y_{c}$ can be expressed as the product of the marginal distribution of $Y_{c}∼𝒩(\mu_{c},\sigma_{c}^{2})$ and the conditional distribution of $Y_{b}^{*}$ given $Y_{c}$:

(4) $$Y_{b}^{*}|Y_{c}=y_{c}∼𝒩\left(\mu_{b}^{*}+\frac{\rho_{b}}{\sigma_{c}}(Y_{c}-\mu_{c}),\;(1-{\rho_{b}}^{2})\right),$$

where $\mu_{b}^{*}=\beta_{b0}^{*}+\beta_{b1}^{*}\cdot x$ and $\mu_{c}=\beta_{c0}+\beta_{c1}\cdot x$, representing the dose‐response relationships for binary and continuous outcomes, respectively. The conditional variance needs to be standardized to ensure identifiability in our bivariate probit model. This implies

(5) $$E(Y_{b}|Y_{c}=y_{c})=P(Y_{b}^{*}>0|Y_{c}=y_{c})=\Phi \left(\frac{\mu_{b}^{*}+\frac{\rho_{b}}{\sigma_{c}}(Y_{c}-\mu_{c})}{\sqrt{\left(1-{\rho_{b}}^{2}\right)}}\right).$$

We reparameterize the model as follows:

(6) $$E(Y_{b}|Y_{c}=y_{c})=P(Y_{b}^{*}>0|Y_{c}=y_{c})=\Phi \left(\mu_{b}+\tau (Y_{c}-\mu_{c})\right),$$

where $\mu_{b}=\beta_{b0}+\beta_{b1}\cdot x$ (with $\beta_{b0}=\frac{\beta_{b0}^{*}}{\sqrt{\left(1-{\rho_{b}}^{2}\right)}}$ and $\beta_{b1}=\frac{\beta_{b1}^{*}}{\sqrt{\left(1-{\rho_{b}}^{2}\right)}}$), $\mu_{c}=\beta_{c0}+\beta_{c1}\cdot x$ and $\tau =\frac{\rho_{b}}{\sigma_{c}\sqrt{1-{\rho_{b}}^{2}}}$. This reparameterization addresses identifiability concerns by ensuring that the model parameters are uniquely determined [44].

In this context, $\beta_{b}=(\beta_{b0},\;\beta_{b1})$ represents the effects of the covariates on the probit of $\mu_{b}$, conditional on $Y_{c}$. Consequently, the corresponding marginal effects, denoted as $\beta_{b}^{*}=(\beta_{b0}^{*},\;\beta_{b1}^{*})$, can be derived by integrating over $Y_{c}$ and rescaling the conditional effects accordingly. Specifically, this rescaling yields

(7) $$\beta_{b0}^{*}=\beta_{b0}/\sqrt{\left(1+\sigma_{c}^{2}\tau^{2}\right)}\;\text{and}\;\beta_{b1}^{*}=\beta_{b1}/\sqrt{\left(1+\sigma_{c}^{2}\tau^{2}\right)}.$$

We consider the Joint9d method, which incorporates eight values of biomarkers, and the Joint2d method, which includes a single biomarker value. The Probit method, relying solely on binary toxicity outcomes, serves as the baseline for comparison. Further details about derivation of the Joint9d method are provided in Supporting Information 9. MLE for all methods is performed using the *bbmle* [45] package in R [46], employing the *nlminb* optimization algorithm.

## 3 Modeling Challenges in Dose‐Finding Studies

Parameter estimation within the frequentist framework can be challenging, especially when data are limited, as is frequently the case in the early stages of dose‐finding studies. In this section, we highlight key issues that arise during model fitting. To illustrate these challenges, we present two hypothetical trial realizations in phase I, each demonstrating a common estimation problem encountered in dose‐finding studies.

For illustration, we consider two hypothetical trials in Section 3.1 and Section 3.2, with J=5 investigated dose levels and a corresponding dose‐toxicity skeleton $p_{0}=(0.25,0.35,0.45,0.55,0.65)$. The dose labels x are derived from the skeleton using the model, as described in detail in Section 5. The total sample size is fixed at N=60 and the trail starts at the lowest dose $d_{1}$. The target response rate is set to $\phi_{T}=0.3$. A total of 20 cohorts are assumed to be enrolled sequentially over the course of the trial. The trial assumes an initial stage satisfying the heterogeneity requirement, as in the original CRML proposal [29].

### 3.1 Example Trial 1: Anchoring at a Lower Dose With a Step Function

In the example trial shown in Figure 1, the initial stage involves two cohorts to satisfy the heterogeneity requirement: no DLTs are observed in the first cohort, while one DLT is recorded in the second. This results in a quasi‐separation data configuration, as summarized in Table 2. Consequently, the two‐parameter probit model repeatedly selects $d_{2}$ from the second cohort onward, persisting until the trial conclusion. This outcome arises because the model produces a step function, as depicted in the right panel of Figure 1. The estimated probability of toxicity for $d_{1}$ and $d_{2}$ remains below 0.3, while the probabilities for $d_{3}$ through $d_{5}$ are estimated to approach 1. As a result, doses $d_{3}$ through $d_{5}$ are never explored during the trial.

**FIGURE 1:**
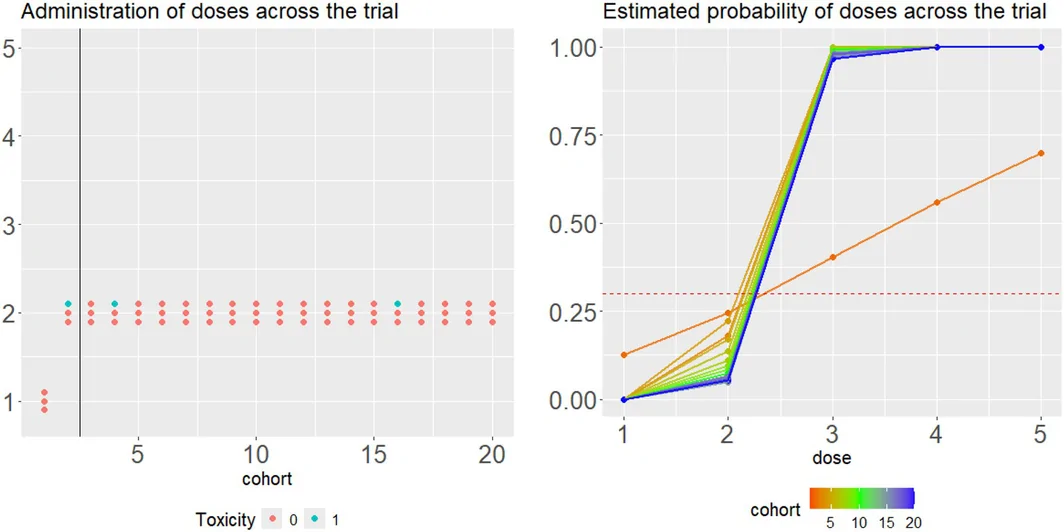
Illustration of a potential anchoring problem in a hypothetical trial using the Probit method. Stage 1 includes two cohorts, followed by Stage 2, which applies the two‐parameter Probit model.

We refer to this phenomenon as the *anchoring problem* in dose‐finding studies, where dose escalation becomes stalled as a result of the step function of the estimated probability for investigated doses. Once this issue arises, further dose escalation to higher levels becomes infeasible, as these doses are perceived to be too distant from the target probability $\phi_{T}$. Consequently, higher doses remain unexplored, and subsequent cohorts are unable to advance the trial. Ultimately, this can lead to an underdosing problem, depending on the target dose in a given scenario.

This phenomenon is more broadly known as the *separation problem* in parameter estimation, where the covariate space (i.e., the investigated doses) is effectively divided into two distinct categories by the step function, leading to an infinite value (i.e., complete separation) or an extremely large value (i.e., quasi‐complete separation) for the MLE of the covariate. This issue was also highlighted in the original CRML paper [29], which notes that when higher dose levels are the correct choice, “the CRML tends to spend too much time at lower levels due to slow escalation caused by grouping.” In this context, “grouping” refers to the partitioning or separation of doses by the step function.

Paoletti and Kramar [33] demonstrated that the two‐parameter model fails to identify the correct dose in challenging scenarios where lower doses yield only non‐DLT. This issue is illustrated in greater detail by the example trial shown in Figure 1, which provides a concrete visualization of the data conditions that lead to parameter nonidentification and consequently incorrect dose selection.

The joint modeling methods introduced in Section 2.4 have the potential to mitigate the anchoring problem under identical initial‐stage conditions. With the same number of patient cohorts in the initial stage, the inclusion of biomarker information can reduce the risk of outcome‐driven separation in the covariate space when fitting joint models. For illustrative purposes, we first consider an example in which the biomarker is completely irrelevant (i.e., $\rho_{b}=0$). Additional details on data generation under various assumptions are provided in Section 5; we omit them here, as they are not central to the current discussion.

This example is visualized in Figure 2, which presents the same hypothetical trial under the two‐stage design described in Section 2.2, now implementing the Joint2d and Joint9d methods in Stage 2. All other aspects of the hypothetical trial are comparable to those in Figure 1. With the same initial stage consisting of two patient cohorts to satisfy the heterogeneity requirement, dose administration under the Joint2d method mirrors that of the Probit method in Figure 1, as the step function in the estimated probability persists. However, the Joint9d method promotes dose escalation starting from the 3rd cohort, immediately after the initial stage, enabling a more efficient exploration of higher dose levels. As more cohorts are enrolled, the estimated probabilities are continuously updated, ultimately leading to the selection of the highest dose, $d_{5}$, as the recommended dose.

**FIGURE 2:**
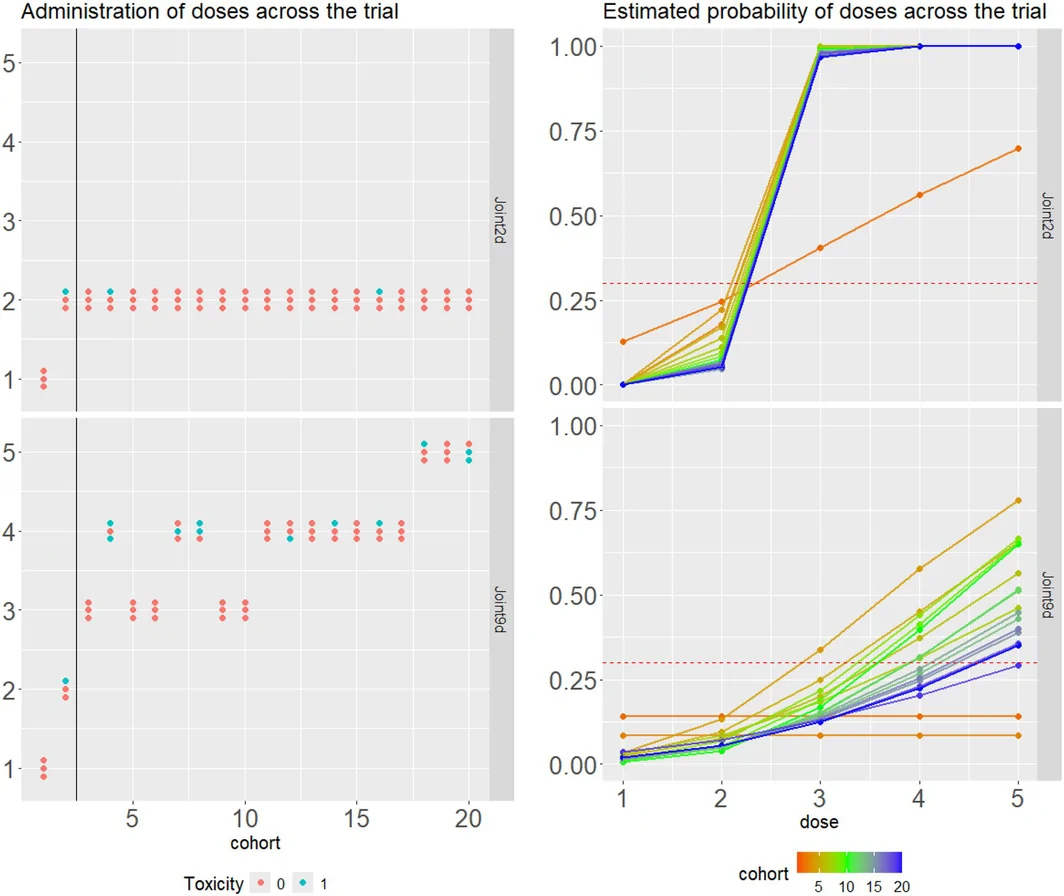
Illustration of the potential anchoring problem in a hypothetical trial using joint modeling approaches. Stage 1 includes two cohorts, and Stage 2 implements the Joint2d and Joint9d methods based on the two‐parameter Probit model.

This trial example illustrates the estimation problem encountered with the likelihood‐based approach due to the limited dataset typical of the early stages of dose‐finding studies. As discussed in Section 2.2, considering the mutually exclusive data configurations, complete separation occurs when the predictor variables perfectly predict the outcome, leading to infinite MLEs. Quasi‐complete separation arises when the outcome is perfectly predicted for a subset of the data, also resulting in problematic MLEs. In contrast, the overlap condition, where the predictor variables do not perfectly predict the outcome, ensures the existence of finite MLEs. The heterogeneity requirement, defined as the presence of both DLTs and non‐DLTs, is motivated to avoid separation issues. However, it is slightly weaker than the overlap condition, as noted by O'Quigley [32].

In dose‐finding studies, limited datasets often face separation or quasi‐separation issues, even when the heterogeneity requirement is satisfied. For example, lower doses may result in only non‐DLTs, while DLTs are observed only at the highest doses. This data configuration can lead to inappropriate estimates of the slope parameter and, consequently, a step function in the estimated probabilities, which can hinder dose‐escalation decisions. Figure 1 and Figure 2 illustrate these challenges using the Probit and Joint2d methods. Incorporation of higher‐dimensional biomarker values (e.g., Joint9d in Figure 2) offers a practical solution in this specific example trial, as higher‐dimensional biomarker values, although noisy, help prevent the covariate space (including dose and continuous outcomes, as defined in the conditional probit model in Equation 11) from becoming separated by the outcomes.

### 3.2 Example Trial 2: Equal Estimated Probability Problem

Another challenge in parameter estimation arises when the dose‐toxicity curve appears flat rather than monotonic after observing outcomes. This can happen if the empirical toxicity probabilities (i.e., observed frequencies) are similar across doses or if the data inadvertently suggest a decreasing dose‐toxicity trend.

Figure 3 illustrates a hypothetical trial following the two‐stage design described in Section 2.2, where Stage 1 consists of a single cohort. As noted in Section 2, the non‐negativity constraint on $\exp(\beta_{b1})$ results in estimated toxicity probabilities that remain similar across all dose levels throughout the trial. This constraint tends to arise in extreme scenarios where the doses have nearly indistinguishable toxicity profiles, either uniformly very safe or uniformly overly toxic. In such cases, the impact on target dose selection is generally minimal since the recommended dose may still be appropriate despite the lack of precision in toxicity estimates.

**FIGURE 3:**
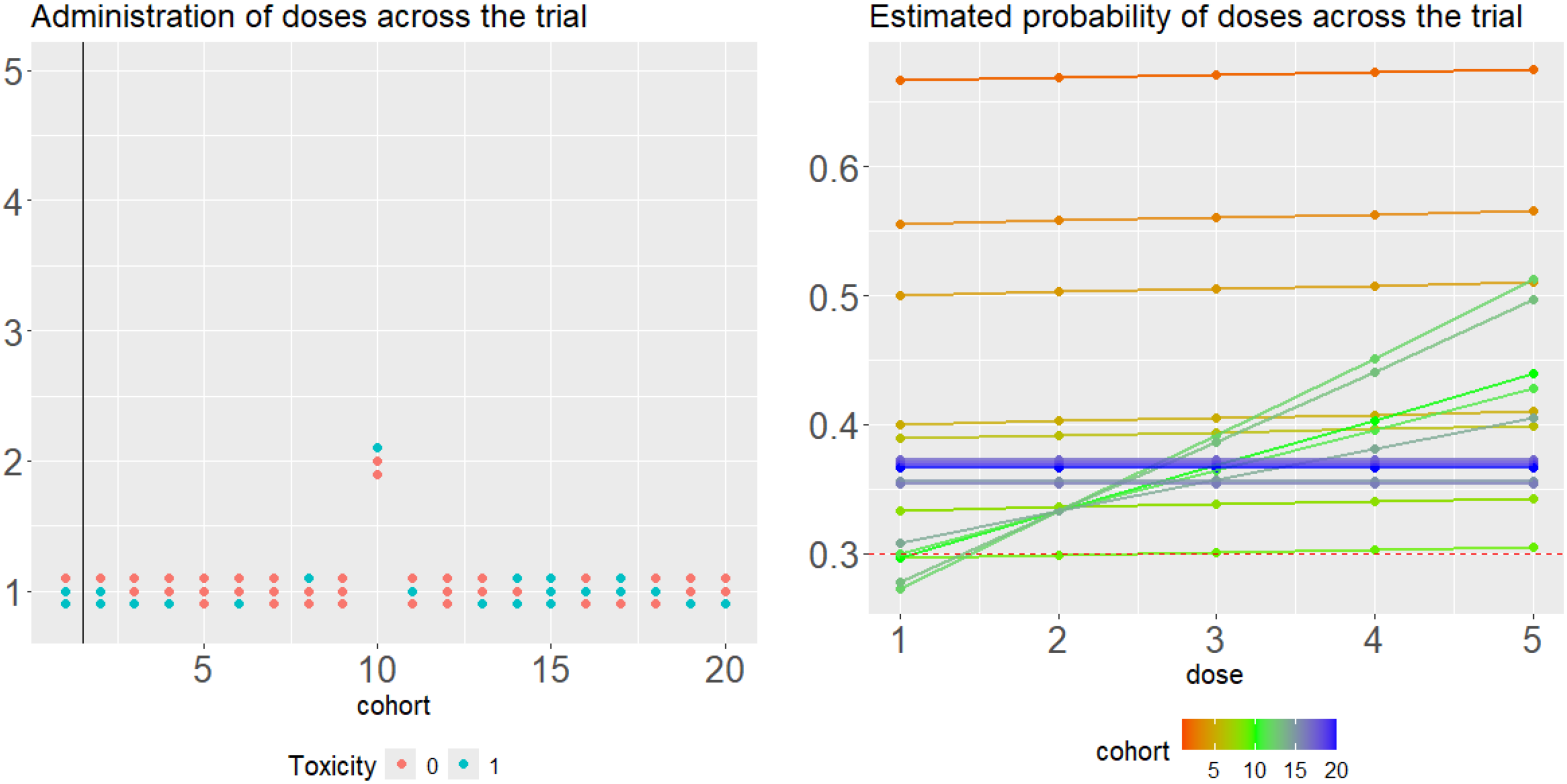
Illustration of the potential issue of equal estimated probabilities in a hypothetical trial using the Probit method. Stage 1 includes one cohort, followed by Stage 2, which applies the two‐parameter Probit model.

Similarly, Figure 4 demonstrates that adding irrelevant biomarker variable (i.e., $\rho_{b}=0$) does not enable the Joint2d and Joint9d method to differentiate between doses when the frequencies of the observed toxicity outcomes are similar. The dose–toxicity curve eventually flattens again in the end.

**FIGURE 4:**
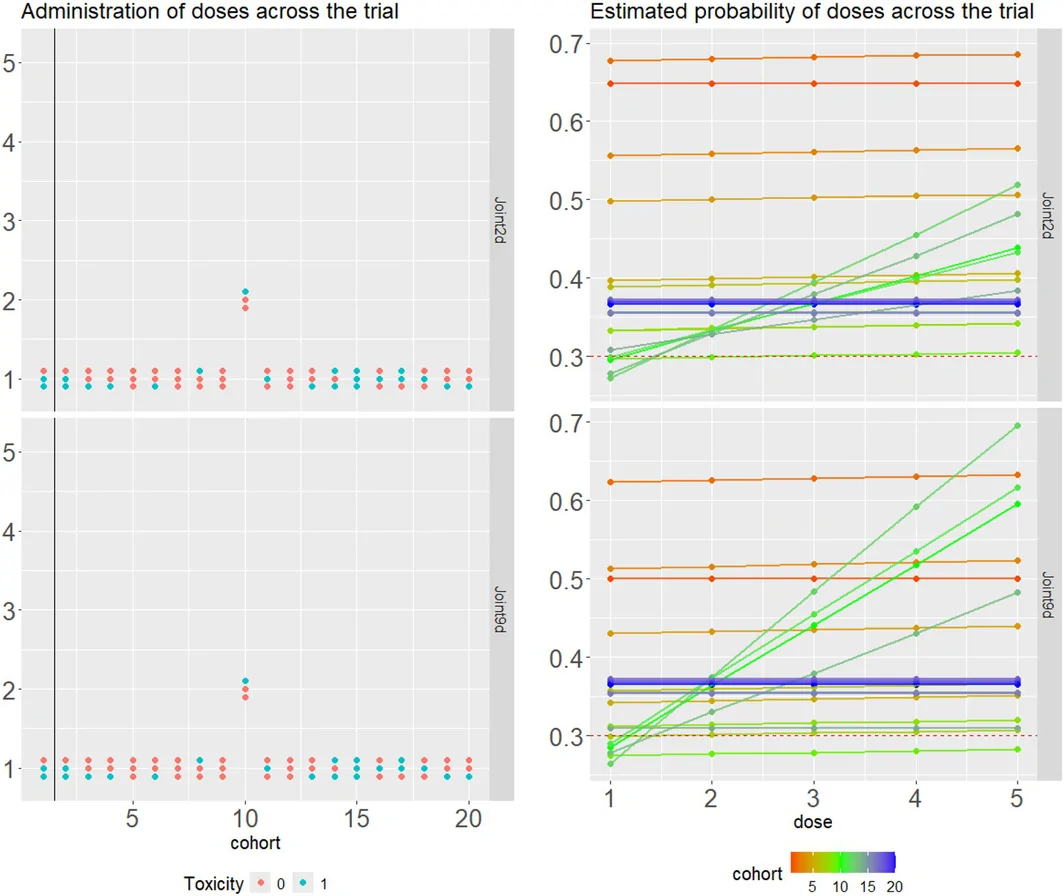
Illustration of the potential equal estimated problem in a hypothetical trial using the joint methods. Stage 1 includes one cohort and Stage 2 applies the Joint2d and Joint9d methods based on the two‐parameter probit model.

This example highlights the issue of equal estimated probabilities across doses, yet different methods ultimately manage to capture information from data and lead to the desired dose recommendation by the end of the trial. All methods attempt to depict the monotonic curve in the middle of the trial after approximately 10 cohorts. However, in the end, it reverts to a flat line, overwhelmed by the information transformed from the binary outcomes.

Overall, there are two primary types of estimation challenges in dose‐finding studies, where the final dose‐toxicity curve is either estimated as a step function or a flat line. A key consideration is whether the joint methods with additional biomarker values can consistently resolve estimation challenges across different scenarios and how its performance is affected when biomarker values contain meaningful information rather than just noise. Moreover, while the original proposal claiming that overall fit was not the primary objective of phase I studies [28], it is crucial to emphasize that these estimation problems, especially the separation problem, should ideally be avoided from the outset to ensure a fair comparison across different methods and produce clinically meaningful results.

We intend to address these two issues in this section in a different way. It is essential to avoid step functions, as they can hinder the dose‐escalation process. While a flat curve is unexpected, it can still provide valuable insights based on empirical toxicity outcomes. Thus, we focus on remedies for the anchoring problem, while still illustrating the proportion of equal probability estimation cases in comprehensive simulations.

## 4 Remedies for Modeling Challenges

To address the parameter estimation challenges with limited data in Section 3.1, two general approaches can be considered. One solution is to increase the dataset size during the initial stage, which introduces greater complexity but may ensure the existence of the MLE in the modeling stage. Alternatively, the model may be simplified, such as by adopting a one‐parameter probit model or a empiric model. For binary outcome analysis with small sample sizes, established techniques such as Firth's logistic regression [47] and Jeffreys' prior [48] have been developed.

In this study, we focus specifically on addressing the estimation problem in the Probit method and its corresponding joint methods within the frequentist framework. In addition to increasing the complexity of the initial stage (Section 4.1) and employing a one‐parameter model (Section 4.2), we explore the calibration of the dose labels (Section 4.3).

### 4.1 Initial Stage

It is highly unlikely that the initial stage in dose‐finding designs will satisfy the overlap requirement, particularly in cases where lower doses are safe and consistently result in non‐DLTs. To address this, we increase the complexity of the initial stage by requiring more occurrences. For example, a rule‐based “2+2” initial stage, which requires at least two DLTs and two non‐DLTs, has been used by Altzerinakou and Paoletti [49]. We refer to this more complex initial stage as Initial2, while the simpler heterogeneity requirement [29] is referred to as Initial1. Similarly, more complex stages such as Initial3 (at least three DLTs and three non‐DLTs) and Initial4 (at least four DLTs and four non‐DLTs) can also be implemented in Stage 1.

### 4.2 One‐Parameter Model

The one‐parameter models, including the empiric model and the one‐parameter probit model with a fixed intercept (see Table 1) are commonly used in the CRM literature. The empiric model is widely recognized for its superior performance compared to the two‐parameter logistic model (and similarly, the two‐parameter probit model) [33]. A simulation study by Chevret [50] illustrated that the performance of CRM via power model could be improved by the use of the one‐parameter logistic model, although results were rather sensitive to the choice of the fixed parameter $a_{0}$.

However, one often‐overlooked aspect in previous investigations is that the intercept $a_{0}$ implicitly encodes information about a *reference dose* $x^{*}=0$. The probability of toxicity at this reference dose is determined at $\pi (x^{*})=\psi (x^{*})=\psi (a_{0})$, independent of the parameter estimation for $\beta_{b1}$. As a result, the dose‐toxicity curve and the estimation of toxicity at investigated doses can be significantly influenced by the reference dose $x^{*}$, equally, the intercept $a_{0}$. When $x^{*}$ falls within the range of dose labels, the fixed probability $\pi (x^{*})$, determined by $a_{0}$, acts as a dividing point in the dose‐response relationship. For example, if $a_{0}=0$, the skeleton $p_{0}=(0.25,0.35,0.45,0.55,0.65)$ corresponds to the transformed dose labels x=(−0.67,−0.39,−0.10,0.15,0.39), with the division occurring between $d_{3}$ and $d_{4}$. Specifically, $\hat{\pi }(x_{j})<0.5$ holds for all doses below $d_{4}$. More generally, the dichotomization of dose labels and the estimated probability $\pi (x^{*})$ depends on the chosen model or the value of $a_{0}$, as shown in Equation (8).

(8) $$\hat{\pi }(x_{j})\left\{\begin{array}{ll} <\pi (x^{*})\; & \text{if}\;x_{j}<x^{*}\\ >\pi (x^{*})\; & \text{if}\;x_{j}>x^{*} \end{array}\right.,\text{where}\;(x^{*}=0).$$

Figure S13 in Supplement Information 8 demonstrated the one‐parameter probit model under different choices of $a_{0}$, each leading to distinct sets of transformed dose labels (see Table 1). Among the choices shown, $a_{0}=0$ is the only one that results in the dichotomization of dose levels. In the case $a_{0}=0.5$, the transformed dose labels, x=(−1.17,−0.89,−0.63,−0.37,−0.11), are not dichotomized by $x^{*}=0$. Instead, the reference dose $x^{*}$ acts as an upper limit, with $\pi (x^{*})=0.69$. In such cases, the model may lead to underestimation if any dose $x_{j}$ satisfies $\pi (x_{j})>0.69$.

Ultimately, we conclude that a sufficiently large value of $a_{0}$ is essential for the one‐parameter probit model to prevent the reference dose $\left(x^{*},\pi (x^{*})\right)$ from dichotomizing dose levels or imposing inappropriate upper limits on the estimated probabilities of response. However, selecting an excessively large value for $a_{0}$ is not always advisable, as it may result in excessively large arguments in the normal cumulative distribution function of the probit model or logistic model, potentially causing the estimated probabilities for all doses to converge to one [51]. This issue reminds us that choosing an overly large $a_{0}$ does not necessarily resolve convergence issues and may, in fact, exacerbate them. Chevret [50] investigated the choice of $a_{0}$ in the one‐parameter logistic model within a Bayesian framework and recommended performing simulation studies to assess sensitivity before finalizing its value.

### 4.3 Calibration for Dose Labels

Paoletti and Kramar [33] evaluated the impact of the coding system, which codes value for dose level, on the operating characteristics of the empiric model. Their findings indicated that the choice of coding system can substantially influence the performance of the CRM when using the empirical model.

Lee and Cheung [52] proposed a systematic approach for calibrating the initial dose‐toxicity skeleton in the Bayesian CRM framework. While their method was developed within a Bayesian framework, the underlying principle of systematically optimizing the spacing between dose levels provides conceptual motivation for dose label calibration in likelihood‐based methods, though the specific optimization criterion would require adaptation.

A systematic calibration of the implemented dose levels, incorporating both the skeleton and the original model (i.e., initial guess of parameters), may further improve the operating characteristics of the Probit method, as well as the joint methods. Future comparisons of these methods could also be expanded to include dose label calibration.

## 5 Simulation

To investigate the contribution of additional biomarker values in dose‐finding studies, we evaluate the performance of the joint methods, specifically, the Joint9d and Joint2d approaches, through a comprehensive simulation study, using the Probit method as a baseline for comparison. The data generation process and simulation settings are detailed in Section 5.1 and Section 5.2. The operating characteristics used to assess the performance of the methods are introduced in Section 5.3. Furthermore, to evaluate the effectiveness of the remedies proposed in Section 4, we first apply all remedies within the same hypothetical trial introduced in Section 3.1 and present the corresponding simulation results across various scenarios.

### 5.1 Data Generation

We consider a study with five doses, $d_{1}<d_{2}<d_{3}<d_{4}<d_{5}$, corresponding to dose labels $x_{1}<x_{2}<x_{3}<x_{4}<x_{5}$. The transformation of these dose labels is outlined in Table 1 and is applied uniformly to both the Probit and joint methods. The trial design includes an eight‐week evaluation window for binary toxicity outcomes. Biomarker measurements are taken at week 1 (one week after recruitment) and at the end of each subsequent week, yielding eight measurements in total (i.e., week 1 to week 8). The final measurement at week 8 is collected concurrently with the binary outcomes at the end of the evaluation window. Dose administration decisions for the subsequent cohort are made once the toxicity data becomes available.

The data generation process uses complete information about patient profiles as the optimal benchmark approach, which was originated in O'Quigley et al. [53] and extended in Mozgunov et.al. [54]. Assuming that the outcomes of each patient can be observed at all dose levels, this allows minimizing simulation error and providing a more accurate comparison between methods.

Specifically, we generate correlated random variables from a standard multivariate normal distribution to represent patients' profiles, which serve as the basis for generating complete outcomes across all doses. For each patient i administered dose $d_{j}$, a 9‐dimensional random vector is drawn from a multivariate normal distribution, as specified in Equation (9). This vector incorporates correlation through the variance–covariance matrix, ∑. The patients' profiles are obtained by transforming these random variables using the standard normal probability density function. Subsequently, complete responses are derived via quantile transformations using the corresponding cumulative distribution functions; that is, $Y_{ci,t}=\Phi^{-1}(u_{ci,t})$ for the continuous outcomes. Similarly for binary outcomes, $Y_{bi}=1-F^{-1}(u_{bi})$, where F is CDF of Bernoulli distribution. In this context, the parameter $\rho_{c}$ and $\rho_{b}$ correspond to the relationship in the R.V. in Equation (9). Specifically, $\rho_{c}$ represents the autoregressive coefficient under the AR(1) assumption, while $\rho_{b}$ quantifies the correlation between $x_{bi}$ and $x_{ci,8}$ corresponding to the lastly evaluated biomarker value. To demonstrate how we generate complete patient information, we present an example involving two hypothetical patients with $Y_{ci,8}$ and $Y_{bi}$ for patient i=1 and patient i=2.

(9) $$\left(\begin{array}{c} u_{ci,1}\\ u_{ci,2}\\ u_{ci,3}\\ u_{ci,4}\\ u_{ci,5}\\ u_{ci,6}\\ u_{ci,7}\\ u_{ci,8}\\ u_{bi} \end{array}\right)∼𝒩\left(\left(\begin{array}{c} 0\\ 0\\ 0\\ 0\\ 0\\ 0\\ 0\\ 0\\ 0 \end{array}\right),\left(\begin{array}{ccccccccc} 1 & \rho_{c} & \rho_{c}^{2} & \rho_{c}^{3} & \rho_{c}^{4} & \rho_{c}^{5} & \rho_{c}^{6} & \rho_{c}^{7} & \rho_{b}^{8}\\ \rho_{c} & 1 & \rho_{c} & \rho_{c}^{2} & \rho_{c}^{3} & \rho_{c}^{4} & \rho_{c}^{5} & \rho_{c}^{6} & \rho_{b}^{7}\\ \rho_{c}^{2} & \rho_{c} & 1 & \rho_{c} & \rho_{c}^{2} & \rho_{c}^{3} & \rho_{c}^{4} & \rho_{c}^{5} & \rho_{b}^{6}\\ \rho_{c}^{3} & \rho_{c}^{2} & \rho_{c} & 1 & \rho_{c} & \rho_{c}^{2} & \rho_{c}^{3} & \rho_{c}^{4} & \rho_{b}^{5}\\ \rho_{c}^{4} & \rho_{c}^{3} & \rho_{c}^{2} & \rho_{c} & 1 & \rho_{c} & \rho_{c}^{2} & \rho_{c}^{3} & \rho_{b}^{4}\\ \rho_{c}^{5} & \rho_{c}^{4} & \rho_{c}^{3} & \rho_{c}^{2} & \rho_{c} & 1 & \rho_{c} & \rho_{c}^{2} & \rho_{b}^{3}\\ \rho_{c}^{6} & \rho_{c}^{5} & \rho_{c}^{4} & \rho_{c}^{3} & \rho_{c}^{2} & \rho_{c} & 1 & \rho_{c} & \rho_{b}^{2}\\ \rho_{c}^{7} & \rho_{c}^{6} & \rho_{c}^{5} & \rho_{c}^{4} & \rho_{c}^{3} & \rho_{c}^{2} & \rho_{c} & 1 & \rho_{b}\\ \rho_{b}^{8} & \rho_{b}^{7} & \rho_{b}^{6} & \rho_{b}^{5} & \rho_{b}^{4} & \rho_{b}^{3} & \rho_{b}^{2} & \rho_{b} & 1 \end{array}\right)\right)$$

*Example*: Consider an extreme case where $\rho_{b}$ indicates a perfect relationship between $u_{ci,8}$ and the toxicity outcomes $u_{bi}$. The bivariate normal vector, with mean $\mu_{i}=(0,0)$ and covariance matrix $\sum =\left(\begin{array}{ll} 1 & \rho_{b}\\ \rho_{b} & 1 \end{array}\right)$ is initially generated as $\left(x_{bi},\;x_{ci,8}\right)$. In this example (see Table 3), the first simulated patient i=1 has a toxicity profile of $u_{bi}=\Phi (0.46)=0.68$ and an biomarker profile $u_{ci,8}=\Phi (0.46)=0.68$. These values correspond to the toxicity response $Y_{bi}=1-F^{-1}(u_{bi},\;\pi_{T})$ and biomarker values $Y_{ci,8}=\Phi^{-1}(u_{ci,8},\;\mu_{c8},\;\sigma_{c}=1)$. For illustrative purposes, we assume $\pi_{T}=(0.30,\;0.45,\;0.55,\;0.65,\;0.75)$ and $\mu_{c8}=(10,\;8,\;6,\;4,\;2)$. In the end, the treatment is more toxic for patient i=1 than for patient i=2, being indicated by a higher likelihood of favorable toxic responses ($Y_{b}=1$ at d=5) and generally lower biomarker values (see Table 3). It is important to note that, for illustrative purposes in the data generation process, we assume $\rho_{b}=1$, indicating a perfect correlation between the profiles for binary and continuous outcomes. However, this assumption is unlikely to hold in practical applications.

**TABLE 3:** Example of two patients with complete information generation.

Patient i	($x_{bi},\;x_{ci,8}$)	($u_{bi},\;u_{ci,8}$)	$Y_{bi}$	$Y_{ci,8}$
i=1	(0.46, 0.46)	(0.68, 0.68)	(0,0,0,0,1)	(10.47, 8.47, 6.47, 4.47, 2.47)
i=2	(1.34, 1.34)	(0.91, 0.91)	(0,0,0,0,0)	(11.34, 9.34, 7.34, 5.34, 3.34)

The Probit method exclusively uses binary outcomes, $Y_{b}$, while the Joint2d method, in addition to $Y_{b}$, incorporates a single biomarker measurement, $Y_{c8}$, collected at week 8 concurrently with the binary outcomes. The Joint9d method extends this by utilizing all biomarker measurements from week 1 to week 8, along with $Y_{b}$.

Weekly values of biomarker are modeled as normally distributed $Y_{c}∼𝒩(\mu_{c},\sum_{c})$, where the vector $\mu_{c}$ represents the mean values $\mu_{ct}(x)$ (t∈[1,8]) and $\sum_{c}$ denotes the variance‐covariance matrix of biomarker values. The linear relationship between time t, dose x, and $\mu_{ct}$ is assumed as

(10) $$\mu_{ct}(x)=\beta_{c0}+\beta_{c1}\cdot x+a_{t}\cdot t.$$

In Equation (10), x represents the candidate dose, and $\beta_{c1}$ captures the relationship between doses and the mean value of biomarker responses. We assume a fixed linear relationship for patient responses in biomarker over time, $a_{t}$, reflecting the treatment's effectiveness in reducing biomarker values on average. A negative value of $a_{t}$ indicates more harm from treatment with time. The variance–covariance matrix of the multivariate distribution, $\sum_{c}$, is modeled under the assumption of autocorrelation as detailed in Section 2.3.

While this study assumes fixed linear trajectories for mean values, within‐subject variance, and intrasubject variance, alternative configurations could be explored in future research. This work represents an initial investigation, highlighting the potential challenges and advantages of integrating biomarker data into dose‐finding decisions within the framework of joint models.

### 5.2 Simulation Scenarios and Parameter Choices

We analyze the simulation results under varying correlations of $\rho_{b}$ and $\rho_{c}$ (see Table 4a) across different scenarios of dose‐response relationship presented in Table 4b. In this initial investigation, the variance of biomarker values is fixed at $\sigma_{c}=1$.

**TABLE 4:** Simulation scenarios and corresponding design parameters.

(a) Summary of design parameters		
Notation	Interpretation	Value
s	Number of simulations	1000
N	Total number of patients	60
n	Number of cohorts	20
m	Cohort size	3
J	Number of dose levels	5
$\rho_{b}$	Correlation between $x_{b}$ and $x_{c,8}$	(0,0.4,0.8)
$\rho_{c}$	Correlation between adjacent biomarker	0.4
$\sigma_{c}$	Variance of biomarker values	1
$\beta_{c0}$	Intercept for $\mu_{ct}$ at baseline	20
$\beta_{c1}$	Impact of dose on $\mu_{ct}$	−2
$a_{t}$	Impact of time on $\mu_{ct}$	−1

	(b) Scenarios with the targeted dose
	$\pi_{T}(x_{j})$
$S_{1}$	(**0.30**, 0.45, 0.55, 0.65, 0.75)
$S_{2}$	(0.15 **0.30** 0.45 0.55 0.65)
$S_{3}$	(0.10 0.15 **0.30** 0.45 0.55)
$S_{4}$	(0.05 0.10 0.15 **0.30** 0.45)
$S_{5}$	(0.05 0.08 0.10 0.15 **0.30**)

To ensure a positive conditional variance in the Joint2d and Joint9d methods (Section 2.4) and maintain a positive‐definite covariance matrix for data generation, parameter selection is restricted. Specifically, a combination of a large $\rho_{b}$ and a small $\rho_{c}$ is not suitable. More details about the constraints on the choices of $\rho_{b}$ and $\rho_{c}$ are provided in Supporting Information 10.

Since our primary interest lies in the impact of the correlation between biomarker and binary toxicity outcomes, we fix $\rho_{c}$ at a moderate value $\rho_{c}=0.4$ and explore simulation results for $\rho_{b}=(0,0.4,0.8)$. While this study focuses on these specific configurations, alternative settings could be explored in future research. Table 4 also summarizes other key parameters used in the study design. The simulated correlation values are intended to reflect a range of clinically plausible settings. Since the true correlation between a continuous biomarker and a composite DLT endpoint is unknown in practice and may vary considerably across agents and patient populations, we evaluate our framework under weak, moderate, and strong correlation assumptions $\rho_{b}=(0,0.4,0.8)$. The $\rho_{b}=0$ scenario captures settings where the biomarker provides no predictive signal and the joint model reduces to the standard approach, while $\rho_{b}=0.8$ reflects settings where the DLT endpoint is dominated by a toxicity type for which the biomarker is a strong predictive marker. Overall, the proposed approach is expected to offer greatest value for agents with well‐characterized, biomarker‐related toxicity mechanisms.

The investigated scenarios are summarized in Table 4b. Scenarios $S_{1}-S_{2}$ are relatively straightforward, as the dose‐toxicity probabilities span a wider range, yielding a steeper effective slope that enables the model to distinguish between dose levels more readily. Scenarios $S_{3}-S_{4}$ represent moderate difficulty with a more gradual progression. Scenario $S_{5}$ is the most challenging: the target lies at the highest dose, while the lower doses exhibit very low toxicity probabilities (0.05, 0.08, 0.10 at $d_{1}-d_{3}$), producing an effectively flat slope across the lower dose range that is likely to trigger the anchoring problem described in Section 3.1.

### 5.3 Operating Characteristics

For performance evaluation, we focus primarily on four metrics. First, we assess (1) *the proportion of correct selections (PCS)*. In addition, to investigate the estimation issues discussed in Section 3, we include two supplementary metrics: (2) *the proportion of separation*, defined as the proportion of cases in which the estimated probabilities satisfy either $|\hat{\pi }(d_{i})-0|\leq 10^{-4}$ or $|\hat{\pi }(d_{i})-1|\leq 10^{-4}$; and (3) *the proportion of equal estimated probabilities*, defined as the proportion of cases in which $|\hat{\pi }(d_{5})-\hat{\pi }(d_{1})|\leq 10^{-4}$. Finally, for safety considerations, we report (4) *the proportion of early stopping* due to excessive toxicity in the initial stage. All results are based on s=1000 simulation replications; see Table 4 for the full set of design parameters. Unless otherwise stated, all operating characteristics are reported to two decimal places, with the exception of the proportion of equal estimated probabilities, which is reported to three decimal places owing to the small magnitude of this quantity.

### 5.4 Impact of Initial Stage

Given access to the same complete information described in Section 3.1, we ensure a fair comparison by examining the trial in Figure 1 with identical setups but varying initial stages. In the example presented in Figure 1, the Initial1 stage comprises two cohorts. In contrast, the Initial3 stage extends beyond the second cohort, continuing until two additional toxicities are observed. Ultimately, the Initial3 stage involves seven cohorts: one toxicity occurs in the second cohort at dose level $d_{2}$, one, occurs in the 4th cohort at $d_{3}$, and the last one occurs in the 7th cohort at $d_{5}$ (see Table 5).

**TABLE 5:** Comparison of different initial stages with the same hypothetical trial shown in Figure 1.

Cohort	dose d	$Y_{b}$
(a) Initial1 (2 cohorts)		
1	$d_{1}$	(0, 0, 0)
2	$d_{2}$	(0, 0, 1)
(b) Initial3 (7 cohorts)		
1	$d_{1}$	(0, 0, 0)
2	$d_{2}$	(0, 0, 1)
3	$d_{2}$	(0, 0, 0)
4	$d_{3}$	(0, 0, 1)
5	$d_{3}$	(0, 0, 0)
5	$d_{4}$	(0, 0, 0)
7	$d_{5}$	(0, 1, 0)

*Note:* Panel (a) presents Initial1 comprising 2 cohorts, and panel (b) presents Initial3 comprising 7 cohorts, each listing the cohort number, administered dose d, and observed toxicity outcomes $Y_{b}$.

Figure 5 illustrates how the choice of the initial stage influences the results of the dose‐finding design. Every aspect other than the choice of initial stage of the hypothetical trial is comparable to that in Section 3.1. With the Initial3, both the Probit and Joint2d methods successfully avoid the anchoring problem in Figure 1 and Figure 2, progressing to $d_{5}$ by the end of the trial. The Joint9d method behaves similarly to its performance in Figure 2, where there is no estimation problem with both Initial1 and Initial3. This example also suggests that the Joint9d method exhibits robustness to the complexity of the initial stage, while the anchoring problem of the Probit and Joint2d methods can be avoided by a more complicated initial stage. Similarly, this estimation problem can be alleviated under Inital2 as shown in Supporting Information 11.

**FIGURE 5:**
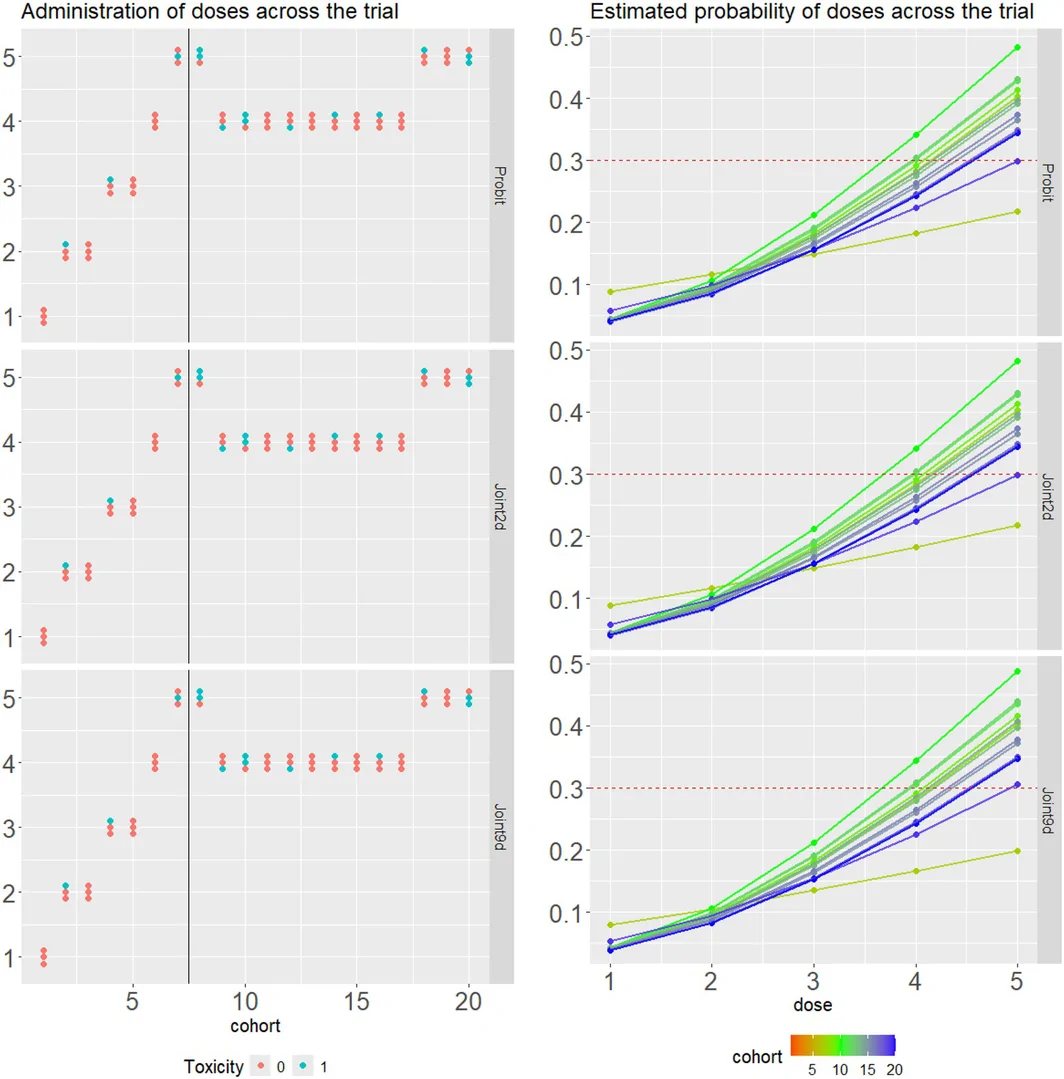
Illustration of a hypothetical trial without the anchoring problem following the implementation of an alternative initial stage (Initial3). Stage 1 includes seven cohorts, and Stage 2 applies methods based on the two‐parameter probit model.

To thoroughly assess the impact of the initial stage, we evaluate the PCS of the Probit method across various scenarios using different initial stages, as introduced in Section 4.1. For comparison, we also include simulation results for the Empiric method, as previously investigated in related studies [29, 49]. Corresponding dose‐finding algorithm is the same as described in Section 2.2. Unlike the Probit and Logistic models, which may fail to generate reasonable estimates under specific data configurations, the Empiric model exhibits greater robustness, consistently producing reliable estimates across different initial stages. A comparison of model choices by Paoletti and Kramar [33] concludes that a one‐parameter empiric (power) model outperforms a two‐parameter logistic model. This superiority becomes more pronounced as sample sizes increase, reinforcing the finding that the separation problem in the two‐parameter probit or logistic models emerges early in the trial and persists, as discussed in Section 3.1.

Simulation results for the PCS of the Empiric method under various initial stages are presented in Supporting Information 12. These results indicate that the performance of the Empiric method is largely unaffected by the choice of initial stage. Consequently, we report only the results under Initial1 in Figure 6, illustrating that the performance of this method is less sensitive to initial conditions than those of the two‐parameter logistic and probit models.

**FIGURE 6:**
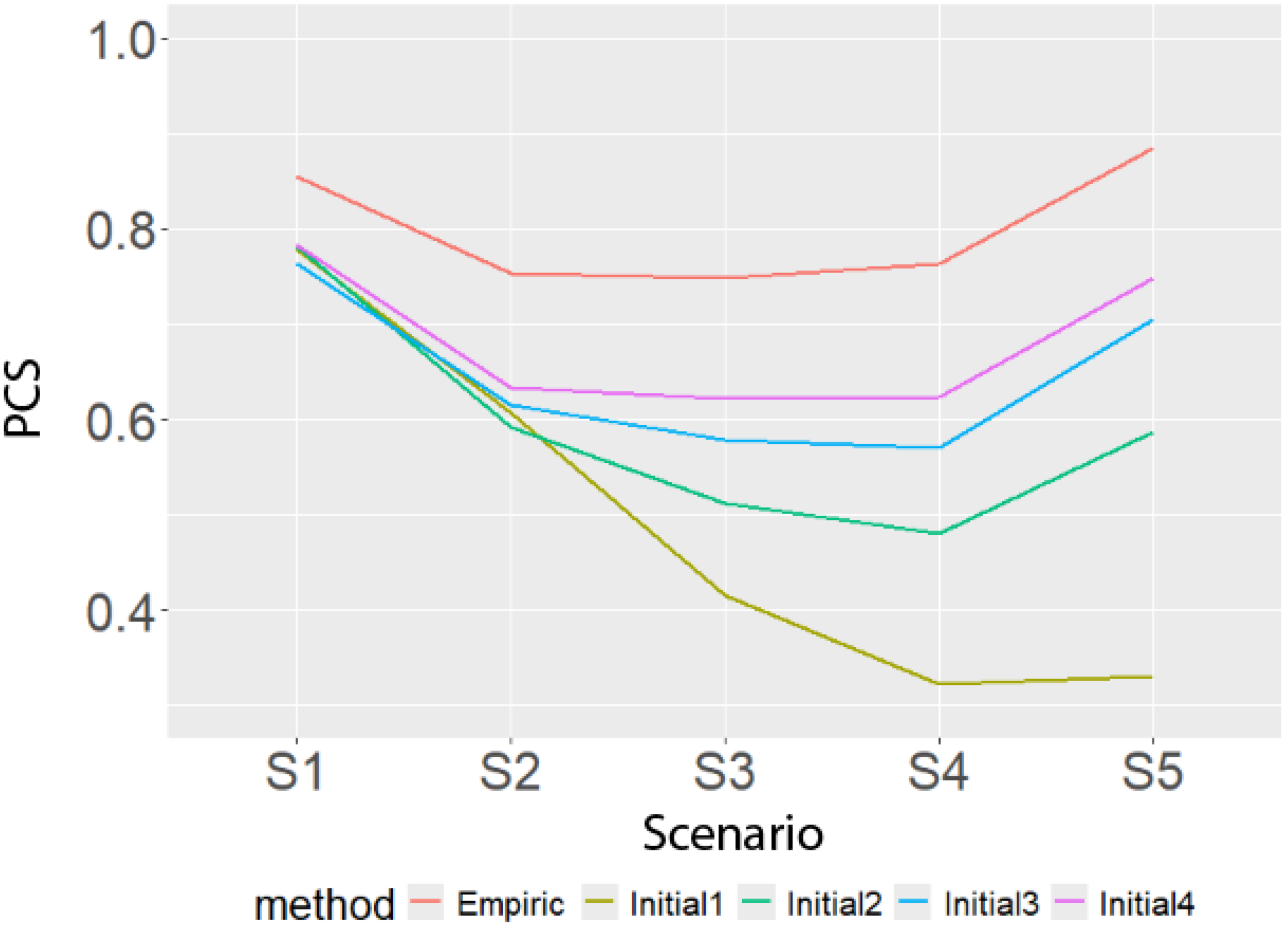
PCS of the Probit method across different scenarios and initial stages, with Empiric results included for reference. Different colours correspond to different methods, the x‐axis represents different scenarios, and the y‐axis represents the corresponding PCS values.

Figure 6 illustrates that the PCS of the Probit method in more challenging scenarios, where the target dose $d^{T}$ is at higher dose levels, can be substantially low if the initial stage is not sufficiently complex. For instance, in scenarios $S_{4}$ and $S_{5}$ under Initial1, fewer than 40% of trials correctly identify the target dose, a level of performance that is suboptimal for clinical application. This observation is consistent with the findings of Paoletti and Kramar [33], who note that “The situation of the highest dose being the MTD is not representative of the performance of the methods. The two‐parameter model never picks up the right dose when using a likelihood estimator”. In most investigated scenarios, except for $S_{1}$, the PCS of the Probit method improves with increased complexity in the initial stage. In scenario $S_{1}$, where the target dose corresponds to the lowest dose level, the PCS appears relatively insensitive to the complexity of the initial stage, even when the dose–toxicity curve exhibits an undesirable step function.

The Empiric model consistently outperforms the Probit method across the investigated scenarios, as demonstrated in Paoletti and Kramar [49]. An often overlooked advantage of the Empiric method is its capacity to mitigate the separation issue in parameter estimation, thereby maintaining stable performance across varying initial stages. This allows it to serve as a key benchmark to complement our analysis: its one‐parameter structure requires only heterogeneity in outcomes for identifiability, and as a result it exhibits zero separation across all scenarios and initial stages.

Despite the consistency across different initial stages and no estimation problem in the Empiric method, We maintain the Probit method as the primary comparator for the following reasons: (1) Methodological consistency—using the same two‐parameter probit structure across all methods (Probit, Joint2d, Joint9d) isolates the contribution of biomarker information without confounding from model changes; (2) Challenging conventional assumptions—our calibration results (see Section 5.6) show calibrated Probit achieves comparable performance to the empiric model after dose label calibration, challenging the assumption that the empiric model is inherently superior [33].

It is important to note that there is no universal standard for the complexity of the initial stage for the dose‐finding studies. The choice should depend on the sample size of the trial while also considering the inherent separation problem. In contrast, for the Empiric method (see Supporting Information 12), more complex initial stages provide no added benefit in terms of the proportion of separation or PCS, though they come at the cost of patient outcomes during the initial stage.

The trade‐off between the benefit of a smaller proportion of separation and the cost of a larger initial stage for the Probit method is illustrated in Table 6. As shown in Table 6a, in four out of five scenarios, more than 50% of simulations result in a high degree of separation when the heterogeneity requirement is implemented using Initial1. This issue is substantially mitigated under Initial2. While a more complex initial stage can effectively reduce the separation problem, the associated cost in terms of time and patients should not be overlooked (as shown in Table 6b). For instance, with Initial4, the initial stage consumes more than one‐quarter of the total sample size on average before the method can proceed in over half of the evaluated scenarios.

**TABLE 6:** Performance trade‐offs of the Probit method under increasingly complex initial stages.

	Initial1	Initial2	Initial3	Initial4
(a) Proportion of the separation				
$S_{1}$	0.25	0.23	0.23	0.19
$S_{2}$	0.57	0.48	0.43	0.32
$S_{3}$	0.61	0.41	0.35	0.24
$S_{4}$	0.83	0.60	0.48	0.37
$S_{5}$	0.88	0.59	0.46	0.40

(b) Average number of cohorts in the initial stage				
$S_{1}$	1.40	2.42	3.12	3.95
$S_{2}$	1.86	3.01	4.05	5.06
$S_{3}$	2.29	3.72	4.89	6.00
$S_{4}$	3.00	4.58	5.87	7.01
$S_{5}$	3.52	5.44	7.00	8.44

*Note:* Panel (b) reports the average number of cohorts in the initial stage across simulations; non‐integer values reflect simulation averages and do not imply that fractional cohorts occur in practice.

Having established Initial3 as the preferred configuration for mitigating estimation problems in the Probit method, we now extend the investigation to the biomarker‐informed joint models. The following results examine the performance of all three methods—Probit, Joint2d, and Joint9d—under the Initial3 configuration, assessing whether the benefits observed for the Probit method carry over to the joint modelling framework and whether the incorporation of biomarker information provides additional gains in dose selection accuracy.

Figure 7a presents the PCS results for all three methods across all scenarios under the Initial3 configuration, with the Empiric method included as an upper benchmark (shown as points). For clarity, the Probit method in Figure 7a displays results under $\rho_{b}=0$ consistently across all panels, as the Probit method does not incorporate biomarker information and its PCS is therefore independent of $\rho_{b}$ by design. There is potential variation in Probit PCS across different $\rho_{b}$ settings in the full simulation results is attributable solely to simulation error, remaining within 2% across all scenarios; complete results are provided in Supporting Information 13. This presentation ensures a direct correspondence between the plotted values in Figure 7 and the numerical results in Figure 7b. When the biomarker is purely noise ($\rho_{b}=0$), the PCS results show negligible differences among these methods, with most cases differing by less than 2%. However, as biomarker becomes more informative (i.e., $\rho_{b}=0.4$ and $\rho_{b}=0.8$), significant differences in PCS emerge across the methods. Figure 7b presents the PCS across five scenarios ($S_{1}$‐$S_{5}$) under varying levels of correlation between binary and continuous outcomes ($\rho_{b}=0,0.4,0.8$).

**FIGURE 7:**
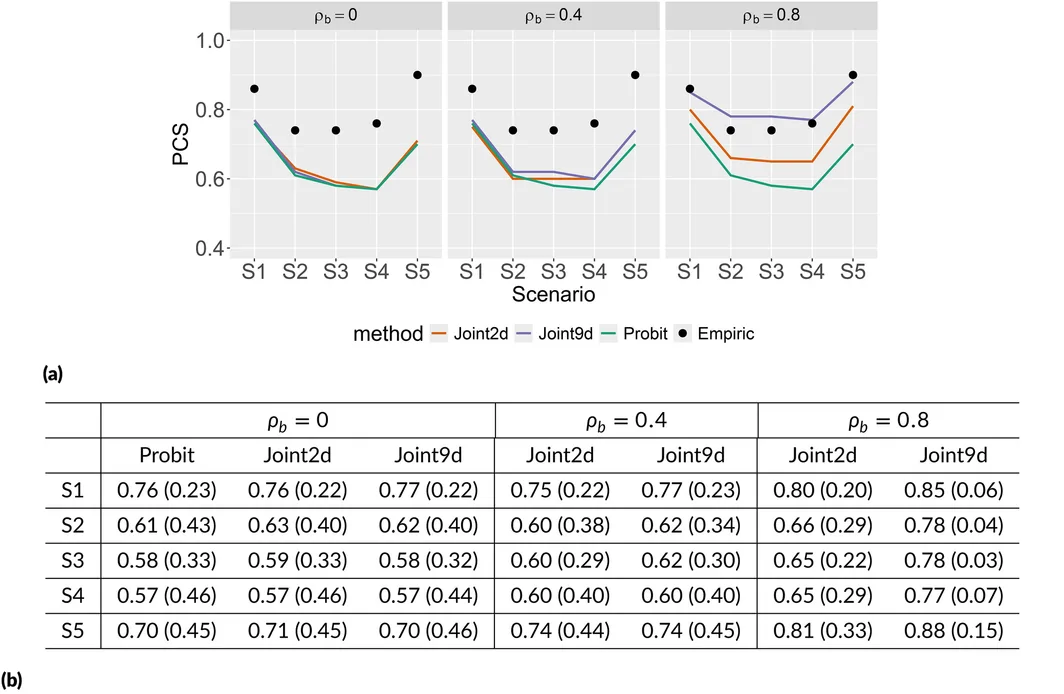
PCS results across five scenarios ($S_{1}$‐$S_{5}$) and $\rho_{b}\in \{0,0.4,0.8\}$ with all three methods implementing the initial stage Initial3. (a) PCS results across scenarios; the empiric method shown as points for reference ($S_{1}-S_{5}$: 0.86, 0.74, 0.74, 0.76, 0.90). (b) PCS results; proportions of separation in parentheses. The Empiric method is excluded as separation does not arise under its one‐parameter structure.

Overall, both joint modeling approaches (Joint2d and Joint9d) consistently outperform the baseline Probit method, particularly as the correlation $\rho_{b}$ increases, progressively closing the gap with the Empiric benchmark. Specifically, Joint2d yields a 3‐10% absolute improvement in PCS across scenarios from $\rho_{b}=0$ to $\rho_{b}=0.8$, while Joint9d achieves a 10‐20% improvement. This suggests that the joint models are better able to leverage the underlying correlation structure between toxicity and biomarker. In scenario $S_{1}$, which represents a relatively easy case where all methods perform well, yielding more than 75% of PCS, the Joint2d and Joint9d methods still achieve incremental improvements of 4‐9%, reaching a PCS of 80% and 85%, respectively, at $\rho_{b}=0.8$, approaching the Empiric benchmark of 86%. In contrast, scenario $S_{5}$ represents the most challenging case, where the benefits of joint modeling are most evident: Joint2d improves PCS from 0.71 to 0.81 (an 10% gain), and Joint9d from 0.70 to 0.88 (an 18% gain), as $\rho_{b}$ increases from 0 to 0.8, with Joint9d closely approaching the Empiric result of 0.90. In such difficult scenarios, we also focus on the proportion of separations (discussed in Section 3.1).

Simulation results for the proportion of separation, reported alongside PCS values, are summarized in Figure 7b. A high separation rate indicates that the model frequently fails to capture the dose–toxicity relationship as the failure of finding the MLE. Under the least informative setting ($\rho_{b}=0$), all methods exhibit high separation rates, particularly in the more challenging scenarios $S_{4}$ and $S_{5}$, where the separation proportion exceeds 40%. As the related information of biomarker increases (e.g., $\rho_{b}=0.8$), both the accuracy of dose selection (PCS) and the stability of the model (separation rate) improve significantly. Among the methods evaluated, Joint9d exhibits the most pronounced reduction in separation. In scenario $S_{5}$, for instance, the separation rate decreases from 45.3% to 14.9%, and falls below 10% in most scenarios, reaching as low as 7.4% in $S_{4}$, 2.8% in $S_{3}$, and 3.6% in $S_{2}$. These results indicate that incorporating biomarker information is highly effective in mitigating the separation problem across a range of scenarios. In contrast, scenario $S_{1}$ consistently shows low separation rates across all methods and $\rho_{b}$ values. This suggests that in simpler settings, where informative data accumulate rapidly at lower dose levels, the anchoring issue has limited influence on the final dose recommendation, as early‐stage information at the lowest dose is sufficient to guide correct selection.

The issue of equal estimated probabilities, as described in Section 3.2, is minimal across all methods and scenarios, with most instances occurring in less than 1% of cases (see Table 7). This phenomenon is largely data‐driven and tends to arise under specific conditions, most notably when the target dose $d^{T}$ is either correctly identified early at a lower dose (e.g., scenario $S_{1}$) or when there is a general lack of observable treatment response across all dose levels (e.g., scenario $S_{5}$).

**TABLE 7:** Proportion of equal estimated probabilities.

	$\rho_{b}=0$			$\rho_{b}=0.4$		$\rho_{b}=0.8$	
	Probit	Joint2d	Joint9d	Joint2d	Joint9d	Joint2d	Joint9d
S1	0.007	0.007	0.008	0.010	0.011	0.012	0.025
S2	0	0	0	0	0	0.003	0.007
S3	0	0	0	0	0	0	0.007
S4	0	0	0	0	0	0	0.006
S5	0.002	0.004	0.003	0.005	0.003	0.003	0.007

*Note:* All three methods were implemented with Initial3 as the initial stage. Values are reported to three decimal places owing to the small magnitude of this proportion.

The issue is slightly more prevalent in the Joint9d model under $\rho_{b}=0.8$, particularly in scenario $S_{1}$, where the observed rate of equal estimated probabilities reaches 2.5%. This increase is likely due to the amplification of patterns in binary outcomes by highly informative biomarker data. When biomarker values provide similar predictive strength as the binary outcomes, they may reinforce existing uncertainties or similarities between dose levels, making it harder to distinguish between competing options.

It is important to emphasize that dose‐finding studies prioritize correct dose selection over precise parameter estimation. The presence of equal estimated probabilities does not hinder the trial's progression or its ability to correctly identify the optimal dose. This is ensured by design features such as the no‐skipping rule, which enforces logical and structured dose escalation/de‐escalation. Consequently, even in the rare cases where estimated probabilities of different doses are similar, all methods remain capable of recommending the correct target dose with high reliability.

Early stopping due to observed toxicities in the initial stage occurs in a small proportion of cases (See Supporting Information 14). This is more pronounced in scenario $S_{1}$, but overall, early termination due to overtoxicity is rare and does not pose a significant concern for the proposed design. Because all three methods share the same initial stage, this metric remains consistent across methods.

### 5.5 One‐Parameter Probit Method

Before using the one‐parameter Probit method in the context of dose‐finding setting, we first apply the method for all available data for 20 cohorts of patients with all 5 dose levels for parameter estimation. Figure 8a illustrates the point estimates of $\hat{\pi }(x_{j})$ for each dose across different scenarios, obtained using the one‐parameter Probit method with varying values of $a_{0}$. When $a_{0}=0$, the reference level $x^{*}$ can dichotomize dose labels between $d_{3}$ and $d_{4}$ at $\pi (x^{*})=0.5$. This results in doses $d_{1}$ through $d_{3}$ being estimated to below 0.5, while $d_{4}$ and $d_{5}$ are consistently estimated as more toxic than 0.5 across all scenarios. Consequently, the method performs well only under scenario $S_{2}$, where the dose‐toxicity relationship aligns with the pre‐specified skeleton. In contrast, it leads to underestimation in scenario $S_{1}$ and overestimation in scenarios $S_{3}$ through $S_{5}$ as shown in Supplement Information 8.

**FIGURE 8:**
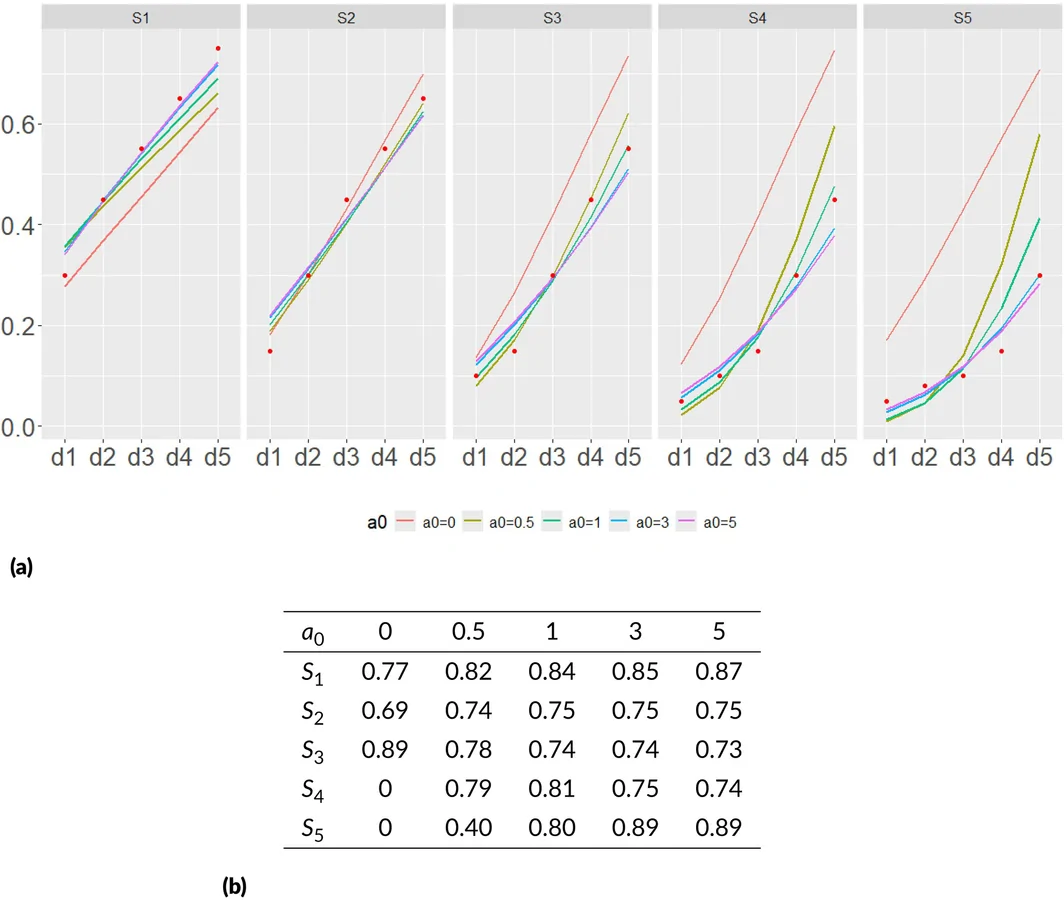
Average estimated toxicity probability and PCS across five scenarios ($S_{1}-S_{5}$) under varying values of $a_{0}$ in the one‐parameter Probit model, with Initial1 as the initial stage. (a) Estimated toxicity probability across scenarios and dose levels under varying $a_{0}$. True toxicity probabilities are shown as dots. (b) PCS under varying $a_{0}$; complete dose information assumed.

For all other choices of $a_{0}$ illustrated in Figure S13 in Supplement Information 8, the reference dose $x^{*}$ no longer dichotomizes the dose labels. Instead, it serves as a reasonable upper limit. When $a_{0}=0.5$, the upper limit $\pi (x^{*})=0.691$ leads to an overestimation of the probability of toxicity for higher doses in scenarios where all doses are relatively safe, such as $S_{4}$ and $S_{5}$. Furthermore, the reference dose $x^{*}=0$ causes the estimated probability of toxicity for higher doses, particularly $d_{5}$, to be biased toward $\pi (x^{*})$. Figure 8a suggests that a small value of $a_{0}$ is unsuitable. Instead, an appropriately large value of $a_{0}$ should ensure that $\pi (a_{0})\approx 1$ at $x^{*}=0$, preventing misleading information from locating the curve via the reference dose $x^{*}$.

In dose‐finding studies, the choice of initial stage has only a marginal impact on the PCS for one‐parameter models, including both the empiric (see Section 5.4) and one‐parameter Probit models. This is because issues such as the anchoring problem and the broader separation issues are specific to two‐parameter sigmoid models, namely, the two‐parameter Probit and Logistic models. We consider the results under Initial1 here.

The dichotomization of dose levels permits accurate identification of the target dose ($d^{T}$) only when the prespecified skeleton aligns precisely with the true dose‐toxicity relationship, as exemplified by scenario $S_{3}$ with $a_{0}=0$ (see Figure 8b). In contrast, the model fails to correctly identify $d^{T}$ under scenarios $S_{4}$ and $S_{5}$, as the true target doses ($d_{4}$ and $d_{5}$, respectively) are erroneously perceived as overly toxic, with estimated toxicity probabilities exceeding the reference level of $\pi (x^{*})=0.5$. Similarly, the specification $a_{0}=0.5$ yields a notably low PCS in $S_{5}$ due to an overestimation of the toxicity of $d_{5}$. Conversely, higher values of $a_{0}$, specifically $a_{0}=1$, $a_{0}=3$, and $a_{0}=5$, demonstrate more robust performance, consistently achieving higher PCS values and accurately identifying the target dose across all examined scenarios. As mentioned in Section 4.2, a large value of $a_{0}$ can easily lead to an extreme argument in the probit model, causing the estimated toxicity probabilities $\pi (d_{j})$ to approach 1 for all doses.

Chevret [50] investigated the impact of the choice of $a_{0}$ for the one‐parameter logistic model within the Bayesian framework, highlighting that the best choice depends on the situation tested. Moreover, it was noted that for any given situation, the bias of the estimated probability of toxicity was not linear and the recommendation of a fixed value for $a_{0}$ is somewhat difficult and arbitrary.

When compared to the results of the two‐parameter probit model, as shown in Figure 7b, there is a consistent improvement in PCS across various scenarios. Furthermore, no instances of the separation problem are observed, as in the empiric model, in Supporting Information 12.

Unfortunately, implementing the one‐parameter probit model within joint models is not feasible because the marginal effects of interest are derived from the conditional model (see Equation 6). Consequently, the dose label transformation must be based on a scaled intercept rather than a fixed intercept. This scaling accounts for the conditional variance, $1-\rho_{b}^{2}$, as shown in Equation (7). As a result, the intercept used in the joint model is influenced by the value of $\rho_{b}$. The transformation based on $a_{0}$ (see Table 1) fails to properly align the dose labels with the skeleton. In other words, joint methods cannot apply the same dose label transformation as the Probit method.

The one‐parameter probit model serves as a comparator alongside the widely used Empiric (power) model [29, 33, 49], allowing us to evaluate whether the numerical difficulties we observe—particularly separation problems and convergence issues—arise specifically from the two‐parameter structure. We acknowledge that this model cannot be directly extended to the joint modeling setting due to the incompatibility of its fixed intercept with the factorization framework, and its role is therefore limited to a diagnostic comparator.

### 5.6 Simulation Results With Calibrated Dose Labels

We perform calibration of dose labels based on both the model parameters and the skeleton of doses, and then select the set of dose labels that optimizes the geometric mean of the PCS results across the investigated scenarios, using the calibrated skeleton and model parameters. The grid search for the calibrated skeleton and model parameters is carried out through an iterative process, starting with a broad range of values and progressively refining them to identify the optimal combination. For each combination of skeleton and model parameters, the PCS is calculated for each scenario presented in Table 4b. The geometric mean of PCS across all investigated scenarios is then computed. The combination that achieves the highest PCS is selected, and the corresponding dose labels are determined based on the chosen method. The grid values can be informed by prior experience, if available. Figure S18 in Supporting Information 15 presents the model and corresponding dose labels both before and after calibration. Note that the calibration procedure is specific to each method and each value of $\rho_{b}$.

Figure 9 shows the simulation results for the same example trial presented in Figure 1, analyzed with the Probit method after dose label calibration. Calibration aligns the dose labels more accurately with the underlying dose‐response curve, causing the calibrated labels in this hypothetical trial to cluster toward the lower end of the curve (see Figure S18). This adjustment resolves the issue of the Probit method becoming anchored at incorrect dose levels, enabling successful escalation to $d_{4}$. Furthermore, after calibration, the estimated dose–response relationship transitions smoothly as a curve rather than exhibiting a step‐function pattern. Thus, calibrating the dose labels can also help mitigate the separation problem observed in Figure 1.

**FIGURE 9:**
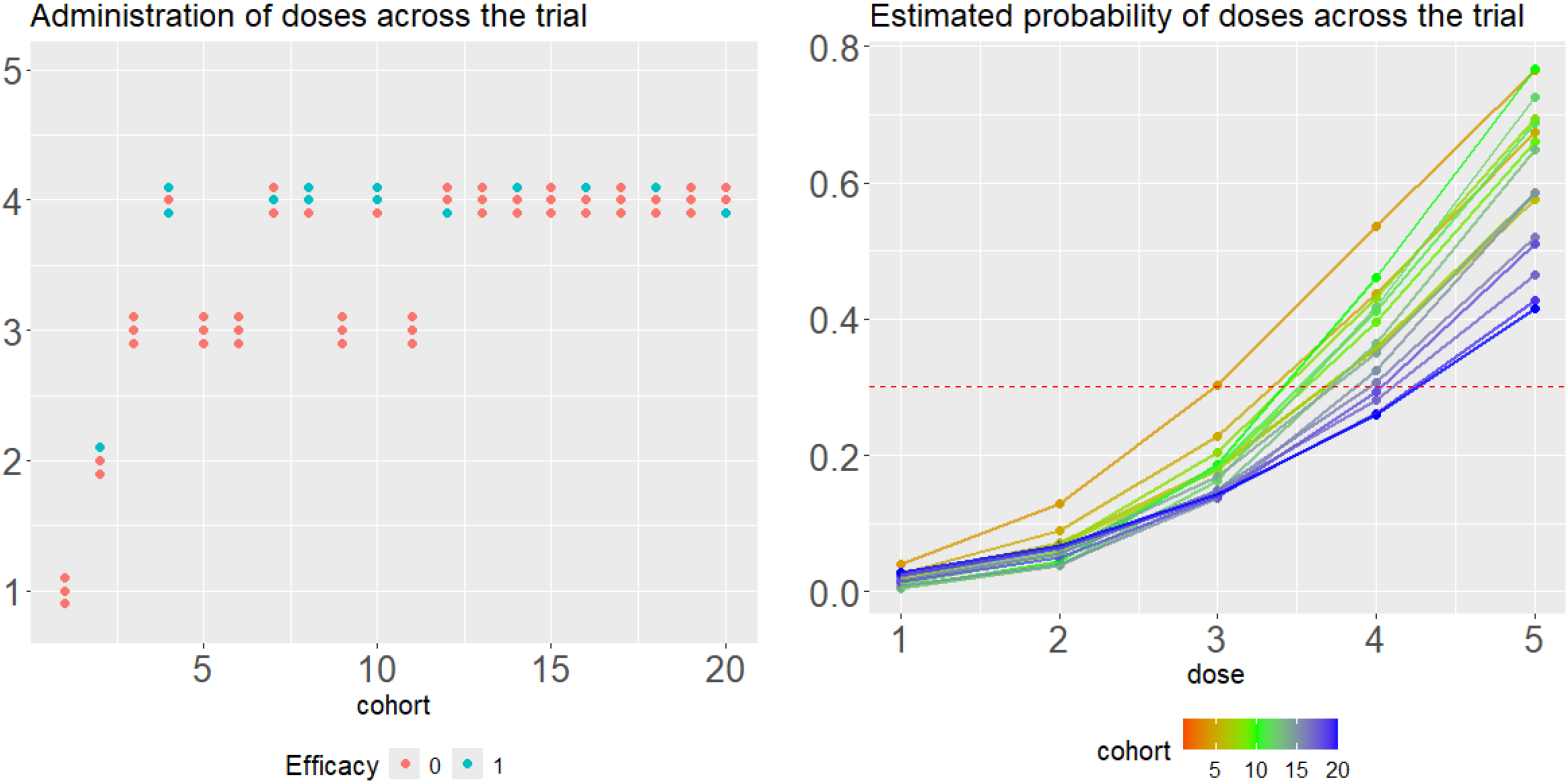
Illustration of the hypothetical trial without the anchoring problem after calibration for dose labels (Initial3).

Because Figure 5 demonstrates the influence of a more complex initial stage on estimation results, we additionally applied the same calibrated dose labels under the Initial1 configuration to isolate the effect of calibration itself. The corresponding results, shown in Supporting Information 16, indicate that calibration alone can resolve the separation issue in this individual example.

In larger simulations, the PCS results for the Probit method, shown in Figure 10a, demonstrate substantial improvement after calibration. Following calibration, the performance of the Probit method becomes comparable to that of the Empiric method. Importantly, this finding suggests that the widely accepted conclusion regarding the empiric model's superior performance, drawn from simulation studies conducted without calibrating the Probit model [33], may be incomplete.

**FIGURE 10:**
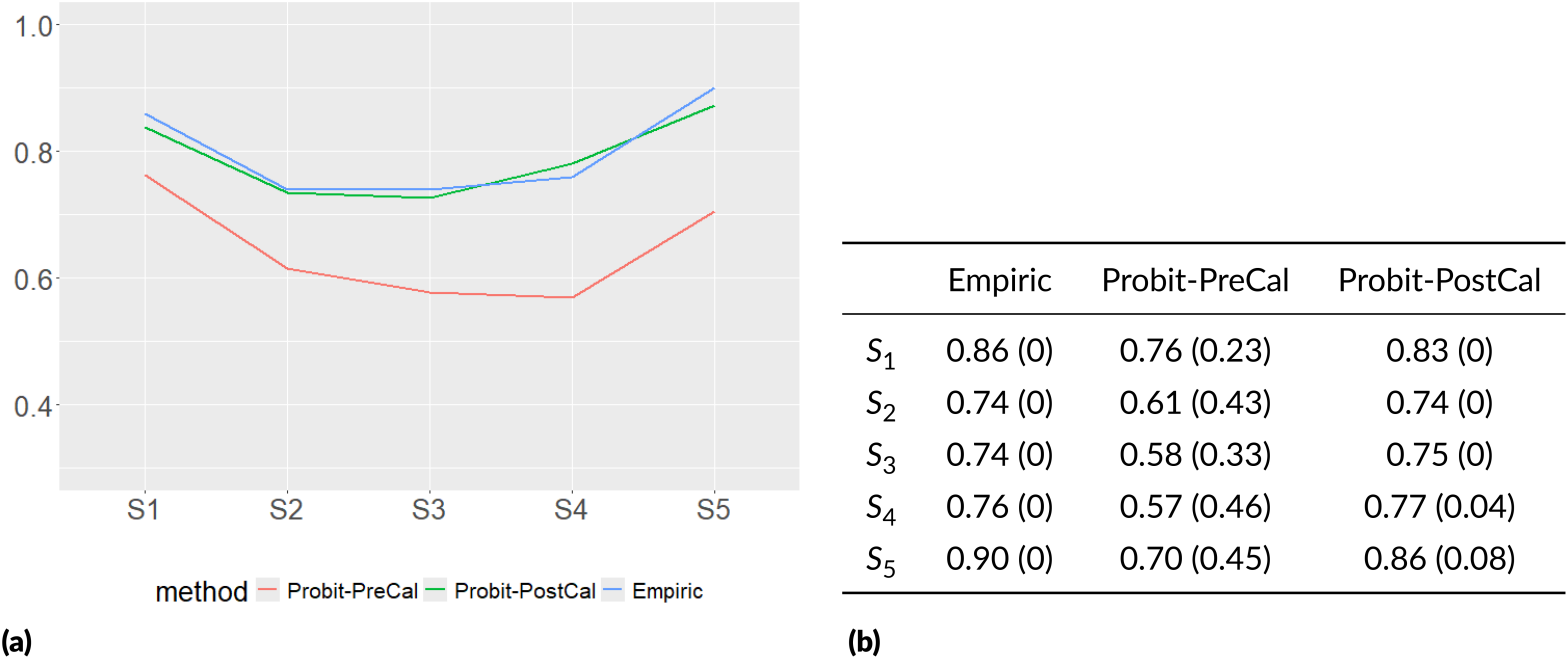
PCS results of the Probit method before calibration (Probit‐PreCal) and after calibration (Probit‐PostCal). The proportion of separation is included in the parentheses. (a) PCS values before and after calibration; Empiric as reference. (b) PCS results with the proportions of separation.

Calibration also helps mitigate the separation problem by aligning dose labels more accurately along the sigmoid curve (see the proportion of separation in Figure 10b). This adjustment is particularly impactful in challenging scenarios where the target dose $d^{T}$ is the highest dose and lower doses exhibit low probabilities of toxicity (as in scenario $S_{5}$), making early toxic responses less likely. In such cases, calibration distributes dose labels more densely across the lower region of the curve, reflecting lower probabilities $\pi (x_{i})$ for these doses. Consequently, the proportion of separation is largely reduced from 44.2% to less than 1% (see $S_{5}$ in Figure 10b). While the Empiric method has often been viewed as superior to the two‐parameter Probit model, our findings indicate that, with properly calibrated dose labels, the Probit method. Since the Empiric method is structurally immune to the separation problem, it serves as a natural reference point for the best achievable performance in the absence of estimation issues. Comparing the calibrated Probit against this benchmark therefore directly quantifies how much of the performance gap between the two models is attributable to separation rather than to inherent modelling differences. As shown in Figure 10b, calibration substantially improves the performance of the Probit method across all scenarios. Following calibration, Probit‐PostCal achieves PCS values of 0.83, 0.74, 0.75, 0.77, and 0.86 in scenarios $S_{1}-S_{5}$ respectively, compared to the Empiric method (0.86, 0.74, 0.74, 0.76, and 0.90). Probit‐PostCal performs comparably to the Empiric method in most of scenarios Although this study does not conduct calibration to the Empiric model, future work exploring dose label optimization in that framework could potentially enhance its performance further.

Figure 11a shows the simulation results for all three methods after calibration. For clarity and direct correspondence with Figure 11b, the Probit method is displayed under $\rho_{b}=0$only, as its PCS is independent of $\rho_{b}$ by design; minor variation across $\rho_{b}$ values reflects simulation error only. The PCS of all methods improves significantly compared to pre‐calibration results (see Figure 7a and Figure 7b). The improvement is most pronounced in the Probit method, where PCS increases by more than 10% in four out of five scenarios. This highlights the critical role of dose label calibration. For the Joint2d method, the benefit of incorporating biomarker values, as shown in Figure 7a, diminishes after calibration. Specifically, the additional information from a single biomarker is insufficient to compensate the added complexity of the model. Similarly, for the Joint9d method, the differences in PCS across $\rho_{b}$ values diminish after calibration. However, the advantage of incorporating informative biomarker (e.g., $\rho_{b}=0.8$) remains evident, particularly in scenarios $S_{2}$‐$S_{4}$.

**FIGURE 11:**
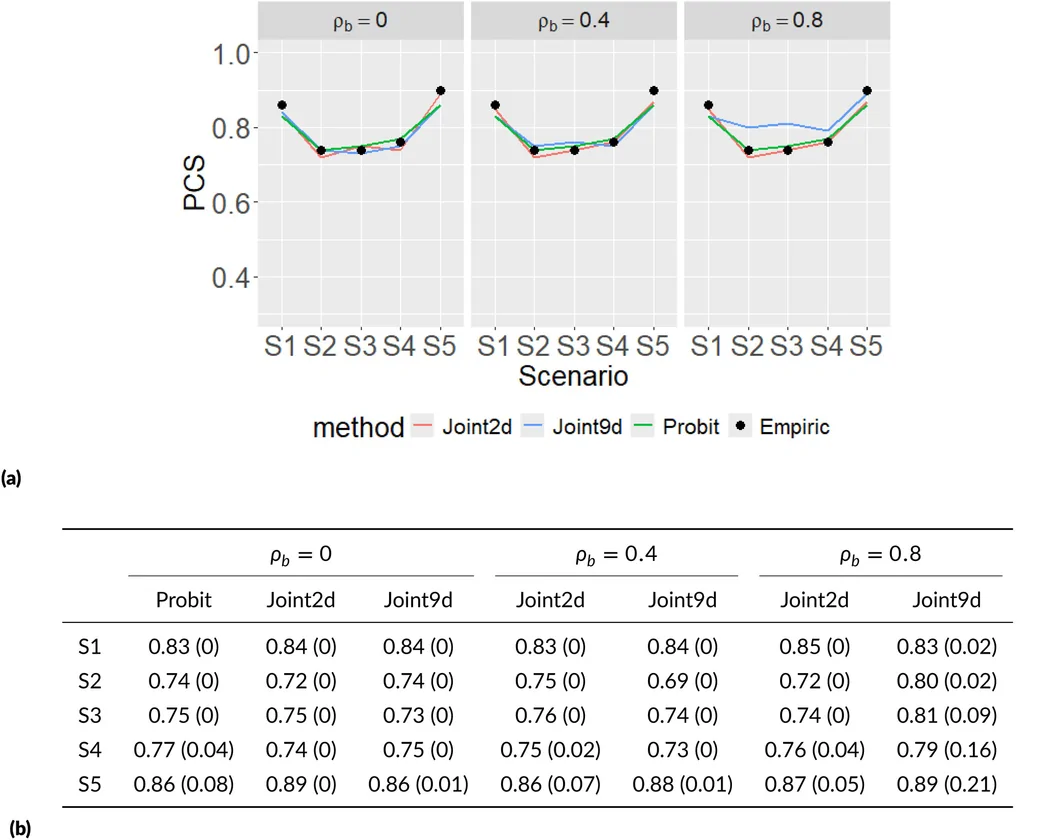
PCS results across five scenarios ($S_{1}-S_{5}$) and $\rho_{b}\in \{0,0.4,0.8\}$ with all three methods implementing the initial stage Initial3. (a) PCS results across scenarios with calibrated dose labels; Empiric method shown as points for reference ($S_{1}-S_{5}$: 0.86, 0.74, 0.74, 0.76, 0.90), with original dose labels and Initial1 as the initial stage. (b) PCS results; proportions of separation in parentheses.

Another key advantage of the joint models over the Probit model is their substantial reduction in separation. For the Probit method, calibrating the dose labels significantly mitigates separation, as shown in Figure 11b: in three out of five scenarios, fewer than 1% of simulations exhibit separation in three out of five scenarios. In the most challenging scenario, $S_{5}$, separation decreases substantially from the 5% before calibration to just 8% after. The joint models show a similarly notable reduction in separation across most scenarios following calibration. Comparing results before and after calibration reveals that poor performance in difficult settings like $S_{5}$, is largely driven by the anchoring problem, a previously overlooked issue in the CRM literature. Importantly, many of the separation cases observed prior to calibration lead to correct dose selection once calibration is applied.

Overall, the anchoring issue in dose‐finding studies, or the separation problem in parameter estimation, arises from the limited data typically available early in trials, which is often insufficient to accurately characterize the target dose–response curve. Calibrating the dose labels helps align the investigated dose levels more appropriately along this curve. Our findings indicate that dose label calibration is critical for the performance of likelihood‐based approaches in dose‐finding studies.

Moreover, the simulation results for the Joint9d method demonstrate that the proposed joint model remains highly robust when informative biomarkers are available (i.e., $\rho_{b}=0.8$). In this scenario, dose labels remain sparse even after calibration (see Figure S18). Nonetheless, the PCS results are quite similar before and after calibration, with some observed improvement. These findings underscore an additional advantage of incorporating biomarkers through joint modeling, as it helps mitigate challenges associated with the problem of separation (see Section 3).

Table 8 shows the proportion of trials yielding equal estimated probabilities after calibration. Consistent with the pre‐calibration results in Table 7, scenarios $S_{1}$ and $S_{5}$ remain more susceptible to this issue than other scenarios. Among the methods analyzed, only the Joint2d and Joint9d approaches exhibit an increased proportion of equal estimates under $\rho_{b}=0.4$ and $\rho_{b}=0.8$.

**TABLE 8:** The proportion of equal estimated probability after calibration.

	$\rho_{b}=0$			$\rho_{b}=0.4$		$\rho_{b}=0.8$	
	Probit	Joint2d	Joint9d	Joint2d	Joint9d	Joint2d	Joint9d
S1	0.002	0	0.002	0.042	0.002	0.035	0.049
S2	0	0	0	0.006	0	0.004	0.013
S3	0.003	0	0	0.010	0	0.008	0.008
S4	0	0.002	0	0.008	0	0.016	0.006
S5	0.008	0.004	0.006	0.024	0.008	0.060	0.002

*Note:* Values are reported to three decimal places owing to the small magnitude of this proportion.

As discussed in Section 3, the occurrence of equal estimated probabilities is better regarded as a phenomenon rather than a fundamental flaw, as it can still reflect meaningful data patterns and support accurate dose recommendations. The increase in the proportion of equal estimated probability observed under $\rho_{b}=0.4$ and $\rho_{b}=0.8$ suggests that high correlation in biomarker information can amplify these patterns, making equal estimated probabilities more frequent. In other words, when the data lack sufficient information about the differences, the model may struggle to differentiate between dose levels, when compared to the Probit method.

This highlights an important limitation of dose label calibration: while it can mitigate certain estimation challenges, such as separation, by creating more densely spaced labels, it cannot fully overcome the issues posed by sparse or limited data. In some cases, it can even exacerbate these issues, as the calibrated dose labels may become more densely distributed than before calibration (see Figure S18).

### 5.7 Simulation Results for Smaller Sample Sizes

Simulation results for all methods—Empiric, Probit, Joint2d, and Joint9d—across scenarios and $\rho_{b}$ values under a reduced sample size are provided in Supporting Information 17. Specifically, we consider N=30, corresponding to 10 cohorts of size 3. The same general patterns as in the main analysis above are observed.

First, Joint9d consistently achieves higher PCS than Joint2d and the Probit method when there is informative information from biomarker (i.e., $\rho_{b}=0.8$), regardless of whether original or calibrated dose labels are used. Second, Joint9d outperforms even the Empiric method under strong biomarker correlation, reinforcing our earlier finding that Joint9d is a strong competitor to the Empiric method. Third, the magnitude of improvement from Probit and Joint2d to Joint9d is smaller under the reduced sample size, suggesting that the higher‐dimensional parameter space of Joint9d requires more patients to fully realize its advantage. Finally, the calibrated dose labels used here were derived under the larger sample size (N=60); PCS results under calibration could therefore be further improved if calibration were performed specifically for N=30, which we leave for future investigation.

## 6 Discussion

Incorporating biomarker information into dose‐finding studies in oncology can not only improve the probability of correct dose selection but also mitigate estimation challenges that are common yet often overlooked in frequentist frameworks. A particularly instructive finding was that the poor performance of the two‐parameter Probit model in challenging scenarios was largely attributable to the anchoring problem arising from sparse data rather than to any fundamental limitation of the model itself. While this might suggest that a Bayesian framework—which can regularize estimation through prior information—would be more naturally suited to such settings, the frequentist approach remains attractive for its transparency and freedom from prior specification; the calibration and biomarker integration strategies proposed here represent complementary solutions to data sparsity within this framework.

In applying a likelihood‐based estimation method, it is essential to prevent parameter estimates from reaching the boundaries of the parameter space and ensure that the likelihood function is not monotonic. Although the well‐established heterogeneity requirement is designed to address these challenges, our findings show that simply meeting this condition is insufficient to guarantee the existence of MLEs in two‐parameter models. In particular, when lower doses exhibit no toxicity, estimation issues such as the separation problem may still arise, leading to a step function in the estimated probability and potentially anchoring the trial at lower doses. This highlights the need for additional strategies to ensure robust parameter estimation. Furthermore, depending on the data, equal estimated probabilities may occur, adding further complexity to the estimation process.

Among the extensive literature on CRM, various simulation studies explore the selection of key design parameters for both Bayesian and likelihood approaches. Our work highlights the inherent limitations in early dose‐finding studies from the perspective of data configuration. While our findings align with prior research, we also provide deeper insights into the challenges encountered in more complex scenarios, emphasizing the importance of careful application of model‐based designs in dose‐finding studies.

One potential approach to mitigating these estimation challenges involves refining the initial stage of model fitting by requiring more occurrences before initiating the model fitting process. In the case of one‐parameter models, the choice of the fixed intercept $a_{0}$ in the one‐parameter Probit model plays a crucial role in performance. Our findings align with those of Chevret [50]. However, the one‐parameter model with a fixed intercept cannot be directly extended to the joint method due to the lack of dose label transformations compatible with repeated biomarker measurements. Dose label calibration, which aligns dose labels more accurately along the dose‐response curve, was shown to effectively enhance PCS and reduce estimation issues in both the Probit and joint methods. Despite these improvements, certain scenarios still faced challenges due to the inherent limitations of the dataset, highlighting the constraints of likelihood‐based approaches in early‐phase dose‐finding studies.

Ideally, when data are sufficiently informative, parameter estimates remain consistent regardless of the optimization algorithm used. However, with limited data, different algorithms can produce substantially divergent estimates, potentially leading to inconsistent dosing decisions. In the absence of a standardized optimization approach, we utilized the R package *optimx* [55], a flexible optimization wrapper offering access to multiple methods available in R. This allowed us to systematically test various algorithms during the MLE procedure.

We also investigated optimization strategies to address numerical instability during maximum likelihood estimation. In the absence of a standardized optimization approach, we utilized the R package *optimx* [56], a flexible optimization wrapper offering access to multiple methods available in R (e.g., “Nelder‐Mead,” “BFGS,” “CG,” “L‐BFGS‐B,” “nlm,” “nlminb”), which allowed us to systematically test various algorithms during the MLE procedure (see Supporting Information 19). While we explored both optimization strategies and starting value initialization (results not shown), algorithm performance varied across individual trials and no single algorithm fully resolved the fundamental challenges posed by sparse data. These findings highlight a critical limitation of the frequentist framework, which remains heavily dependent on appropriate dose labels and is especially sensitive to data configurations.

The proposed joint method proves to be more robust than traditional approaches, showing consistent performance regardless of the initial stage and dose label calibration. It achieves improved PCS results and fewer estimation issues overall, making it a promising tool in dose‐finding studies. However, several extensions merit consideration. First, the chosen AR(1) dependence model assumes that the correlation between biomarker measurements decays exponentially with increasing time lag such that measurements far apart in time contribute relatively little shared information. While this offers parsimony, alternative models such as random effects specifications induce a constant within‐subject correlation regardless of the time lag—a structure that may be more realistic for biomarkers whose trajectories are largely driven by stable patient‐level characteristics. To assess the robustness of the proposed methods to this assumption, we evaluated performance under a random effects data generation mechanism in Supporting Information 20, where the same general patterns were observed, suggesting that the conclusions of this work are not materially sensitive to the choice of dependence structure. Second, while the factorization‐based modeling framework offers computational advantages, alternative approaches—such as joint likelihood specifications based on latent variable [57]—may be able to capture complex dependencies between the toxicity, efficacy, and biomarker results and warrant exploration. Such latent variable specifications could potentially enable the use of more parsimonious one‐parameter models while maintaining adequate representation of the multivariate outcome structure. Third, beyond model extensions, while the current frequentist approach, adopting a Bayesian framework could be a more effective solution. Bayesian methods, with their ability to incorporate prior information and handle uncertainty more effectively, may provide additional stability and flexibility in real‐world clinical applications. Future work should therefore explore Bayesian extensions of both the Probit and joint methods to improve estimation accuracy and operational performance across diverse clinical scenarios.

## Funding

This project has received funding from Horizon 2020 research and innovation program under grant agreement No. 965397. This report is independent research supported by the National Institute for Health and Care Research (NIHR506292 and NIHR503042). The views expressed in this publication are those of the authors and not necessarily those of the NHS, the National Institute for Health and Care Research or the Department of Health and Social Care (DHCS). PM and TJ also received funding from the UK Medical Research Council (MC_UU_00002/14, MC_UU_00002/19, and MC_UU_00040/03). Infrastructure support is acknowledged from Cancer Research UK, the NIHR Cambridge Biomedical Research Centre (BRC‐1215‐20014) and Cambridge Experimental Cancer Medicine Centre.

## Conflicts of Interest

The authors declare no conflicts of interest.

## Supporting information




**Figure S1:** Flowchart of the two‐stage design **Figure S2:** The one‐parameter probit model with various values of $a_{0}$. Corresponding dose labels and the reference dose are illustrated. Reference dose with $\pi (x^{*})=(0.5,0.691,0.841,0.998,1)$ corresponding to $a_{0}=(0,0.5,1,3,5)$. **Figure S3:** Conditional variance under different choices of parameter $\rho_{b}$ and $\rho_{c}$ ($\sigma_{c}=1$). **Figure S4:** Positive definiteness of a variance–covariance matrix under different $\rho_{b}$ and $\rho_{c}$, 0 and 1 indicate positive definiteness and nonpositive definiteness ($\sigma_{c}=1$). **Figure S5:** Illustration of the hypothetical trial without the anchoring problem after implementing an alternative initial stage: Initial2. Stage 1 includes four cohorts and Stage 2 applies the Probit method based on the two‐parameter probit model. **Figure S6:** PCS of the Empiric method under different scenarios with different initial stages. **Table S1:** Performance trade‐offs of the Empiric method under increasingly complex initial stages. **Table S2:** PCS results under all scenarios and correlation values with all methods implementing the initial stage Initial3; proportions of separation in parentheses. The Probit method is reproduced under each $\rho_{b}$ as the full version of Figure 7b, with minor variation across $\rho_{b}$ values reflecting simulation error only. **Table S3:** Proportion of early stopping for being overtoxic. All three methods were implemented with Initial3 as the initial stage. **Figure S7:** Calibrated model and dose labels. **Figure S8:** Illustration of the hypothetical trial without the anchoring problem after calibration for dose labels (Initial1). **Figure S9:** PCS results of the Probit method before (Probit‐PreCal) and after calibration (Probit‐PostCal), with the Empiric method included as a reference. All methods implemented the initial stage Initial1. **Figure S10:** PCS results across five scenarios ($S_{1}$‐$S_{5}$) and $\rho_{b}\in \{0,0.4,0.8\}$ with all three methods implementing the initial stage Initial3, under a reduced sample size of 10 cohorts (half of that in Figure 7a). **Figure S11:** PCS results across five scenarios ($S_{1}$‐$S_{5}$) and $\rho_{b}\in \{0,0.4,0.8\}$ with all three methods implementing the initial stage Initial3, under a reduced sample size of 10 cohorts (half of that in Figure 11a). **Table S4:** Dose labels before and after calibration, with calibration applied using a method‐specific and $\rho_{b}$‐specific approach. **Table S5:** The proportion of separation and the proportion of equal estimated probability for the Probit method with and without the algorithm selection. The column “best” represents results with the algorithm selection. **Table S6:** The proportion of separation for the Joint2d method with and without the algorithm selection. The column “best” represents results with the algorithm selection. **Table S7:** The proportion of equal estimated probability for the Joint2d method with and without the algorithm selection. The column “best” represents results with the algorithm selection. **Figure S12:** PCS results across five scenarios ($S_{1}$‐$S_{5}$) and $\rho_{b}\in \{0,0.4,0.8\}$ with all three methods implementing the initial stage Initial1. **Figure S13:** PCS results across five scenarios ($S_{1}$‐$S_{5}$) and $\rho_{b}\in \{0,0.4,0.8\}$ with all three methods implementing the initial stage Initial3.

## Data Availability

The data that support the findings of this study are available from the corresponding author upon reasonable request.

## References

[1] A. Spreafico , A. R. Hansen , A. R. Abdul Razak , P. L. Bedard , and L. L. Siu , “The Future of Clinical Trial Design in Oncology,” Cancer Discovery 11, no. 4 (2021): 822–837. 10.1158/2159-8290.CD-20-1301 PMC8099154 33811119

[2] FDA , “FDA Promotes Clinical Trial Innovation,” (2024), https://www.fda.gov/news‐events/fda‐voices/fda‐promotes‐clinical‐trial‐innovation.

[3] FDA , “Project Optimus,” (2024), https://www.fda.gov/about‐fda/oncology‐center‐excellence/project‐optimus.

[4] Group BDW , A. J. Atkinson, Jr. , W. A. Colburn , et al., “Biomarkers and Surrogate Endpoints: Preferred Definitions and Conceptual Framework,” Clinical Pharmacology & Therapeutics 69, no. 3 (2001): 89–95. 10.1067/mcp.2001.113989 11240971

[5] E. Kilgour , D. G. Rothwell , G. Brady , and C. Dive , “Liquid Biopsy‐Based Biomarkers of Treatment Response and Resistance,” Cancer Cell 37, no. 4 (2020): 485–495. 10.1016/j.ccell.2020.03.012 32289272

[6] FDA‐NIH Biomarker Working Group , “BEST (Biomarkers, EndpointS, and Other Tools) Resource,” Food and Drug Administration (US), (2016). 27010052

[7] R. M. Califf , “Biomarker Definitions and Their Applications,” Experimental Biology and Medicine 243, no. 3 (2018): 213–221. 10.1177/1535370217750088 PMC5813875 29405771

[8] E. Rivera , M. Haim Erder , M. Fridman , D. Frye , and G. N. Hortobagyi , “First‐Cycle Absolute Neutrophil Count Can Be Used to Improve Chemotherapy‐Dose Delivery and Reduce the Risk of Febrile Neutropenia in Patients Receiving Adjuvant Therapy: A Validation Study,” Breast Cancer Research 5, no. 5 (2003): R114–R120. 10.1186/bcr618 PMC314422 12927041

[9] M. R. McGill , “The Past and Present of Serum Aminotransferases and the Future of Liver Injury Biomarkers,” EXCLI Journal 15 (2016): 817–828. 10.17179/excli2016-800 PMC5318690 28337112

[10] Q. Hu , A. Hada , and L. Han , “Platelet Count as a Biomarker for Monitoring Treatment Response and Disease Recurrence in Recurrent Epithelial Ovarian Cancer,” Journal of Ovarian Research 13, no. 1 (2020): 78, Epub 2020/07/20, PMID: 32682445, 10.1186/s13048-020-00682-z. PMC7368983 32682445

[11] R. Ferrara , C. Zemmour , T. Reichert , et al., “Predictive Factors for Persistent Thrombocytopenia After Peptide Receptor Radioligand Therapy in Enteropancreatic Neuroendocrine Tumors,” Frontiers in Endocrinology 16 (2025): 1568243. 10.3389/fendo.2025.1568243 PMC12119268 40444233

[12] J. C. Wan , C. Massie , J. Garcia‐Corbacho , et al., “Liquid Biopsies Come of Age: Towards Implementation of Circulating Tumour DNA,” Nature Reviews Cancer 17, no. 4 (2017): 223–238. 10.1038/nrc.2017.7 28233803

[13] L. R. Yates , M. Gerstung , S. Knappskog , et al., “Subclonal Diversification of Primary Breast Cancer Revealed by Multiregion Sequencing,” Nature Medicine 21, no. 7 (2015): 751–759. 10.1038/nm.3886 PMC4500826 26099045

[14] S. P. Shah , R. D. Morin , J. Khattra , et al., “Mutational Evolution in a Lobular Breast Tumour Profiled at Single Nucleotide Resolution,” Nature 461, no. 7265 (2009): 809–813. 10.1038/nature08489 19812674

[15] H. Satam , K. Joshi , U. Mangrolia , et al., “Next‐Generation Sequencing Technology: Current Trends and Advancements,” Biology 12, no. 7 (2023): 997. 10.3390/biology12070997 PMC10376292 37508427

[16] X. Chen , P. Mozgunov , R. D. Baird , and T. Jaki , “Using Circulating Tumor DNA as a Novel Biomarker of Efficacy for Dose‐Finding Designs in Oncology,” Statistical Methods in Medical Research 34, no. 8 (2025): 09622802251350457. 10.1177/09622802251350457 PMC12365363 40590061

[17] T. Jaki , S. Clive , and C. J. Weir , “Principles of Dose Finding Studies in Cancer: A Comparison of Trial Designs,” Cancer Chemotherapy and Pharmacology 71 (2013): 1107–1114. 10.1007/s00280-012-2059-8 PMC3636432 23299793

[18] A. Teixeira‐Pinto and J. Harezlak , “Factorization and Latent Variable Models for Joint Analysis of Binary and Continuous Outcomes,” in Analysis of Mixed Data: Methods & Applications, vol. 81 (CRC Press, Taylor & Francis Group, 2013), 91.

[19] A. Teixeira‐Pinto and S. L. T. Normand , “Correlated Bivariate Continuous and Binary Outcomes: Issues and Applications,” Statistics in Medicine 28, no. 13 (2009): 1753–1773. 10.1002/sim.3588 PMC2818753 19358234

[20] P. J. Catalano and L. M. Ryan , “Bivariate Latent Variable Models for Clustered Discrete and Continuous Outcomes,” Journal of the American Statistical Association 87, no. 419 (1992): 651–658.

[21] D. R. Cox and N. Wermuth , “Response Models for Mixed Binary and Quantitative Variables,” Biometrika 79, no. 3 (1992): 441–461.

[22] G. M. Fitzmaurice and N. M. Laird , “Regression Models for a Bivariate Discrete and Continuous Outcome With Clustering,” Journal of the American Statistical Association 90, no. 431 (1995): 845–852.

[23] M. D. Sammel , L. M. Ryan , and J. M. Legler , “Latent Variable Models for Mixed Discrete and Continuous Outcomes,” Journal of the Royal Statistical Society Series B: Statistical Methodology 59, no. 3 (1997): 667–678.

[24] D. B. Dunson , “Bayesian Latent Variable Models for Clustered Mixed Outcomes,” Journal of the Royal Statistical Society: Series B (Statistical Methodology) 62, no. 2 (2000): 355–366.

[25] B. N. Bekele and Y. Shen , “A Bayesian Approach to Jointly Modeling Toxicity and Biomarker Expression in a Phase I/II Dose‐Finding Trial,” Biometrics 61, no. 2 (2005): 343–354. 10.1111/j.1541-0420.2005.00314.x 16011680

[26] J. M. Wason and S. R. Seaman , “Using Continuous Data on Tumour Measurements to Improve Inference in Phase II Cancer Studies,” Statistics in Medicine 32, no. 26 (2013): 4639–4650. 10.1002/sim.5867 PMC4282550 23776143

[27] J. M. Wason and S. R. Seaman , “A Latent Variable Model for Improving Inference in Trials Assessing the Effect of Dose on Toxicity and Composite Efficacy Endpoints,” Statistical Methods in Medical Research 29, no. 1 (2020): 230–242. 10.1177/0962280219831038 PMC6986906 30799777

[28] J. O'Quigley , M. Pepe , and L. Fisher , “Continual Reassessment Method: A Practical Design for Phase 1 Clinical Trials in Cancer,” Biometrics 46 (1990): 33–48. 2350571

[29] J. O'Quigley and L. Z. Shen , “Continual Reassessment Method: A Likelihood Approach,” Biometrics 52 (1996): 673–684. 8672707

[30] L. Z. Shen and J. O'QUIGLEY , “Consistency of Continual Reassessment Method Under Model Misspecification,” Biometrika 83, no. 2 (1996): 395–405.

[31] Y. K. Cheung and R. Chappell , “A Simple Technique to Evaluate Model Sensitivity in the Continual Reassessment Method,” Biometrics 58, no. 3 (2002): 671–674. 10.1111/j.0006-341x.2002.00671.x 12230003

[32] J. O'Quigley , “Theoretical Study of the Continual Reassessment Method,” Journal of Statistical Planning and Inference 136, no. 6 (2006): 1765–1780.

[33] X. Paoletti and A. Kramar , “A Comparison of Model Choices for the Continual Reassessment Method in Phase I Cancer Trials,” Statistics in Medicine 28, no. 24 (2009): 3012–3028. 10.1002/sim.3682 19672839

[34] Y. K. Cheung , Dose Finding by the Continual Reassessment Method (CRC Press, 2011).

[35] E. Garrett‐Mayer , “The Continual Reassessment Method for Dose‐Finding Studies: A Tutorial,” Clinical Trials 3, no. 1 (2006): 57–71. 10.1191/1740774506cn134oa 16539090

[36] G. M. Wheeler , A. P. Mander , A. Bedding , et al., “How to Design a Dose‐Finding Study Using the Continual Reassessment Method,” BMC Medical Research Methodology 19 (2019): 1–15. 10.1186/s12874-018-0638-z PMC6339349 30658575

[37] B. E. Storer , “Design and Analysis of Phase I Clinical Trials,” Biometrics 45 (1989): 925–937. 2790129

[38] M. J. Silvapulle , “On the Existence of Maximum Likelihood Estimators for the Binomial Response Models,” Journal of the Royal Statistical Society. Series B, Statistical Methodology 43 (1981): 310–313.

[39] A. Albert and J. A. Anderson , “On the Existence of Maximum Likelihood Estimates in Logistic Regression Models,” Biometrika 71, no. 1 (1984): 1–10.

[40] E. Lesaffre and H. Kaufmann , “Existence and Uniqueness of the Maximum Likelihood Estimator for a Multivariate Probit Model,” Journal of the American Statistical Association 87, no. 419 (1992): 805–811.

[41] P. Gresele , G. Guglielmini , M. Del Pinto , et al., “Low Platelet Count at Admission Has an Adverse Impact on Outcome in Patients With Acute Coronary Syndromes: From the START Antiplatelet Registry,” Scientific Reports 14, no. 1 (2024): 14516. 10.1038/s41598-024-64113-5 PMC11196263 38914608

[42] A. R. De Leon and K. C. Chough , Analysis of Mixed Data: Methods & Applications (CRC Press, 2013).

[43] D. Patnaik and N. Sharma , “Selection Problems for Application of Probit. Tobit. Logit and Maximum Likelihood Estimation: A Methodological Issue,” International Journal of Computational Engineering Research 3, no. 7 (2013): 13–29.

[44] C. Choe , S. Jung , and R. L. Oaxaca , “Identification and Decompositions in Probit and Logit Models,” Empirical Economics 59 (2020): 1479–1492.

[45] B. Bolker and R Development Core Team , “bbmle: Tools for General Maximum Likelihood Estimation,” R Package Version 1.0.25.1, (2023), https://CRAN.R‐project.org/package=bbmle.

[46] R Core Team , “R: A Language and Environment for Statistical Computing,” R Foundation for Statistical Computing, Vienna, Austria, (2023), https://www.R‐project.org/.

[47] D. Firth , “Bias Reduction of Maximum Likelihood Estimates,” Biometrika 80, no. 1 (1993): 27–38.

[48] H. Jeffreys , “An Invariant Form for the Prior Probability in Estimation Problems,” Proceedings of the Royal Society of London. Series A, Mathematical and Physical Sciences 186, no. 1007 (1946): 453–461. 10.1098/rspa.1946.0056 20998741

[49] M. A. Altzerinakou and X. Paoletti , “Change‐Point Joint Model for Identification of Plateau of Activity in Early Phase Trials,” Statistics in Medicine 40, no. 9 (2021): 2113–2138. 10.1002/sim.8889 33561898

[50] S. Chevret , “The Continual Reassessment Method in Cancer Phase I Clinical Trials: A Simulation Study,” Statistics in Medicine 12, no. 12 (1993): 1093–1108. 10.1002/sim.4780121201 8210815

[51] E. Demidenko , “Computational Aspects of Probit Model,” Mathematical Communications 6, no. 2 (2001): 233–247.

[52] S. M. Lee and Y. K. Cheung , “Model Calibration in the Continual Reassessment Method,” Clinical Trials 6, no. 3 (2009): 227–238. 10.1177/1740774509105076 PMC2884971 19528132

[53] J. O'quigley , X. Paoletti , and J. Maccario , “Non‐Parametric Optimal Design in Dose Finding Studies,” Biostatistics 3, no. 1 (2002): 51–56. 10.1093/biostatistics/3.1.51 12933623

[54] P. Mozgunov , T. Jaki , and X. Paoletti , “A Benchmark for Dose Finding Studies With Continuous Outcomes,” Biostatistics 21, no. 2 (2020): 189–201. 10.1093/biostatistics/kxy045 30165594

[55] J. C. Nash , “On Best Practice Optimization Methods in R,” Journal of Statistical Software 60, no. 2 (2014): 1–14.

[56] J. C. Nash and R. Varadhan , “Unifying Optimization Algorithms to Aid Software System Users: Optimx for R,” Journal of Statistical Software 43, no. 9 (2011): 1–14.

[57] C. McCulloch , “Joint Modelling of Mixed Outcome Types Using Latent Variables,” Statistical Methods in Medical Research 17, no. 1 (2008): 53–73. 10.1177/0962280207081240 17855745

