# Supplementary material for: Using Joint Models in Phase I Dose‐Finding Designs in Oncology: Considerations for Frequentist Approaches
Source: Stat Med. 2026 Oct 13;45(23-24):e70758. doi: 10.1002/sim.70758 (PMC13659646; doi:10.1002/sim.70758)
Supplement: Supplementary file 1 [file SIM-45-0-s001.pdf]

Supporting Information

7 | FLOWCHART OF THE TWO-STAGE DESIGN

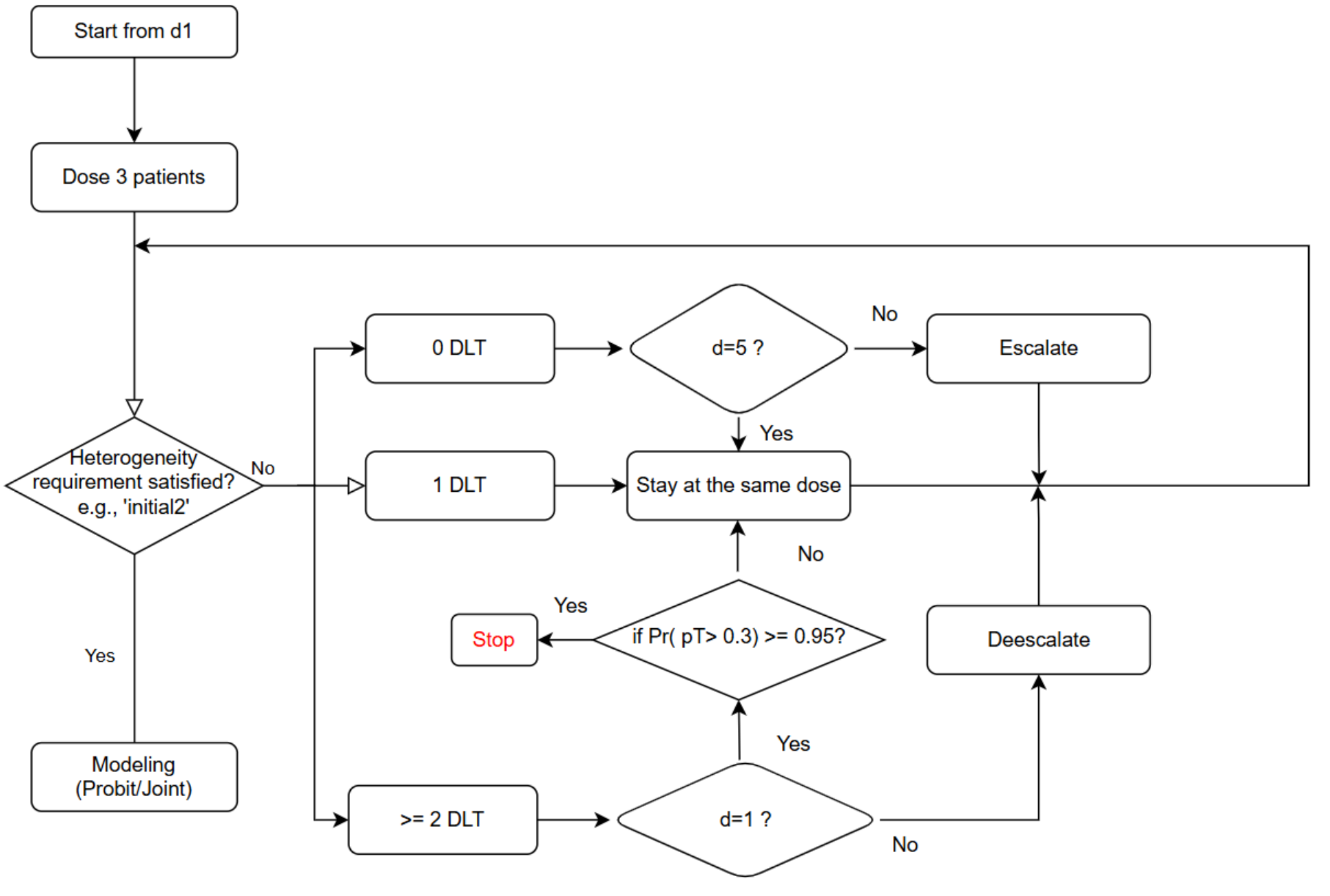

FIGURE 12 Flowchart of the two-stage design

8 | IMPACT OF THE INTERCEPT a_0 ON THE ONE-PARAMETER PROBIT MODEL

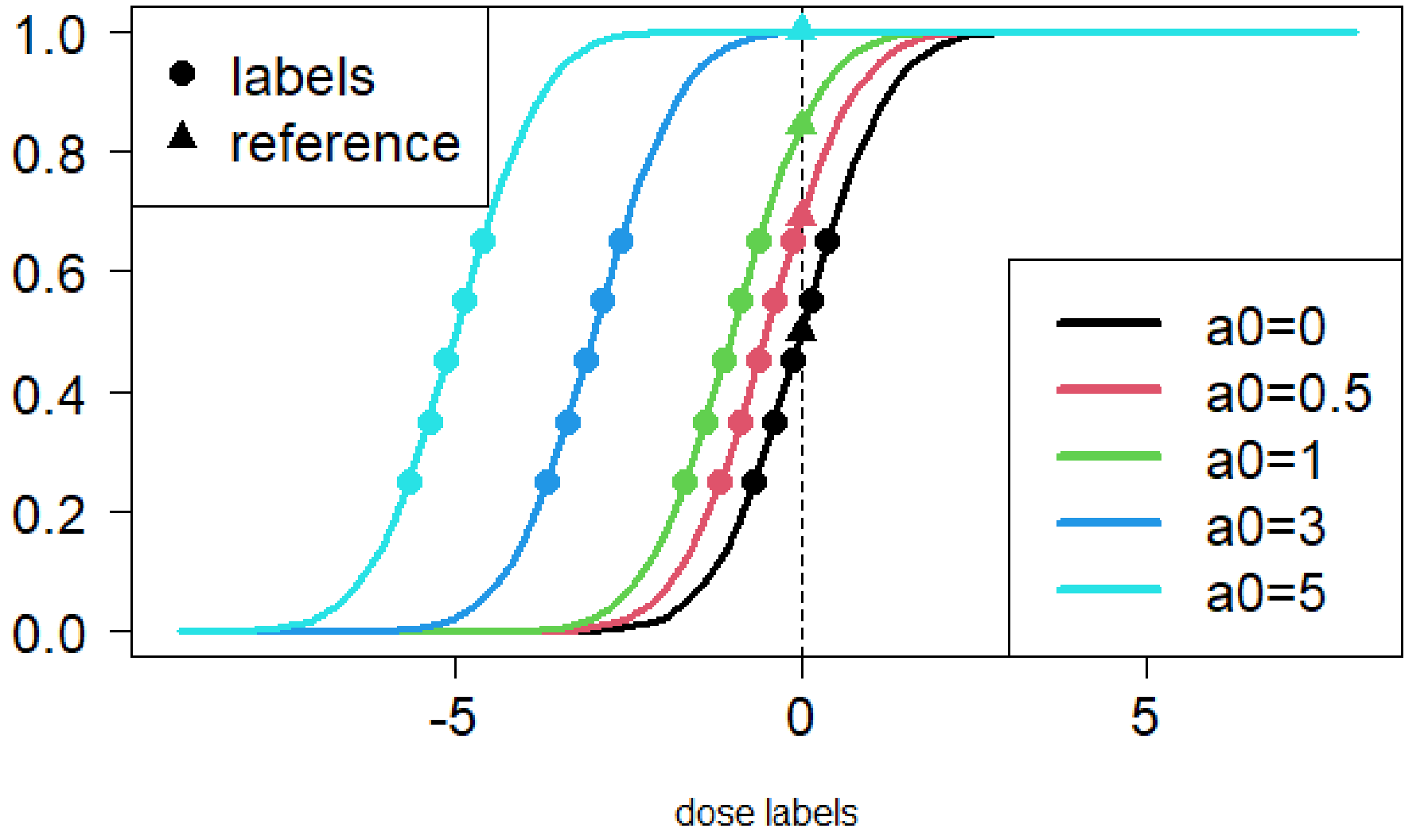

FIGURE 13 The one-parameter probit model with various values of a_0. Corresponding dose labels and the reference dose are illustrated. Reference dose with $\pi(x^*) = (0.5, 0.691, 0.841, 0.998, 1)$ corresponding to $a_0 = (0, 0.5, 1, 3, 5)$.

9 | DERIVATION FOR THE JOINT9D METHOD

The Joint9d method models the joint probability for individual i at administered dose x_j of the binary variable Y_{bi} and all $Y_{ci,t}$, $t \in (1, \dots, 8)$. For the correlation ρ_b between Y_b and $Y_{c,8}$, we assume $\rho_b \geq 0$. To account for negative correlations between outcomes in higher dimensions, the sign of the correlation is introduced explicitly, avoiding ambiguity in interpretation.

Following the framework of factorization above, we are now interested in the marginal model for $\mathbf{Y}_{ci}$, which is multivariate normally distributed, and a conditional model for Y_{bi} given $\mathbf{Y}_{ci}$. The derived conditional distribution is:

$$E(Y_{bi}|\mathbf{Y_{ci}} = \mathbf{y_{ci}}) = \Phi\left(\mu_{bi} + \tau(\mathbf{Y_{ci}} - \mu_{\mathbf{ci}})\right) \text{ and } \mathbf{Y_{ci}} = \mu_{\mathbf{ci}} + \epsilon_{\mathbf{ci}}. \tag{11}$$

where $\mu_{bi} = \beta_{b0} + \exp(\beta_{b1}) \cdot x_j$, $\mu_{ci,t} = \beta_{c0} + \beta_{c1} \cdot x_j + a_t \cdot t$ ($t \in 1, \dots, 8$), and $\epsilon_{\mathbf{ci}} \sim \mathbf{N}_8\,(0, \mathbf{\Sigma_c})$. For biomarker values, β_{c0} is the intercept, β_{c1} indicates the dose-response relationship, and a_t captures the change of mean values μ_t with time. Corresponding parameters are expressed as $\boldsymbol{\theta} = (\beta_{b0}, \beta_{b1}, \beta_{c0}, \beta_{c1}, a_t, \tau, \sigma_c)$.

$$\begin{pmatrix} Y_{ci,1} \\ Y_{ci,2} \\ Y_{ci,3} \\ Y_{ci,4} \\ Y_{ci,5} \\ Y_{ci,6} \\ Y_{ci,7} \\ Y_{ci,8} \\ Y^*_{bi} \end{pmatrix} \sim \mathcal{N}\left(\begin{pmatrix} \mu_{ci,1} \\ \mu_{ci,2} \\ \mu_{ci,3} \\ \mu_{ci,4} \\ \mu_{ci,5} \\ \mu_{ci,6} \\ \mu_{ci,7} \\ \mu_{ci,8} \\ \mu^*_{bi} \end{pmatrix}, \begin{pmatrix} \sigma_c^2 & \rho_c\sigma_c^2 & \rho_c^2\sigma_c^2 & \rho_c^3\sigma_c^2 & \rho_c^4\sigma_c^2 & \rho_c^5\sigma_c^2 & \rho_c^6\sigma_c^2 & \rho_c^7\sigma_c^2 & -\rho_b^8\sigma_c \\ \rho_c\sigma_c^2 & \sigma_c^2 & \rho_c\sigma_c^2 & \rho_c^2\sigma_c^2 & \rho_c^3\sigma_c^2 & \rho_c^4\sigma_c^2 & \rho_c^5\sigma_c^2 & \rho_c^6\sigma_c^2 & -\rho_b^7\sigma_c \\ \rho_c^2\sigma_c^2 & \rho_c\sigma_c^2 & \sigma_c^2 & \rho_c\sigma_c^2 & \rho_c^2\sigma_c^2 & \rho_c^3\sigma_c^2 & \rho_c^4\sigma_c^2 & \rho_c^5\sigma_c^2 & -\rho_b^6\sigma_c \\ \rho_c^3\sigma_c^2 & \rho_c^2\sigma_c^2 & \rho_c\sigma_c^2 & \sigma_c^2 & \rho_c\sigma_c^2 & \rho_c^2\sigma_c^2 & \rho_c^3\sigma_c^2 & \rho_c^4\sigma_c^2 & -\rho_b^5\sigma_c \\ \rho_c^4\sigma_c^2 & \rho_c^3\sigma_c^2 & \rho_c^2\sigma_c^2 & \rho_c\sigma_c^2 & \sigma_c^2 & \rho_c\sigma_c^2 & \rho_c^2\sigma_c^2 & \rho_c^3\sigma_c^2 & -\rho_b^4\sigma_c \\ \rho_c^5\sigma_c^2 & \rho_c^4\sigma_c^2 & \rho_c^3\sigma_c^2 & \rho_c^2\sigma_c^2 & \rho_c\sigma_c^2 & \sigma_c^2 & \rho_c\sigma_c^2 & \rho_c^2\sigma_c^2 & -\rho_b^3\sigma_c \\ \rho_c^6\sigma_c^2 & \rho_c^5\sigma_c^2 & \rho_c^4\sigma_c^2 & \rho_c^3\sigma_c^2 & \rho_c^2\sigma_c^2 & \rho_c\sigma_c^2 & \sigma_c^2 & \rho_c\sigma_c^2 & -\rho_b^2\sigma_c \\ \rho_c^7\sigma_c^2 & \rho_c^6\sigma_c^2 & \rho_c^5\sigma_c^2 & \rho_c^4\sigma_c^2 & \rho_c^3\sigma_c^2 & \rho_c^2\sigma_c^2 & \rho_c\sigma_c^2 & \sigma_c^2 & -\rho_b\sigma_c \\ -\rho_b^8\sigma_c & -\rho_b^7\sigma_c & -\rho_b^6\sigma_c & -\rho_b^5\sigma_c & -\rho_b^4\sigma_c & -\rho_b^3\sigma_c & -\rho_b^2\sigma_c & -\rho_b\sigma_c & 1 \end{pmatrix}\right) \tag{12}$$

The conditional distribution of Y_{bi} given Y_{ci} is

$$E(Y_{bi}|\mathbf{Y_{ci}} = (\mu_{bi} + \begin{bmatrix} -\frac{(-\rho_b^8+\rho_b^7\rho_c)}{\sigma_c(\rho_c^2-1)} \\ -\frac{(-\rho_b^7-\rho_b^7\rho^2+\rho_b^8\rho+\rho_b^6\rho_c)}{\sigma_c(\rho_c^2-1)} \\ -\frac{(-\rho_b^6-\rho_b^6\rho_c^2+\rho_b^7\rho_c+\rho_b^5\rho_c)}{\sigma_c(\rho_c^2-1)} \\ -\frac{(-\rho_b^5-\rho_b^5\rho_c^2+\rho_b^6\rho_c+\rho_b^4\rho_c-\rho_b^5-\rho_b^5\rho_c^2+\rho_b^6\rho_c+\rho_b^4\rho_c)}{\sigma_c(\rho_c^2-1)} \\ -\frac{(-\rho_b^4-\rho_b^4\rho_c^2+\rho_b^5\rho_c+\rho_b^3\rho_c)}{\sigma_c(\rho_c^2-1)} \\ -\frac{(-\rho_b^3-\rho_b^3\rho_c^2+\rho_b^4\rho_c+\rho_b^2\rho_c)}{\sigma_c(\rho_c^2-1)} \\ -\frac{(-\rho_b^2-\rho_b^2\rho_c^2+\rho_b^3\rho_c+\rho_b\rho_c)}{\sigma_c(\rho_c^2-1)} \\ -\frac{(-\rho_b+\rho_b^2\rho_c)}{\sigma_c(\rho_c^2-1)} \end{bmatrix}^T \begin{bmatrix} (y_{ci,1}-\mu_{ci,1}) \\ (y_{ci,2}-\mu_{ci,2}) \\ (y_{ci,3}-\mu_{ci,3}) \\ (y_{ci,4}-\mu_{ci,4}) \\ (y_{ci,5}-\mu_{ci,5}) \\ (y_{ci,6}-\mu_{ci,6}) \\ (y_{ci,7}-\mu_{ci,7}) \\ (y_{ci,8}-\mu_{ci,8}) \end{bmatrix}, \Sigma_{b*|c}), \tag{13}$$

where $\mu_{bi} = \beta_{b0} + \exp(\beta_{b1}) \cdot x_j$ and

$$\Sigma_{b*|c} = (1 - (-\rho_b^{16} - \rho_b^{14} - \rho_b^{12} - \rho_b^{10} - \rho_b^{8} - \rho_b^{6} - \rho_b^{4} - \rho_b^{2} - \rho_b^{14}\rho_c^2 - \rho_b^{12}\rho_c^2 - \rho_b^{10}\rho_c^2 - \rho_b^{8}\rho_c^2 - \rho_b^{6}\rho_c^2 - \rho_b^{4}\rho_c^2 + 2\rho_b^{15}\rho_c + 2\rho_b^{13}\rho_c + 2\rho_b^{11}\rho_c + 2\rho_b^{9}\rho_c + 2\rho_b^{7}\rho_c + 2\rho_b^{5}\rho_c + 2\rho_b^{3}\rho_c) \quad (14)$$

The reparameterization is derived from the marginal effects outlined in Equation 13 and the conditional variance specified in Equation 14. That is $\beta_{b0}^* = \beta_{b0} \cdot \sqrt{\Sigma_{b*|c}}$ and $\beta_{b1}^* = \exp(\beta_{b1}) \cdot \sqrt{\Sigma_{b*|c}}$.

10 | CONSTRAINTS FOR CHOICES OF PARAMETERS

Results for $\sigma_c = 1$ are presented, as its value influences the conditional variance and the positive definiteness of the covariance matrix.

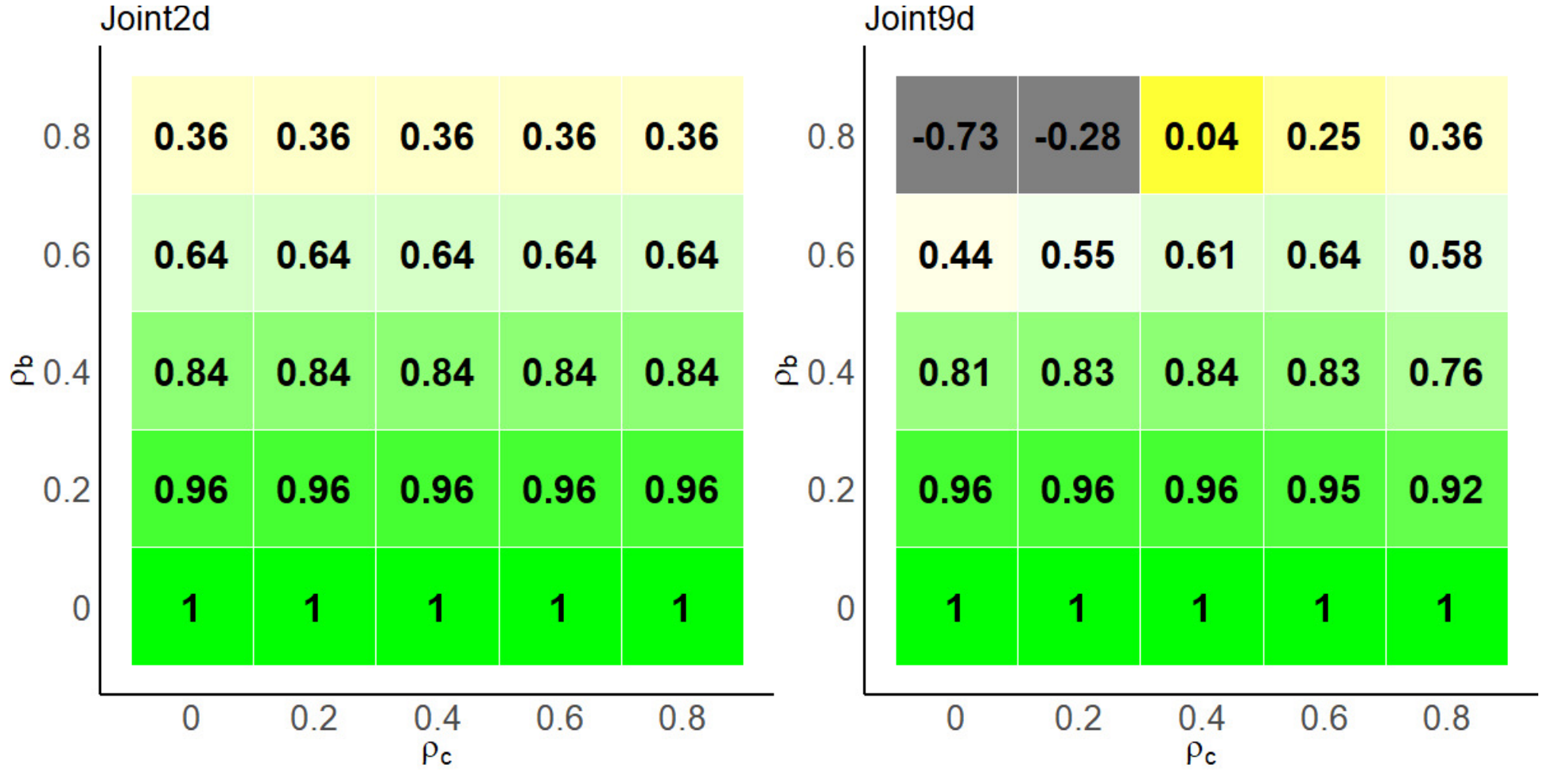

FIGURE 14 Conditional variance under different choices of parameter ρ_b and ρ_c ($\sigma_c = 1$).

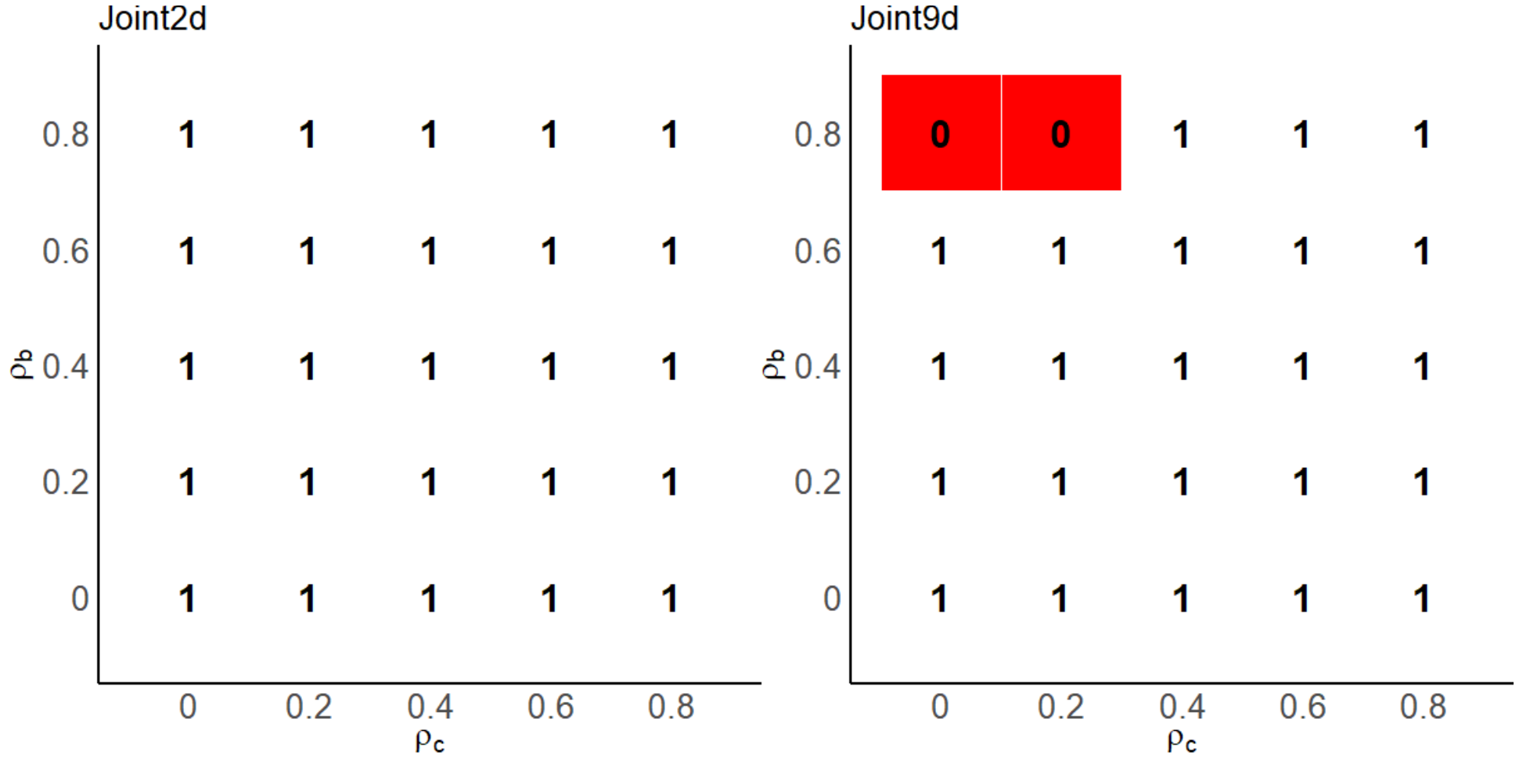

FIGURE 15 Positive definiteness of a variance-covariance matrix under different ρ_b and ρ_c, 0 and 1 indicate positive definiteness and non-positive definiteness ($\sigma_c = 1$).

11 | NO ANCHORING PROBLEM WITH INITIAL2

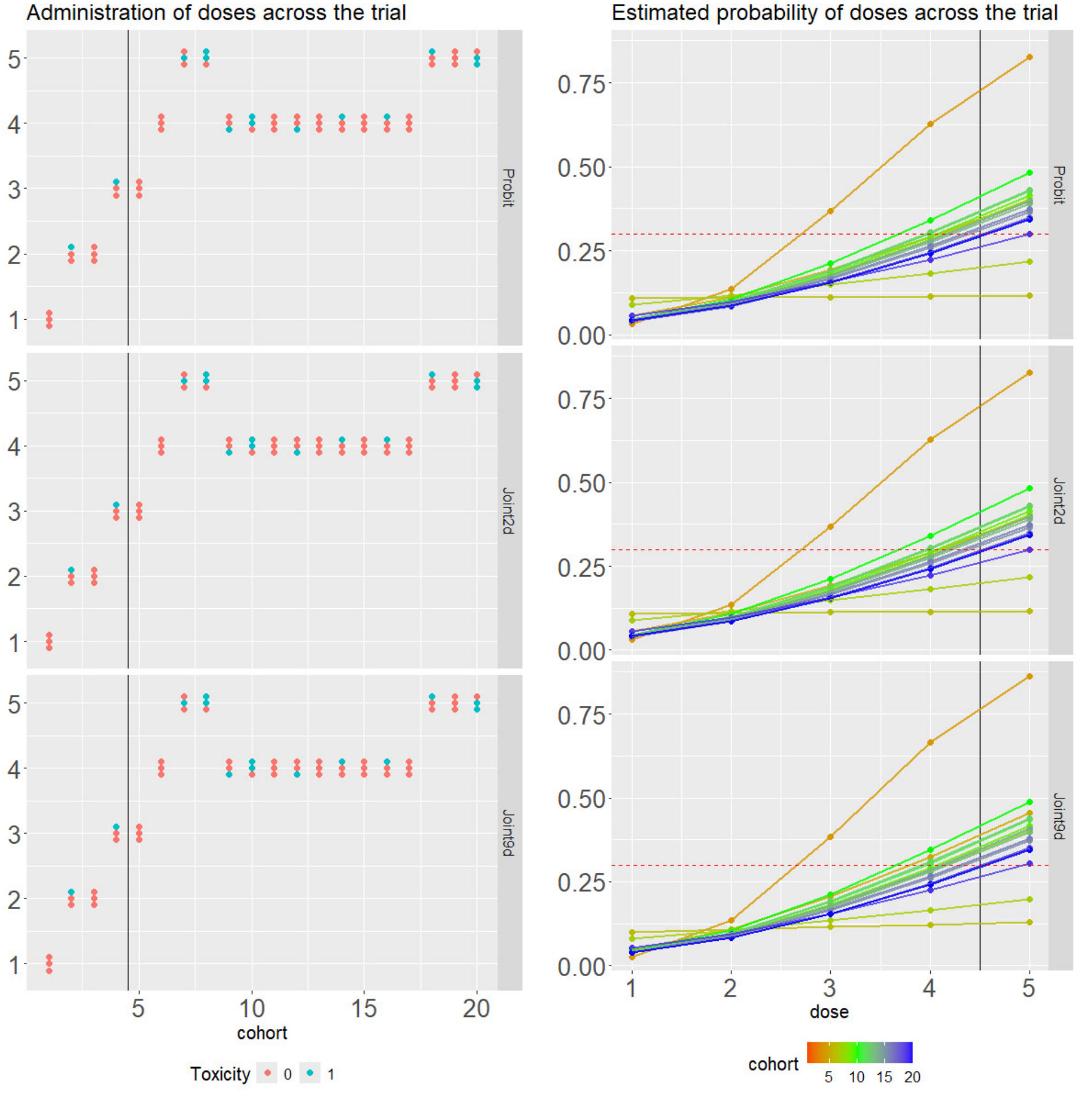

FIGURE 16 Illustration of the hypothetical trial without the anchoring problem after implementing an alternative initial stage: Initial2. Stage 1 includes four cohorts and Stage 2 applies the Probit method based on the two-parameter probit model.

12 | PCS OF THE EMPIRIC METHOD UNDER DIFFERENT INITIAL STAGES

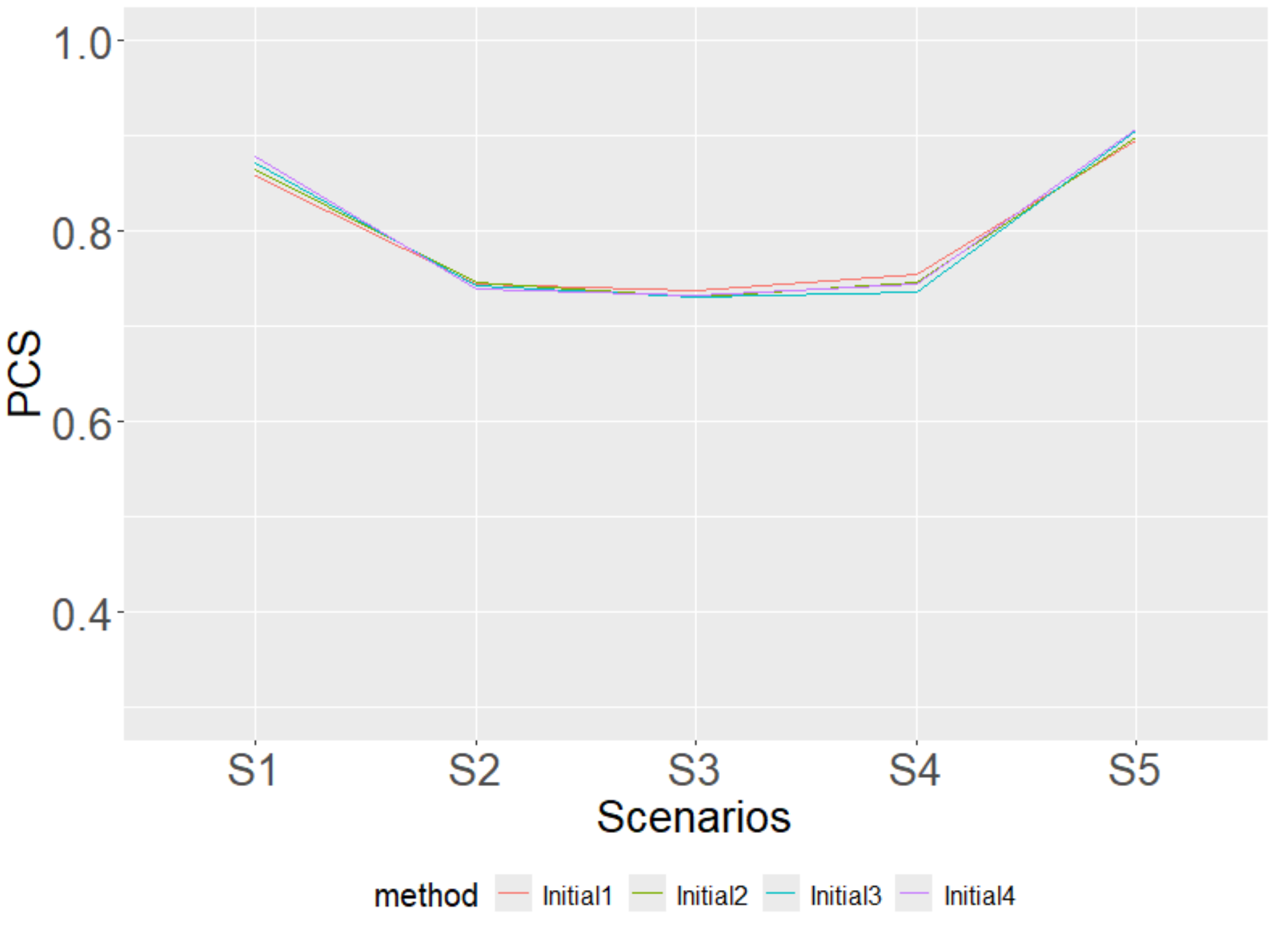

FIGURE 17 PCS of the Empiric method under different scenarios with different initial stages.

	Initial1	Initial2	Initial3	Initial4
S_1	0	0	0	0
S_2	0	0	0	0
S_3	0	0	0	0
S_4	0	0	0	0
S_5	0	0	0	0

(a) Proportion of the separation

	Initial1	Initial2	Initial3	Initial4
S_1	1.40	2.42	3.12	3.95
S_2	1.86	3.01	4.05	5.06
S_3	2.29	3.72	4.89	6.00
S_4	3.00	4.58	5.87	7.01
S_5	3.52	5.44	7.00	8.44

(b) Average number of cohorts required in the initial stage

TABLE 9 Performance trade-offs of the Empiric method under increasingly complex initial stages.

13 | PCS RESULTS OF THREE METHODS ACROSS SCENARIOS

	$\rho_b = 0$			$\rho_b = 0.4$			$\rho_b = 0.8$		
	Probit	Joint2d	Joint9d	Probit	Joint2d	Joint9d	Probit	Joint2d	Joint9d
S1	0.76 (0.23)	0.76 (0.22)	0.77 (0.22)	0.74 (0.23)	0.75 (0.22)	0.77 (0.23)	0.76 (0.24)	0.80 (0.20)	0.85 (0.06)
S2	0.61 (0.43)	0.63 (0.40)	0.62 (0.40)	0.59 (0.42)	0.60 (0.38)	0.62 (0.34)	0.64 (0.41)	0.66 (0.29)	0.78 (0.04)
S3	0.58 (0.33)	0.59 (0.33)	0.58 (0.32)	0.58 (0.31)	0.60 (0.29)	0.62 (0.30)	0.56 (0.32)	0.65 (0.22)	0.78 (0.03)
S4	0.57 (0.46)	0.57 (0.46)	0.57 (0.44)	0.58 (0.45)	0.60 (0.40)	0.60 (0.40)	0.59 (0.47)	0.65 (0.29)	0.77 (0.07)
S5	0.70 (0.45)	0.71 (0.45)	0.70 (0.46)	0.72 (0.45)	0.74 (0.44)	0.74 (0.45)	0.73 (0.43)	0.81 (0.33)	0.88 (0.15)

TABLE 10 PCS results under all scenarios and correlation values with all methods implementing the initial stage Initial3; proportions of separation in parentheses. The Probit method is reproduced under each ρ_b as the full version of Figure 7b, with minor variation across ρ_b values reflecting simulation error only.

14 | PROPORTION OF EARLY STOPPING FOR BEING OVERTOXIC

ρ_b	0	0.4	0.8
S_1	0.073	0.090	0.072
S_2	0.004	0.012	0.005
S_3	0.001	0.001	0.002
S_4	0	0	0
S_5	0.001	0	0

TABLE 11 Proportion of early stopping for being overtoxic. All three methods were implemented with Initial3 as the initial stage.

15 | DOSE-TOXICITY CURVE AND CORRESPONDING DOSE LABELS BEFORE AND AFTER CALIBRATION

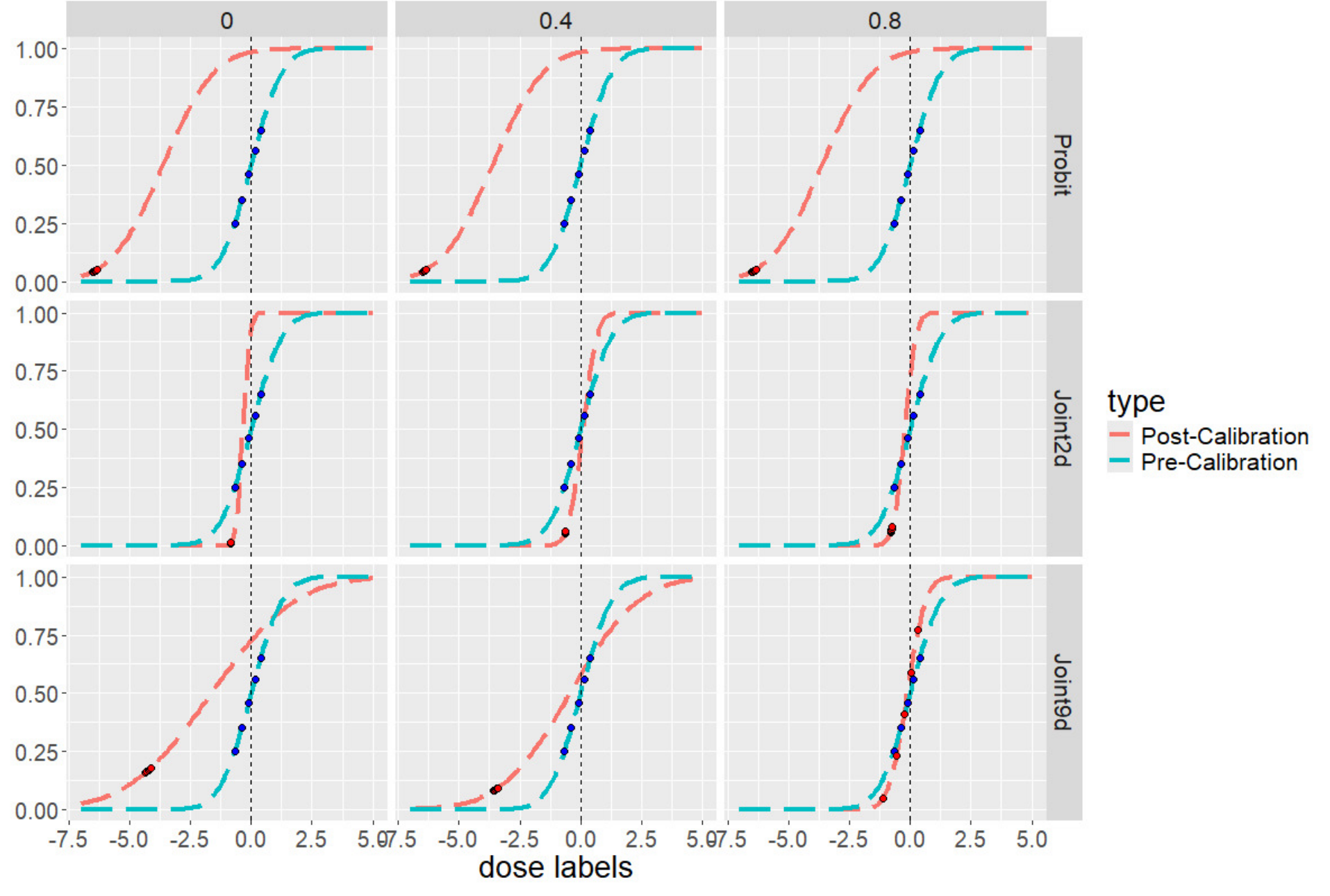

FIGURE 18 Calibrated model and dose labels.

16 | SIMULATION RESULT BY THE PROBIT METHOD WITH CALIBRATED LABLES WITH INITIAL1

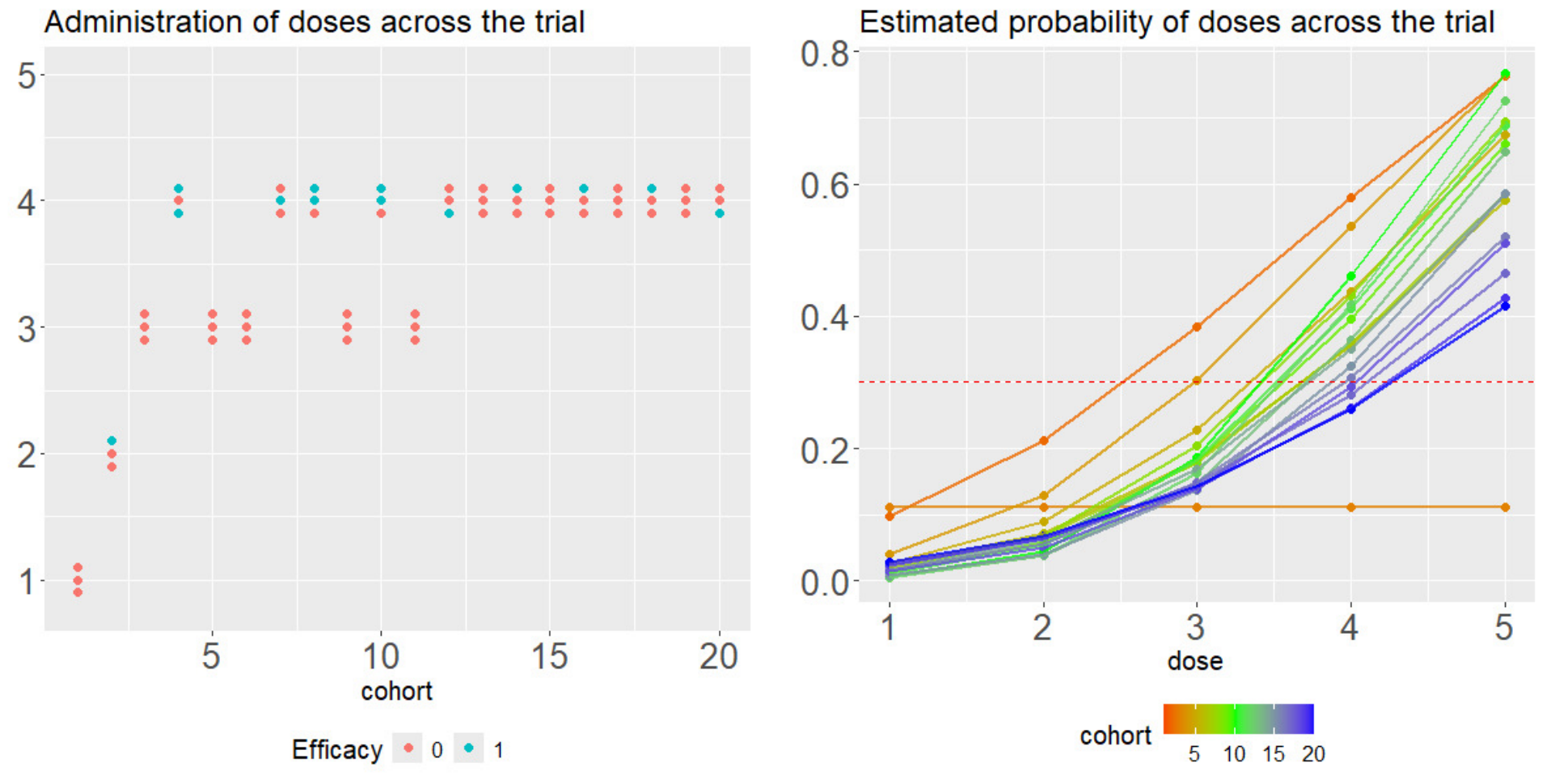

FIGURE 19 Illustration of the hypothetical trial without the anchoring problem after calibration for dose labels (Initial1).

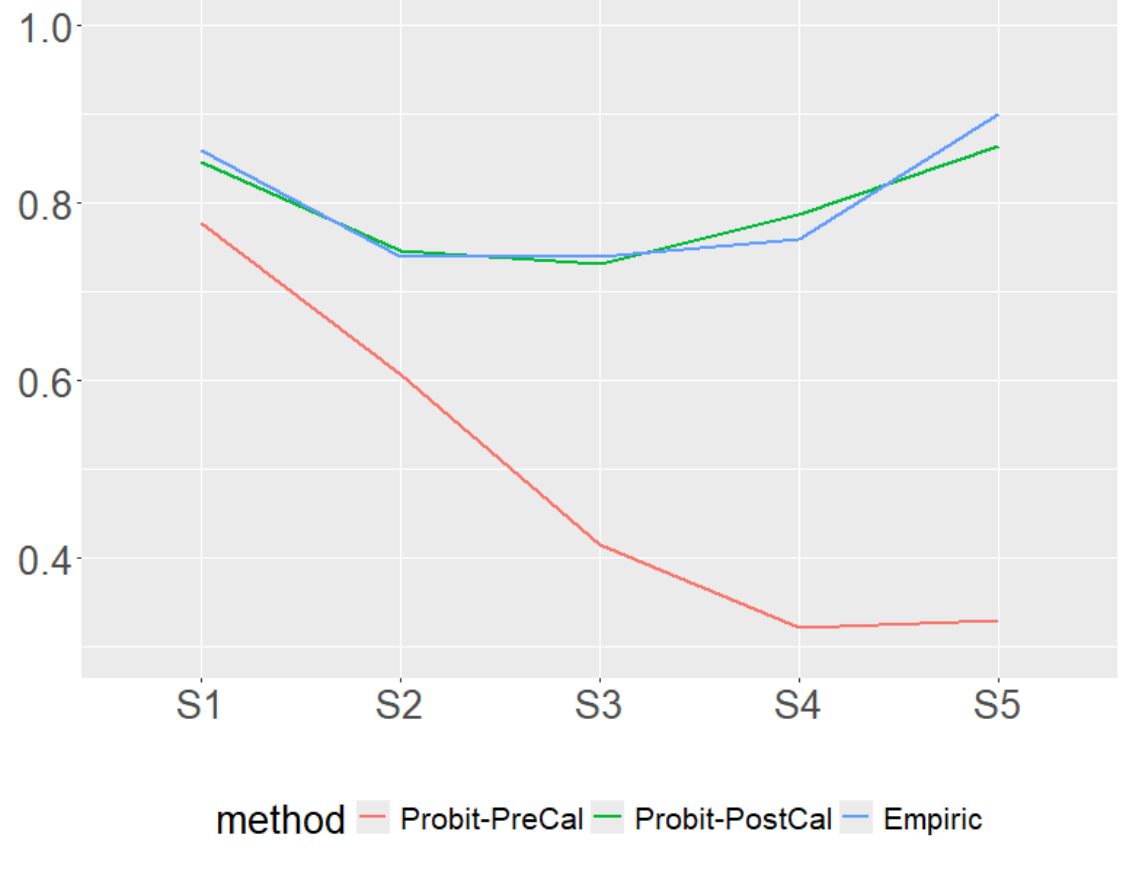

(a) PCS values before and after calibration; Empiric as reference.

	Empiric	Probit-PreCal	Probit-PostCal
S_1	0.86 (0)	0.78 (0.23)	0.85 (0)
S_2	0.74 (0)	0.61 (0.54)	0.75 (0)
S_3	0.74 (0)	0.42 (0.59)	0.73 (0)
S_4	0.76 (0)	0.32 (0.80)	0.79 (0)
S_5	0.90 (0)	0.33 (0.87)	0.86 (0)

(b) Numerical PCS values; proportions of separation in parentheses.

FIGURE 20 PCS results of the Probit method before (Probit-PreCal) and after calibration (Probit-PostCal), with the Empiric method included as a reference. All methods implemented the initial stage Initial1.

17 | SIMULATION RESULTS FOR SMALLER SAMPLE SIZES

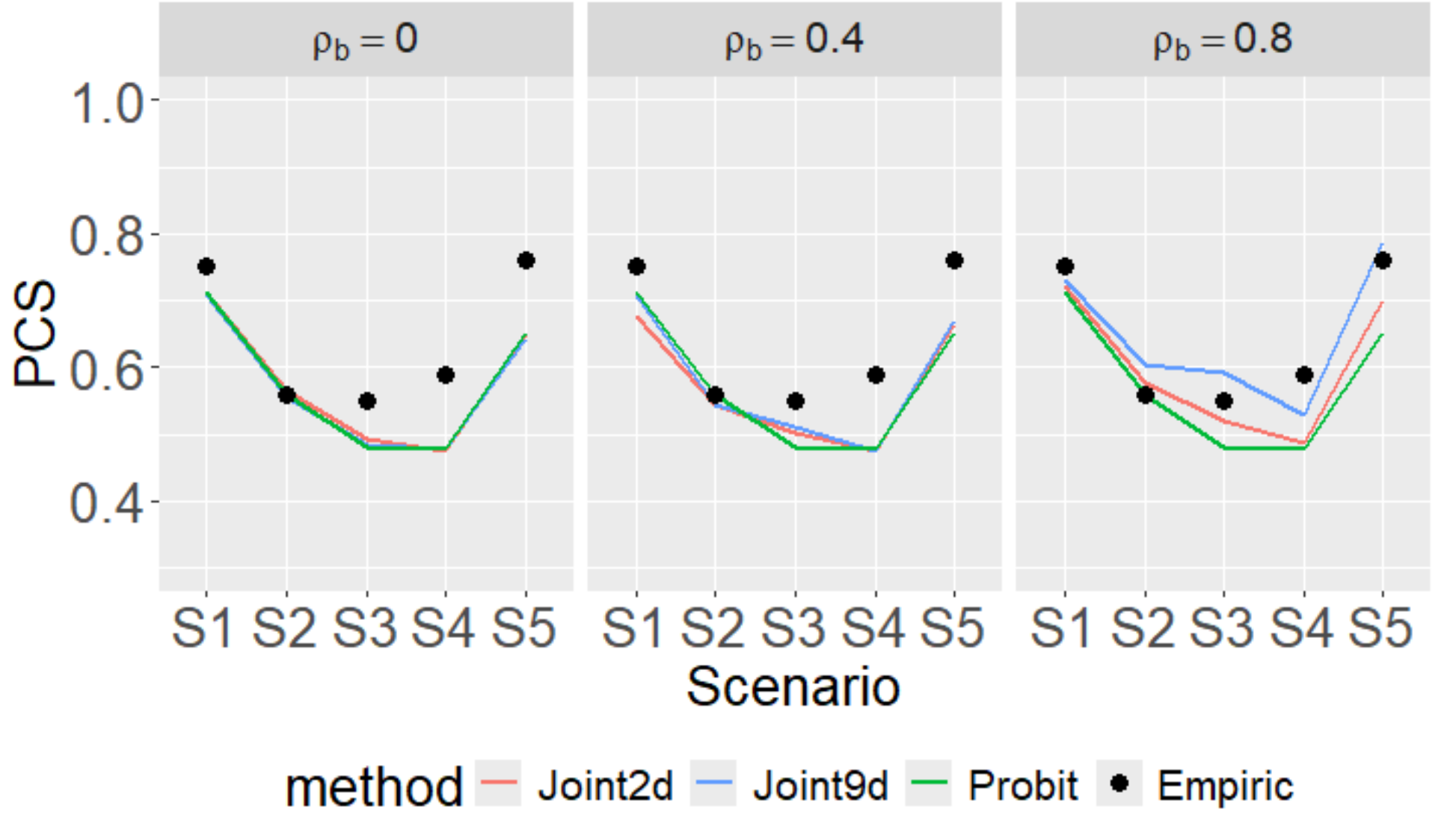

(a) PCS results across scenarios; Empiric method shown as points for reference ($S_1 - S_5$: 0.75, 0.56, 0.55, 0.59, 0.76).

	$\rho_b = 0$			$\rho_b = 0.4$		$\rho_b = 0.8$	
	Probit	Joint2d	Joint9d	Joint2d	Joint9d	Joint2d	Joint9d
S1	0.71 (0.23)	0.71 (0.22)	0.71 (0.22)	0.67 (0.22)	0.71 (0.23)	0.72 (0.20)	0.73 (0.06)
S2	0.56 (0.43)	0.57 (0.40)	0.56 (0.40)	0.54 (0.38)	0.54 (0.34)	0.58 (0.29)	0.60 (0.04)
S3	0.48 (0.33)	0.49 (0.33)	0.48 (0.32)	0.50 (0.29)	0.51 (0.30)	0.52 (0.22)	0.59 (0.03)
S4	0.48 (0.46)	0.48 (0.46)	0.48 (0.44)	0.48 (0.40)	0.48 (0.40)	0.49 (0.29)	0.53 (0.07)
S5	0.65 (0.45)	0.65 (0.45)	0.64 (0.46)	0.66 (0.44)	0.67 (0.45)	0.70 (0.33)	0.79 (0.15)

(b) PCS results; proportions of separation in parentheses. The Empiric method is excluded as separation does not arise under its one-parameter structure.

FIGURE 21 PCS results across five scenarios (S_1-S_5) and $\rho_b \in \{0, 0.4, 0.8\}$ with all three methods implementing the initial stage Initial3, under a reduced sample size of 10 cohorts (half of that in Figure 7a).

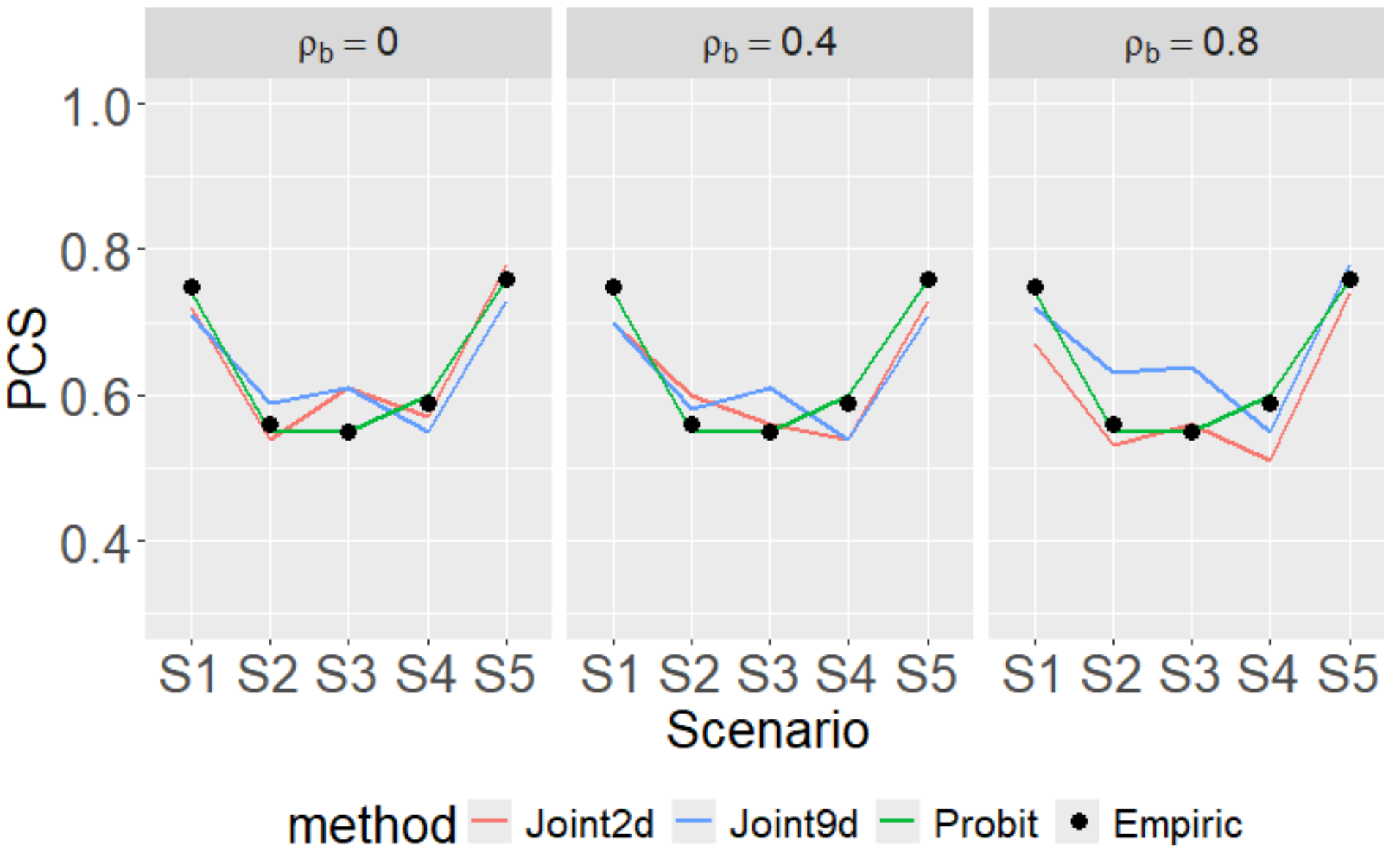

(a) PCS results across scenarios with calibrated dose labels; Empiric method shown as points for reference ($S_1 - S_5$: 0.75, 0.56, 0.55, 0.59, 0.76).

	$\rho_b = 0$			$\rho_b = 0.4$		$\rho_b = 0.8$	
	Probit	Joint2d	Joint9d	Joint2d	Joint9d	Joint2d	Joint9d
S1	0.74 (0)	0.72 (0)	0.71 (0)	0.70 (0)	0.70 (0)	0.67 (0)	0.72 (0.02)
S2	0.55 (0)	0.54 (0)	0.59 (0)	0.60 (0)	0.58 (0)	0.53 (0)	0.63 (0.02)
S3	0.55 (0)	0.61 (0)	0.61 (0)	0.56 (0)	0.61 (0)	0.56 (0)	0.64 (0.09)
S4	0.60 (0.05)	0.57 (0)	0.55 (0)	0.54 (0.02)	0.54 (0)	0.51 (0.04)	0.55 (0.16)
S5	0.76 (0.07)	0.78 (0)	0.73 (0.01)	0.73 (0.07)	0.71 (0.01)	0.74 (0.05)	0.78 (0.21)

(b) PCS results; proportions of separation in parentheses. The Empiric method is excluded as separation does not arise under its one-parameter structure.

FIGURE 22 PCS results across five scenarios (S_1-S_5) and $\rho_b \in \{0, 0.4, 0.8\}$ with all three methods implementing the initial stage Initial3, under a reduced sample size of 10 cohorts (half of that in Figure 11a).

18 | CALIBRATED DOSE LABELS

		$\rho_b = 0$			$\rho_b = 0.4$		$\rho_b = 0.8$	
	Pre-Calibration	Probit	Joint2d	Joint9d	Joint2d	Joint9d	Joint2d	Joint9d
d_1	-0.675	-6.514	-0.854	-4.334	-0.649	-3.572	-0.809	-1.119
d_2	-0.385	-6.469	-0.846	-4.281	-0.640	-3.525	-0.791	-0.569
d_3	-0.105	-6.423	-0.838	-4.227	-0.630	-3.477	-0.773	-0.259
d_4	0.151	-6.378	-0.829	-4.173	-0.620	-3.429	-0.755	0.017
d_5	0.385	-6.331	-0.821	-4.118	-0.610	-3.381	-0.736	0.327

TABLE 12 Dose labels before and after calibration, with calibration applied using a method-specific and ρ_b-specific approach.

19 | ESTIMATION PROBLEM WITH AND WITHOUT ALGORITHM SELECTION

	Separation		Equal estimated probability	
	nlminb	best	nlminb	best
S1	0.001	0	0.059	0.065
S2	0	0.001	0	0.001
S3	0.003	0.002	0.003	0
S4	0.044	0.061	0	0
S5	0.079	0.163	0.008	0.008

TABLE 13 The proportion of separation and the proportion of equal estimated probability for the Probit method with and without the algorithm selection. The column 'best' represents results with the algorithm selection selection.

	$\rho_b = 0$		$\rho_b = 0.4$		$\rho_b = 0.8$	
	nlminb	best	nlminb	best	nlminb	best
S1	0.001	0	0	0.002	0.003	0.003
S2	0.001	0	0	0	0.003	0
S3	0.009	0.001	0	0.008	0.008	0.006
S4	0.095	0.036	0.003	0.088	0.070	0.051
S5	0.224	0.082	0.005	0.161	0.133	0.061

TABLE 14 The proportion of separation for the Joint2d method with and without the algorithm selection. The column 'best' represents results with the algorithm selection selection.

	$\rho_b = 0$		$\rho_b = 0.4$		$\rho_b = 0.8$	
	nlminb	best	nlminb	best	nlminb	best
S1	0.063	0.089	0.072	0.123	0.076	0.221
S2	0.001	0.001	0	0.008	0.001	0.010
S3	0.002	0.004	0	0.003	0	0.014
S4	0	0	0.001	0.004	0.001	0.008
S5	0.009	0.026	0.015	0.021	0.011	0.050

TABLE 15 The proportion of equal estimated probability for the Joint2d method with and without the algorithm selection. The column 'best' represents results with the algorithm selection selection.

20 | ROBUSTNESS TO DATA GENERATION MECHANISM

The data generation process described in Section 5.1 assumes an AR(1) dependence structure among the repeated biomarker measurements, characterised by a variance-covariance matrix Σ_c as specified in Section 5.1. While this assumption offers parsimony and a natural interpretation of temporal decay in correlation, the reviewer rightly noted that AR(1) implies near-independence between distant measurements — for instance, with $\rho_c = 0.4$, the correlation between measurements at week 1 and week 8 is $\rho_c^7 \approx 0.002$. To evaluate the robustness of the proposed methods to this assumption, we consider an alternative data generation mechanism based on a random effects model, which induces a compound symmetry structure among the repeated biomarker measurements.

| Random effects data generation

Under the random effects model, the biomarker measurement for patient i at time point t and dose level d_j is modelled as:

$$Y_{ci,t}(d_j) = \mu_{ct}(d_j) + b_i + \varepsilon_{it}, \tag{15}$$

where $\mu_{ct}(d_j) = \beta_{c0}(d_j) + \beta_{c1} \cdot t$ is the dose- and time-specific mean with $\beta_{c1} = -1$ and dose-specific intercepts $\beta_{c0}(d_j)$ decreasing across dose levels; $b_i \sim N(0, \sigma_b^2)$ is a patient-level random effect shared across all eight measurement occasions, capturing the between-subject variability; and $\varepsilon_{it} \overset{iid}{\sim} N(0, \sigma_\varepsilon^2)$ are independent within-subject residual errors. This formulation implies a compound symmetry variance-covariance structure for the repeated biomarker measurements:

$$\Sigma_c^{RE} = \begin{pmatrix} \sigma_b^2 + \sigma_\varepsilon^2 & \sigma_b^2 & \cdots & \sigma_b^2 \\ \sigma_b^2 & \sigma_b^2 + \sigma_\varepsilon^2 & \cdots & \sigma_b^2 \\ \vdots & \vdots & \ddots & \vdots \\ \sigma_b^2 & \sigma_b^2 & \cdots & \sigma_b^2 + \sigma_\varepsilon^2 \end{pmatrix}, \tag{16}$$

where the within-subject correlation between any two measurements is constant regardless of their temporal distance:

$$\text{Corr}(Y_{ci,t}, Y_{ci,t'}) = \frac{\sigma_b^2}{\sigma_b^2 + \sigma_\varepsilon^2}, \quad \forall\, t \neq t'. \tag{17}$$

In contrast to the AR(1) model, where correlation decays exponentially as $\rho_c^{|t-t'|}$, the random effects model maintains a constant correlation between all pairs of measurements, irrespective of the time lag. This represents a substantially stronger and more uniform within-subject dependence structure, which may be more realistic for biomarkers whose trajectories are largely driven by stable patient-level characteristics.

The patient-level random effect b_i is constructed to be correlated with the underlying toxicity propensity u_{bi} of patient i, with correlation $\rho_b \in \{0, 0.4, 0.8\}$, preserving the same correlation structure between the biomarker and binary toxicity outcome as in the original data generation mechanism. Specifically:

$$b_i = \rho_b \cdot z_i \cdot \sigma_b + \sqrt{1 - \rho_b^2} \cdot \sigma_b \cdot \eta_i, \tag{18}$$

where z_i is the latent toxicity propensity for patient i and $\eta_i \overset{iid}{\sim} N(0, 1)$ is independent of z_i. The binary toxicity outcome is then generated as $Y_{bi} = 1[u_{bi} \le \pi(d_j)]$, where $u_{bi} = \Phi(z_i)$ and $\pi(d_j)$ is the true toxicity probability at dose d_j under scenario S. We set $\sigma_b = 1$ and $\sigma_\varepsilon = 0.5$, yielding a within-patient biomarker correlation of $\sigma_b^2/(\sigma_b^2+\sigma_\varepsilon^2) = 0.80$ under the random effects structure, substantially higher than the maximum pairwise correlation of $\rho_c = 0.4$ under AR(1).

| Simulation results

All other aspects of the simulation design - scenarios, sample size, initial stage, correlation values ρ_b, and operating characteristics - remain identical to those described in the main analysis. Simulation results under the random effects data generation are presented in Figures 23 and 24, and the same general patterns as in the main analysis are observed, with the following notable findings.

First, under the smaller initial stage (Initial1, Figure 23), all three methods exhibit substantially higher separation rates and lower PCS, particularly in the more challenging scenarios S_4 and S_5, consistent with the main manuscript findings that an insufficiently complex initial stage exacerbates the separation problem. Second, under Initial1, Joint2d and Joint9d show comparable PCS performance, with Joint9d offering only marginal improvement. This differs from the AR(1) setting and suggests that the stronger within-subject correlation induced by the random effects model provides sufficient additional information for Joint2d to perform comparably to Joint9d. Third, adopting the more complex Initial3 stage (Figure 24) leads to substantial improvement in PCS across all three methods, most pronounced in scenarios S_4 and S_5, which aligns with the main manuscript conclusion that a more complex initial stage can largely alleviate the separation problem and improve dose selection accuracy.

Overall, the simulation results under the random effects data generation are consistent with those in the main analysis, suggesting that the proposed methods are robust to the choice of dependence structure for repeated biomarker measurements. Notably, the comparable performance of Joint2d and Joint9d under Initial3 suggests that the marginal benefit of incorporating higher-dimensional biomarker information diminishes when a single informative biomarker already provides sufficient signal - a finding that has practical implications for trial design, where collecting fewer biomarker measurements may be preferable for simplicity and cost. A natural direction for future work is to investigate the performance of dose label calibration under the random effects data generation, which may yield further improvements in PCS and potentially enable the Probit and joint methods to achieve performance comparable to the Empiric benchmark, as demonstrated in the main analysis.

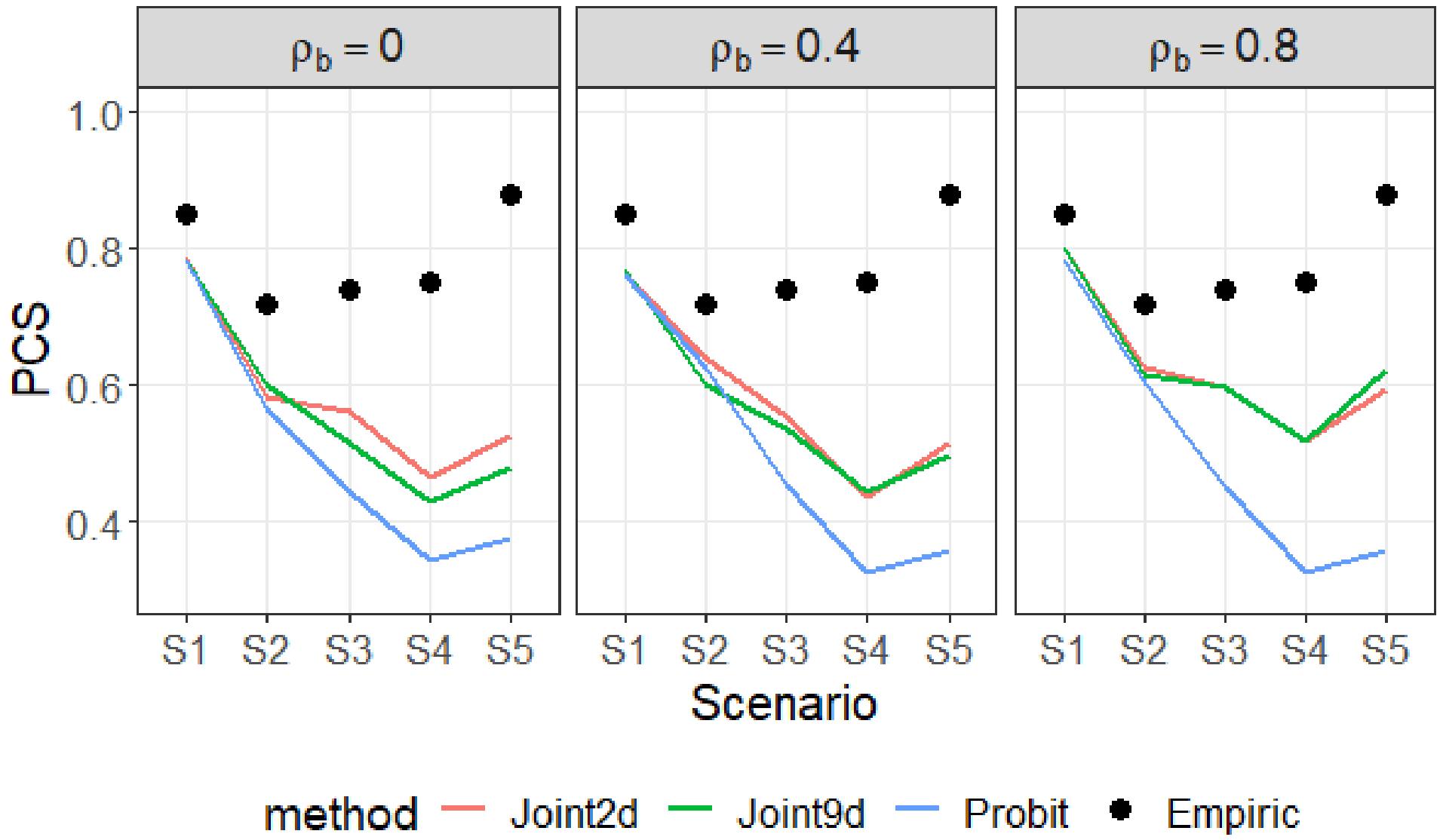

(a) PCS results across scenarios; Empiric method shown as points for reference ($S_1 - S_5$: 0.85, 0.72, 0.74, 0.75, 0.88).

	$\rho_b = 0$			$\rho_b = 0.4$		$\rho_b = 0.8$	
	Probit	Joint2d	Joint9d	Joint2d	Joint9d	Joint2d	Joint9d
S1	0.78 (0.24)	0.79 (0.20)	0.78 (0.20)	0.76 (0.21)	0.77 (0.22)	0.80 (0.18)	0.80 (0.18)
S2	0.57 (0.58)	0.58 (0.44)	0.60 (0.44)	0.64 (0.41)	0.60 (0.43)	0.63 (0.35)	0.61 (0.34)
S3	0.44 (0.60)	0.56 (0.46)	0.51 (0.47)	0.55 (0.42)	0.54 (0.45)	0.60 (0.33)	0.60 (0.33)
S4	0.34 (0.82)	0.46 (0.63)	0.43 (0.65)	0.44 (0.62)	0.44 (0.62)	0.52 (0.44)	0.52 (0.49)
S5	0.38 (0.83)	0.52 (0.66)	0.48 (0.71)	0.52 (0.66)	0.50 (0.67)	0.59 (0.55)	0.62 (0.54)

(b) PCS results; proportions of separation in parentheses. The Empiric method is excluded as separation does not arise under its one-parameter structure.

FIGURE 23 PCS results across five scenarios (S_1-S_5) and $\rho_b \in \{0, 0.4, 0.8\}$ with all three methods implementing the initial stage Initial1.
